# ﻿A comparative morphology of trichobothrial bases in araneoid spiders and its significance for the phylogeny and system of the superfamily Araneoidea (Arachnida, Araneae)

**DOI:** 10.3897/zookeys.1219.133002

**Published:** 2024-11-26

**Authors:** Kirill Y. Eskov, Yuri M. Marusik

**Affiliations:** 1 Borissiak Paleontological Institute, Russian Academy of Sciences, Profsoyuznaya Str., 123, Moscow 117647, Russia Borissiak Paleontological Institute, Russian Academy of Sciences Moscow Russia; 2 Institute of Biological Problems of the North, Far Eastern Branch, Russian Academy of Sciences, Portovaya Str., 18, Magadan 68500, Russia Institute of Biological Problems of the North, Far Eastern Branch, Russian Academy of Sciences Magadan Russia; 3 Department of Zoology and Entomology, University of the Free State, Bloemfontein 9300, South Africa University of the Free State Bloemfontein South Africa; 4 Altai State University, 61 Lenina Pr., Barnaul, RF-656049, Russia Altai State University Barnaul Russia

**Keywords:** Aranei, Araneomorphae, bothrial morphology, bothrial evolution

## Abstract

Bothrial morphology was studied by SEM in 137 araneoid genera representing all 22 currently recognized extant families and all 42 conventional subfamilies of the Araneoidea. The ancestral type in the superfamily Araneoidea is a ‘hooded’ bothrium with a single well-developed transverse ridge, dividing its proximal and distal plates (‘*Erigone*-type’); the advanced type is a solid dome-like bothrium without vestiges of the ridge (‘*Theridion*-type’); there are several intermediate types reflecting various pathways and stages of the ridge reduction (united here as ‘*Argiope*-type’). The parallel trends in bothrial evolution, recognized as continuous series from the ancestral type up to the advanced one through some intermediate stages, are distinguished in each of the seven main phylogenetic lineages of the superfamily: ‘tetragnathoids’, ‘araneoids’, ‘cyatholipoids’, and ‘theridioids’ possess a complete set of the three types, while ‘malkariods’, ‘symphytognathoids’. and ‘linyphioids’ lack the advanced, dome-like type (‘*Theridion*-type’). Only three taxa have been proposed earlier as the sister group of the superfamily Araneoidea: Nicodamoidea, Deinopoidea, and Leptonetoidea; morphology of bothria, as well as other cuticular microstructures, clearly supports the araneoid-nicodamoid relationship hypothesis, purely ‘molecular’ to date. Bothrial morphology provides the additional arguments for several taxonomic acts, e.g., for the reranking the Agnarsson’s (2004) ‘clade 35’ (*Theonoe*, *Carniella*, *Robertus*, and *Pholcomma*) up to the Theonoeinae Simon, 1894, **stat. nov.**, and for the revalidation the micropholcommatid *Plectochetos* Butler, 1932, **gen. revalid.** and zygiellid *Parazygiella* Wunderlich, 2004, **gen. revalid.**

## ﻿Introduction

Trichobothria are a conspicuous type of mechanoreceptive sensilla in terrestrial arthropods. They are represented by erect, very elongated setae that are set in a deep cup-like socket, the bothrium. The morphology of trichobothrial bases is very diverse in spiders and has become a popular subject of investigation since the very beginning of regular usage of scanning electron microscopy in taxonomy and phylogeny of the order. For instance, trichobothrial characters were successfully used in the diagnostics of suprageneric taxa and reconstruction of evolutionary trends in mygalomorphs by Raven (1985) and Eskov et al. (2024), in ‘hypochiloids’ by Forster et al. (1987), and particularly in dionychans by Ramírez (2014).

However, the largest superfamily of the order, Araneoidea, which comprises 22 extant families and more than a quarter of the described species, was almost ignored by arachnologists in this respect: araneoid trichobothria are highly uniform and therefore seem useless for high-level (suprageneric) systematics and phylogeny. It is indicative that the fundamental ‘Atlas of phylogenetic data for entelegyne spiders’ by Griswold et al. (2005) presents SEM images of bothria of 49 species of 23 families, but only one of them (!) represents the araneoids (the mimetid *Mimetushesperus* Chamberlin, 1923: fig. 149 G); the diagnosis of the superfamily contains only a laconic remark: “...Trichobothrial bases are smooth” (Griswold et al. 2005:14).

The review of Simphytognathoidea by Lopardo and Hormiga (2015) comprises 23 characters concerning, one way or another, the trichobothria, but only one of them touches the morphology of the trichobothrial shaft (“smooth/serrated” or “distinctly plumose”: char. 150), and not a single one considering the morphology of the bothria. Álvarez-Padilla and Hormiga (2011: 713) declared a special attention to the trichobothria in their review of tetragnathid morphology: “We study the evolution of six morphological character systems within Tetragnathidae: spinneret spigots, respiratory structures, trichobothria, chelicerae, and male and female genitalia”; however, they presented numerous SEM images trichobothrial pattern of the legs, but not a single enlarged image of the bothria permitting to discern its structure.

Even in cases where arachnologists have paid attention to this structure and described the bothria of the studied araneoid taxa in detail, e.g., Forster et al. (1990) in Synotaxidae s. l., Rix and Harvey (2010) in Micropholcommatidae, they have not attempted any generalization. So, an evolutionary ‘big picture’ of the development of these structures in Araneoidea, comparable with abovementioned conclusions by Ramírez (2014) for Dionycha, is completely absent.

The only case of an interfamilial comparison of bothrial morphology in araneoids was provided by Miller (2007), in a study of synaphrid relationships; only two bothrial types were distinguished by him, ‘hooded’ and those that are ‘evenly rounded and lack a hood’: “The typical bothrium form in Synaphridae is hooded, a characteristic shared at least with Anapidae (Platnick and Forster 1989: fig. 15), Micropholcommatidae (Forster and Platnick 1984: figs 374, 375), Mysmenidae (Griswold 1985: fig. 8), Theridiosomatidae (unpublished data), Malkaridae (Platnick and Forster 1987: fig. 18), Linyphiidae (Hormiga 2002: fig. 46G), Synotaxidae (Forster et al. 1990: figs 144, 260), and many non-araneoid spider families (Forster and Platnick 1984; Griswold et al. 2005); bothria in other araneoid families including araneids (Griswold et al. 1998: fig. 22E), theridiids (Agnarsson 2004: figs 31G, 69E, 74D), cyatholipids (Griswold 2001: fig. 6B), and mimetids (Griswold et al. 2005: fig. 149G) are evenly rounded and lack a hood” (Miller 2007: 30).

Distribution of these two bothrial types through the listed araneoid families seems completely chaotic and lacking any phylogenetic sense; Miller (2007) has not formulated such a conclusion directly, but it is quite obvious. And probably due to this deceptive ‘obviousness’, subsequent investigators lost any interest in this problem and turned away from searching for any regularities in this field.

We, however, suspected that the above ‘phylogenetic chaos’ was an artifact, caused by the following factors: (1) a too rough typology of bothria (two opposite types only); (2) a too limited number of studied araneoid species/genera; and (3) a too random set of the studied araneoid families/ subfamilies (the latter two because of a deficiency of his own or published data available to Miller (2007) at that time).

Testing this supposition, we have studied the bothrial morphology in 137 araneoid genera representing all 22 currently recognized extant araneoid families and all 42 conventional subfamilies; so, a complete (or close to complete) diversity of these structures through superfamily Araneoidea seems to have been revealed. Araneoid bothria turned out to be much more uniform than, e.g., dionychan (see Ramírez 2014), but the ‘fine-tuning’ of bothrial typology indicated some bothrial types in addition to the above-mentioned couple ‘hooded’ (basal) and ‘evenly rounded’ (terminal).

Forster (1988: 11), in his study of the Cyatholipidae of New Zealand, noted in the family diagnosis: “Bothria with the posterior hood reduced to two small ridges or absent”, and then guessed the following: “The reduction of the posterior hood of the bothrium and the small aperture of the domed tarsal organ are all derived characters which have developed apparently in parallel in many of the families” (Forster 1988: 15). And the ‘big picture’ of bothrial evolution in various Araneoidea lineages revealed in this work convincingly supports this assumption.

## ﻿Material and methods

SEM images were taken on a Tescan Vega2 and a Tescan Vega3 scanning electron microscopes in Palaeontological Institute (Moscow), operated in a high vacuum mode at the accelerating voltages of 10–20 kV, using SE and BSE detectors. Specimens were gradually dehydrated in 100% ethanol, dried, and sputter-coated with gold-palladium.

The bothria of 142 araneoid and five non-araneoid species are figured; in addition, the original images of three araneoid bothria were obtained from colleagues. For a list of the specimens examined, see Suppl. material 1. Terminology of the bothrial parts follows that in Eskov et al. (2024). All measurements are given in μm. Abbreviations of leg joints: **mt** – metatarsus, **ti** – tibia.

Abbreviations for trichobothria parts: ***al*** alveolus; ***AP*** angle with clear apex of proximal plate; ***dp*** distal plate; ***ff*** frontal fold; ***pp*** proximal plate; ***pp+dp*** fused proximal and distal plates; ***RA*** round apex; **SbA** semicircular arch; **sh** shaft; **tr** transverse ridge of proximal plate.

## ﻿Results

### ﻿Preliminary notes on the sister group of the superfamily Araneoidea

Three phylogenetically distant taxa have always been supposed to be the sister group of the superfamily Araneoidea:

1. Nicodamoidea: cribellate Megadictynidae Lehtinen, 1967 and ecribellate Nicodamidae Simon, 1897. “The Nicodamidae is one of the few spider families for which there is no well supported hypothesis on its position” (Jocqué and Dippenaar-Schoeman 2006: 184: fig. 5).
2. Deinopoidea: Deinopidae C.L. Koch, 1850 and Uloboridae Thorell, 1869. “The entirely cribellate Deinopoidea [...] All spin modified orbs. Some controversy existed in the past over placement of Deinopidae (ogre-faced spiders), but ethological work showed that they shared derived motor patterns unique to orb weavers, despite the derived web architecture” (Coddington and Levi 1991: 585). Deinopoids were coupled with another orb-web builders, ecribellate araneoids, in frame of the concept ‘Orbicularia’ (Coddington 1986a; Jocqué and Dippenaar-Schoeman 2006: fig. 5).
3. Leptonetoidea (sensu Wunderlich and Müller 2018): cribellate Archoleptonetidae Gertsch,1974, ecribellate Leptonetidae Simon, 1890, and Telemidae Fage, 1913 (all extant), cribellate Protoaraneodidae Wunderlich & Müller, 2018 and ecribellate Praeterleptonetidae Wunderlich, 2008 (both extinct). The sister pair Leptonetidae + Telemidae was traditionally nested within Haplogynae (Platnick et al. 1991; Jocqué and Dippenaar-Schoeman 2006: 150: fig. 4). However, “The distribution of several features of the spinning organs [in recently discovered the cribellate leptonetids], respiratory system, and genitalia suggests that the phylogenetic position of the Leptonetidae needs to be reevaluated and makes their position within the Haplogynae uncertain” (Ledford and Griswold 2010: 2). Finally, the new molecular data (Wheeler et al. 2017) placed leptonetids far outside the robustly supported Synspermiata (all the ecribellate ‘haplogynes’, including the telemids).

The hypothesis of the araneoid-nicodamid relationship (as well as the incorporation of the cribellate *Megadictyna* Dahl, 1906 in the ecribellate Nicodamidae) was first put forward by Forster (1970). The trichobothrial pattern of the legs was listed as one of the main characters suggests the affinities of the Nicodamidae with the Araneoidea (Forster 1970: 177): single trichobothrium on each of the metatarsi I–III, trichobothria absent on metatarsus IV and absent on tarsus.

However, this hypothesis was not accepted: Harvey (1995) nested Nicodamidae within the ‘RTA-clade’, close to Titanoecidae, and rejected the araneoid-nicodamid relationship. One of his main objections was the dissimilarity of the spinning organs: “The identification of synapomorphies for the Orbiculariae and Araneoidea by Coddington (1990a, 1990b) conclusively excludes the Nicodamidae from these groups, particularly as nicodamids possess two major ampullate spigots on the anterior lateral spinnerets [Fig. 11], whereas all orbicularians possess only one” (Harvey 1995: 283). Regarding the simplified trichobothrial pattern (the lack of tarsal trichobothria and the presence of only a single metatarsal trichobothrium) in both araneoids and nicodamids, Harvey (1995: 287), following Coddington and Levi (1991), considered it “plesiomorphic within the Araneomorphae” and, consequently, insufficient (see discussion of the polarity of this character below).

The concept of ‘Orbicularia’ became a step in another direction: “Reconstitution of the orb weavers, or Orbiculariae, also resulted from cladistic analysis of a classical cribellate-ecribellate dichotomy. Classically orb webs were thought to have evolved twice: once among the (paraphyletic) Cribellatae, and once among the (polyphyletic) Ecribellatae. […] Given the collapse of the Cribellatae and Ecribellatae as valid taxa, the orb web itself constituted initial evidence for monophyly. A series of detailed ethological and morphological investigations has failed to refute this hypothesis, thus corroborating that cribellate orb weavers (Deinopoidea) are the sister group of Araneoidea” (Coddington and Levi 1991: 584–585). This concept was put forward by Coddington (1986a, 1990a, b) and was widely accepted and prevailed for the next two decades (e.g., Griswold et al. 1998, 1999; Jocqué and Dippenaar-Schoeman 2006). It should be noted that almost all morphological characters supporting the ‘Orbicularia’ as a monophyletic clade seem limited by the fine structure of the spinning organs (see Griswold et al. 2005).

Nowadays, Forster’s (1970) earlier assumption on the araneoid-nicodamoid relationship (as well as the unity of cribellate and ecribellate nicodamoids) was resurrected by molecular methods and is accepted in most modern phylogenies (see Kallal et al. 2018: fig. 1B–F). “Our data refute the long-held paradigm of orbicularian monophyly [...] by including the RTA clade in the same lineage that groups the cribellate (Deinopoidea) and ecribellate (Araneoidea) orb-weavers. This latter result, based on DNA sequence data, is by no means new [...], but has been dismissed repeatedly in favour of the orbicularian monophyly hypothesis. [...] The results presented herein suggest that nicodamids are the closest relatives to a clade that includes all ecribellate orb-weavers” (Dimitrov et al. 2017: 231–234).

It is not surprising that this result “has been dismissed repeatedly”, because the ‘molecular clade’ Nicodamoidea + Araneoidea still lacks any sufficient morphological support: “Morphological evidence for this arrangement remains weak [...] The morphological evidence for placing nicodamids near or far from orb-weavers is not robust. It is molecular evidence, albeit from the same genes but with a diverse array of taxon samples, that strongly associates Nicodamoidea with Araneoidea” (Dimitrov et al. 2017: 240).

Wunderlich and Müller (2018) recently proposed their ‘Leptonetoidea’ (i.e., well-known haplogyne couple Leptonetidae + Telemidae, supplemented with several extinct haplogynes, both ecribellate and cribellate) as the Araneoidea sister group. The ‘Leptonetoid-Araneoid branch’ was placed by Wunderlich and Müller (2018: fig. 1) near the base of araneomorphs and characterized by the following characters: “large and erect paracymbium; loss of feathery hairs; tendencies to existence of lateral cheliceral files and the loss of the cribellum”. All the listed characters of the supposed branch seem ambiguous: several leptonetoids (both extant and extinct) possess the cribellum; the majority of araneoids lack the cheliceral files; the homology of the araneoid paracymbium and the leptonetoid cymbial outgrowth was not proved; and the loss of feathery hairs seems to be rather a homoplasy than a synapomorphy. In addition, Wunderlich and Müller (2018: 41) pay special attention to the leg autotomy, but this character in any case is not a synapomorphy of ‘Leptonetoid-Araneoid branch’: on the one hand, it was not recorded in the majority of araneoids, and on the other hand it was recorded in the very distantly related Filistatidae and Herseliidae (Scharff and Coddington 1997: char. 36).

Before testing these three hypotheses by the characters of cuticular microstructure (including the bothrial morphology, ignored previously in this respect) let us return to the above-mentioned trichobothrial pattern of the legs. Conclusion on the polarity of this character in the araneomorphs is still based on Lehtinen’s early hypothesis: “Type I. The plesiomorphic pattern of trichobothria in spiders consists of a single subdistal metatarsal and two parallel rows of tibial trichobothria, but none on tarsi or femora” (Lehtinen 1980: 493), and “The simplest trichobothrial pattern, with two longitudinal parallel rows dorsally on tibiae and a single row on metatarsi, is shared by most Araneoidea and related groups, most haplogyne groups, some Nicodamidae and Eresidae. The trichobothrial pattern of most theraphosomorph spiders [i.e., Liphistiomorphae and Mygalomorphae] is much more complicated [i.e., with tarsal and several metatarsal trichobothria]. However, I regard the latter as an apomorphic pattern, and probably the simple pattern described above is close to an ancestral pattern” (Lehtinen 1978: 265).

There was no discussion as to why he chose to “regard the latter as an apomorphic pattern”. Nevertheless, this viewpoint was supported, without any additional arguments, by Coddington and Levi (1991: 581–582): “The distribution of trichobothria (fine sensory hairs) on the metatarsi and tarsi is another important character [...]. The plesiomorphic araneomorph pattern seems to be [the] absence or near absence on the metatarsi and tarsi (although present on mygalomorph and liphistiomorph tarsi). The derived condition is single or multiple rows of trichobothria”, and this hypothesis persists until now (e.g., Miller et al. 2010). It should be emphasized that for the mygalomorh trichobothrial pattern Coddington and Levi (1991: 575) adhered (without any special argument again) to the opposite polarity: “It is interesting that one apomorphy of the atypoids is the great reduction or absence of tarsal trichobothria”.

In our opinion, an obvious phylogenetic protocol of comparison of the Araneomorphae with its sister group, Orthognatha (Liphistiomorphae and Mygalomorphae), assumes the opposite polarity of this character: just the ‘complex pattern’ (with tarsal and several metatarsal trichobothria) is an ancestral condition, directly inherited by the araneomorphs from the orthognathans. This pattern has persisted in some araneomorph lineages (e.g., RTA-clade and Salticidae), or partially reversed in others: e.g., the appearance of the tarsal trichobothria in haplogyne Caponidae (Jocqué and Dippenaar-Schoeman 2006: 88), and of the additional metatarsal trichobothria in some unrelated araneoids, such as the araneid *Melychiopharis* Simon, 1895 (Fig. 12A) and linyphiid *Allomengea* Strand, 1912 (Helsdingen 1974).

A further simplification of the initial ‘complex’ pattern in various araneomorph lineages (due to parallel reductions) is a more easily explained evolutionary trend than a complication of the initial ‘simple’ pattern (due to numerous parallel origins). Thus, Scharff and Coddington (1997: char. 37) supposed the abovementioned multiplication of the metatarsal trichobothria in several unrelated araneid genera (cyrtarachnin *Mastophora* Holmberg, 1876, gasteracanthin *Gasteracantha* Sundevall, 1833, aranein *Cyclosa* Menge, 1866, etc.) were autapomorphies, but reversions to the common plesiomorphic/ancestral condition seem more convincing. So, the single ‘simplified’ type of trichobothrial pattern in both nicodamoids and araneoids was correctly considered by Forster (1970) as evidence of their relatedness.

Regarding the morphology of trichobothrial bases (as well as the remaining cuticular microstructures) Lehtinen (1996: 407) concluded: “The leg skin structure of Amaurobiomorpha and the primitive outgroups (Hypochilidae, Gradungulidae and Austrochilidae) as well as all true labidognath Haplogyne groups (Dysderoidea, Scytodoidea and Caponiidae) is either ridged or secondarily smooth, while the longitudinally ridged bothrial base is dominant throughout these lines of evolution, strongly suggesting the plesiomorphic state of the ridged skin and longitudinally ridged bothrial base in labidognath spiders”. The same polarity of this cuticular character, i.e., from the fingerprint (ridged) leg cuticle to the scaly via the smooth ones, was stated by Ramírez (2014: char. 100), and we also agree with this conclusion.

The ridged cuticle is absent in all of the most archaic spider taxa (Liphistiomorphae, Mygalomorphae, and Filistatidae) and seems a synapomorphy of the ‘non-filistatid araneomorphs’; the ‘longitudinally ridged bothria’, in an obvious way, arise from the surrounding ridged cuticle. This cuticular/bothrial type is really plesiomorphic for the suborder, being present in all basal ‘non-filistatid araneomorphs’, as all ‘hypochiloid’ lineages (e.g., Hypochilidae: Fig. 2F) and Synspermiata (e.g., Ochyroceratidae: Brescovit et al. 2018: fig. 6A). The cuticle/bothria in Leptonetoidea (Fig. 2E) and Deinopoidea (Fig. 2C) belong to this plesiomorphic type. In addition, “Ridged skin is correlated with the presence of several [non-serrate] types of hairs” (Lehtinen 1996: 399), which also appears plesiomorphic (e.g., Uloboridae: Fig. 2D).

In advanced Araneomorph lineages, the ridged cuticle is replaced by a scaly (or secondarily smooth) one; plumose and pseudoserrate (plumose-laminar) setae are replaced by serrate ones; and longitudinally ridged bothria are replaced by transversally ridged (or smooth dome-like) ones. Scaly cuticles and serrate setae are conventionally listed as synapomorphies of Araneoidea (e.g., Griswold et al. 1998; Ramírez 2014), and these characters are shared with Nicodamidae (Fig. 2B). The bothria of Nicodamoidea, both Nicodamidae (Fig. 2A) and Megadictynidae (Griswold et al. 2005: fig. 154F), are non-longitudinally ridged, as well as of all the araneoid ones (Figs 3A–F, 4A–F).

To sum up: in addition to (1) the ‘simplified’ trichobothrial pattern (no tarsal, a single metatarsal), both Araneoidea and Nicodamidae share the following characters: (2) serrate (not plumose) setae; (3) scaly (not ridged) leg cuticle; and (4) trichobothrial bases not longitudinally ridged; all of these are derived characters. These characters stated here in the set of morphological synapomorphies, support the ‘purely molecular’, until now, clade Nicodamoidea + Araneoidea. It should be noted that Lehtinen (1996: 399) doubted a close relationship of nicodamids with their scaly skin and serrate setae and megadictynids with their smooth skin and plumose-laminar setae (see Lehtinen 1996: fig. 46; Griswold et al. 2005: fig. 137A, B); however, the cribellate *Megadictyna* may be supposed a basal sister group of all the remaining, ecribellate, members of the above clade.

It should be noted that there are two directions of the initial transformation of the ‘bothrial hood’, leading to the two opposite derived bothrial types: the ‘dome-like’ (see details below) and the other, here termed ‘multiridged’: “Trichobothria proximal plate transverse ridges: 0. Smooth. The hood is smooth, without definite transverse ridges; it may have similar sculpture as the surrounding cuticle [...]. 1. With transverse ridges. The hood has well-defined transverse ridges [...]. These ridges are much larger than the sculpture of the surrounding cuticle” (Ramírez 2014: char. 178).

The multiridged bothria are absent in all the basal Araneomorphae (‘hypochiloids’, Filistatidae and Synspermiata), and present in all the advanced araneomorph clades (Wheeler et al. 2017: figs 3–8): Palpimanoidea (e.g., Huttoniidae), ‘RTA-clade’ (e.g., Amaurobiidae), ‘Oval calamistrum clade’ (e.g., Zoropsidae), Dionycha (e.g., Trachelidae) (see Ramírez 2014: figs 94E, F, K, 96C, respectively). The only advanced araneomorph clade completely lacking this advanced bothrial type is Araneoidea + Nicodamidae; at the same time, Megadictynidae possesses the multiridged bothria (Griswold et al. 2005: fig. 154F), and it seems an apomorphy of the basal cribellate lineage of this clade.

**Figure 1.**
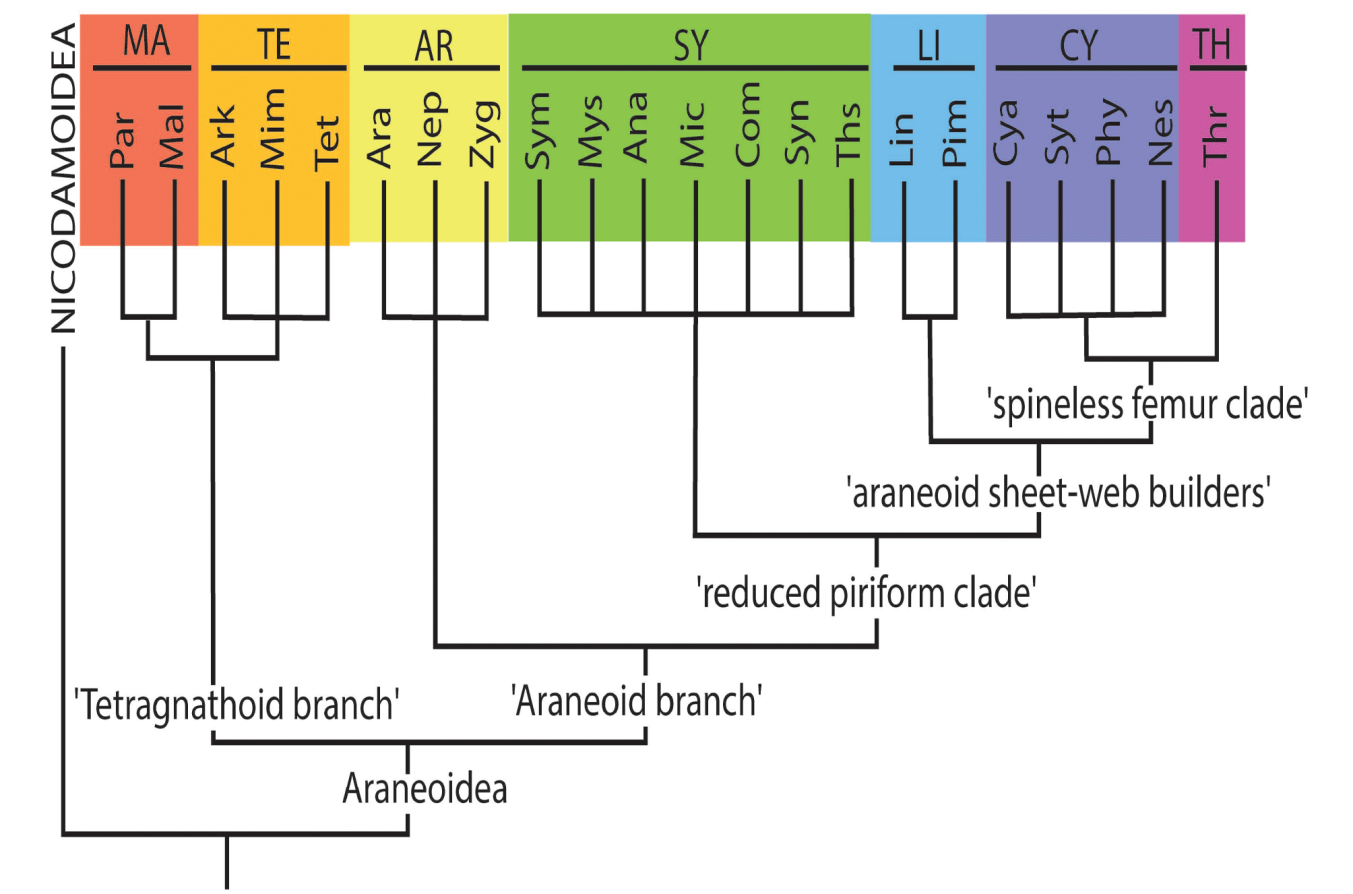
Accepted system of the superfamily Araneoidea, down to families (by Eskov and Marusik 2023). Abbreviations: AR – Araneoid lineage (Ara – Araneidae, Nep – Nephilidae, Zyg – Zygiellidae); CY – Cyatholipoid lineage (Cya – Cyatholipidae, Nes – Nesticidae, Phy – Physoglenidae, Syt – Synotaxidae); LI – Linyphioid lineage (Lin – Linyhiidae, Pim – Pimoidae); MA – Malkaroid lineage (Mal – Malkaridae, Par – Pararchaeidae); SY – Symphytognathoid lineage (Ana – Anapidae, Com – Comaromidae, Mic – Micropholcommatidae, Mys – Mysmenidae, Sym – Symphylognathidae, Syn – Synaphridae, Ths – Theridiosomatidae); TE – Tetragnathoid lineage (Ark – Arkyidae, Mim – Mimetidae, Tet – Tetragnathidae); TH – Theridioid lineage (Thr – Theridiidae).

In other words, bothrial morphology, as well as the morphology of the remaining cuticle microstructures, clearly support the nicodamoid-araneoid relationship hypothesis, and rejects the two competing ones (i.e., deinopoid-araneoid and leptonetoid-araneoid relationship).

### ﻿Preliminary notes on the typology of the trichobothrial bases in Araneoidea

**Figure 2.**
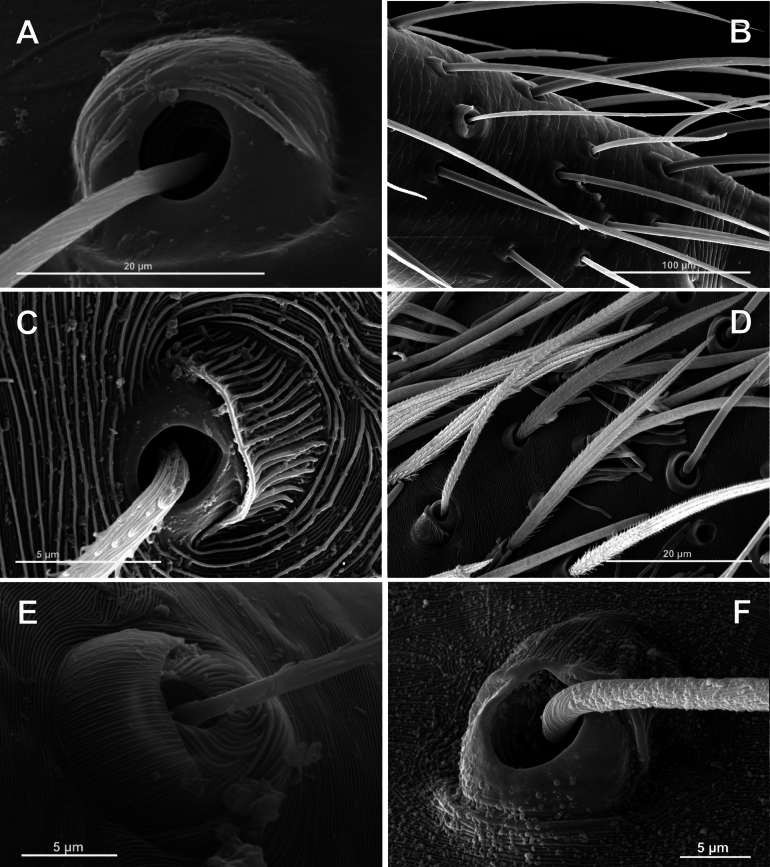
Bothria of the supposed sister groups of Araneoidea (**A, B**Nicodamoidea: Nicodamidae**C, D**Deinopoidea: Uloboridae**E**Leptonetoidea: Leptonetidae) and of the basal Araneomorphae (**F** ‘Hypochloidea’: Hypochilidae) **A***Litodamushickmani*, ti 3 **B***Litodamushickmani*, mt 3 **C***Myagrammopis* sp. ti 2 **D***Zozis* sp., ti 3 **E***Leptonetelacaucasica*, mt 1 **F***Hypochiluspococki*, ti 3.

The structure of spider trichobothria and the names of their parts is given following Ramírez (2014: 122–125): “The trichobothria […] are sensory setae on the dorsal surfaces of legs and palps, specialized in detecting air movement. The *setal shaft* is slender, perpendicular to the cuticle surface [...]. The socket forms a cup or *bothrium*, with an ample central cavity. The opening of the cup (*alveolus*) restricts the movement of the *setal shaft*. The *bothrium* is usually divided in *proximal* and *distal plates*; the *proximal plate* is often called *trichobothrial ‘hood*’.” The bothria of nicodamids, confirmed herein as a sister group of araneoids, is ‘hooded’ (Fig. 2A, B; Harvey 1995: fig. 6; Lehtinen 1996: fig. 45): its proximal plate, the ‘trichobothrial hood’, is clearly differentiated from the distal plate (see Ramírez 2014: fig. 84D). So, the ‘hooded’ bothria should be recognized as the ancestral type in the superfamily Araneoidea.

**Figure 3.**
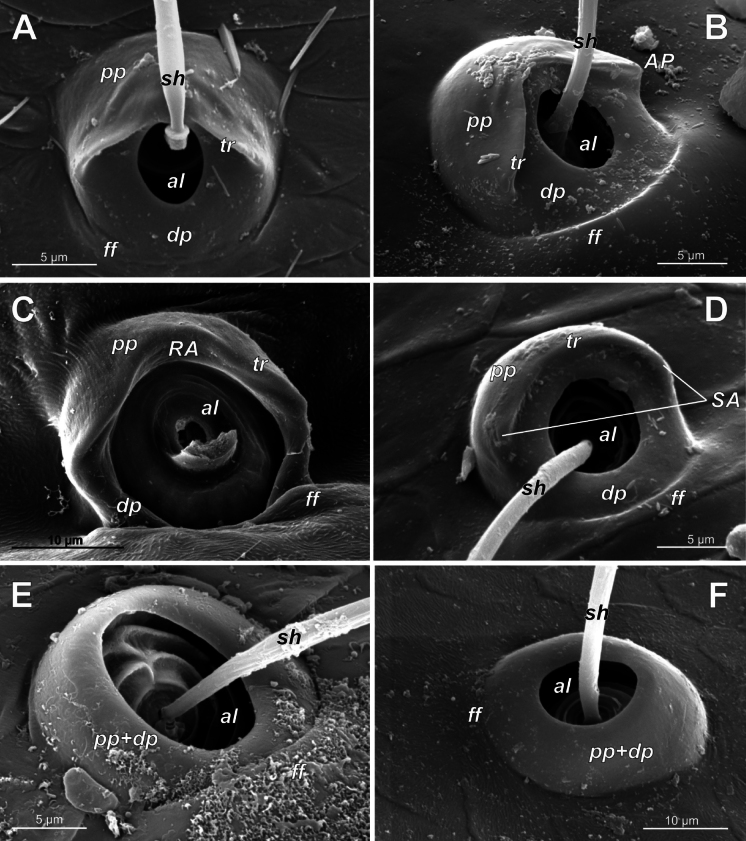
Typology of araneoid bothria (I). **A–D** ‘E-type, *Erigone* type’ (ancestral) **E, F** ‘T-type, *Theridion* type’ (advanced) **A***Erigonedentipalpis*, (E-type) **B***Chilenodesaustralis* (E-type) **C***Chrysometaalajuela* (E-type) **D***Melychiopharis* sp. (E-type) **E***Theridiontinctum* (T-type) **F***Nephila* sp. (T-type). Abbreviations: *al* alveolus; *AP* angle with clear apex of proximal plate; *dp* distal plate; *ff* frontal fold; *pp* proximal plate; *pp+dp* fused proximal and distal plates; *RA* round apex; *SA* semicircular arch; *sh* shaft; *tr* transverse ridge of proximal plate.

An ancestral (‘hooded’) araneomorph bothrium consists of the following structures: a more or less flattened distal plate (dp) with a rounded opening for a setal shaft (alveolus: al); a more or less swollen proximal pate (‘hood’: pp) with its distal margin forming a clear transverse ridge (tr); and a cuticular fold that delimits the bothrium from the front, termed here the ‘frontal fold’ (ff) (see Fig. 3A–D).

A single high-rank spider clade where the bothrial transformations have been studied in detail and on a large scale is Dionycha: “Trichobothria proximal and distal plate limit: 0. Well differentiated. The distal margin of the trichobothrial hood is well defined, often overhanging the distal plate and the opening of the socket [...]. In some cases, the margin is well marked, although not overhanging [...]. 1. Not well differentiated. The distal margin of the hood is tenuous, superficial, not well marked [...]. 2. Homogeneous. The bothrium is smooth, without distinction into proximal and distal plates [...]. States are ordered, as state 1 is intermediate between states 0 and 2” (Ramírez 2014: char. 176).

**Figure 4.**
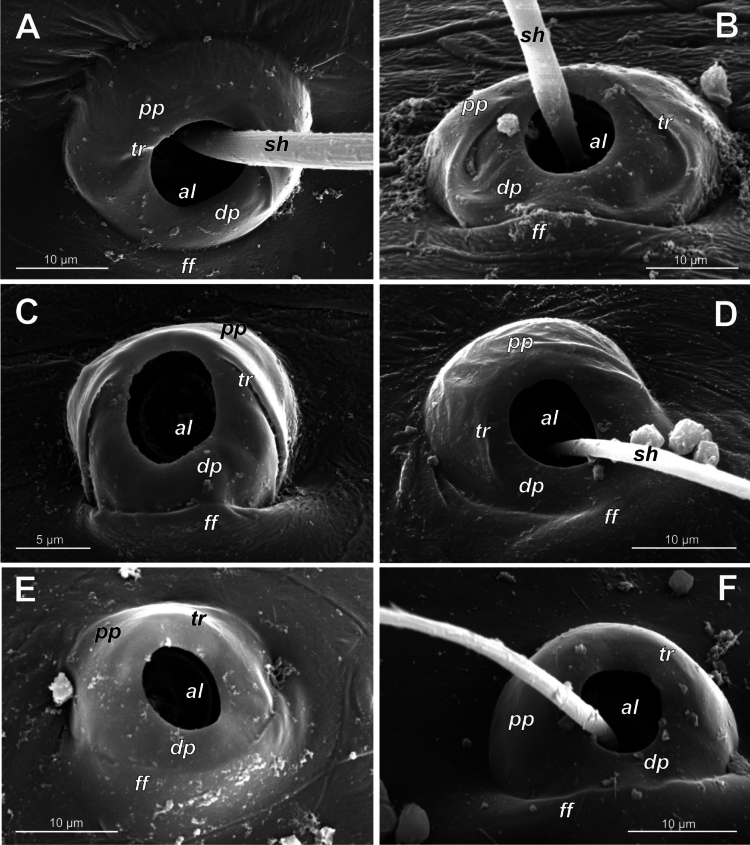
Typology of araneoid bothria of the *Argiope* type (intermediate) (II) **A***Argiopebruennichi***B***Nanometa* sp. **C***Synotaxus* sp. **D***Stemonyphanteslineatus***E***Erofurcata*, leg 2 tibia **F***Eidmannellapallida*. Abbreviations: *al* alveolus; *dp* distal plate; *ff* frontal fold; *pp* proximal plate; *pp+dp* fused proximal and distal plates; *sh* shaft; *tr* transverse ridge of proximal plate.

The trend in the araneoid bothria transformation seems exactly like this. The ancestral type in the superfamily Araneoidea is a ‘hooded’ bothria with a single well-developed transverse ridge, dividing its proximal and distal plates, named herein ‘*Erigone*-type’ (Fig. 3A–D); the advanced type is a solid dome-like (“homogeneous”, “evenly rounded and lack a hood”) bothria without vestiges of the ridge: ‘*Theridion*-type’ (Fig. 3E, F); and there are several intermediate cases reflecting various pathways and stages of the ridge reduction, all united herein as the ‘*Argiope*-type’ (Fig. 4A–F).

Several subtypes in each of the three above types may be recognized. However, in, e.g., the ‘*Erigone*-type’ we face a continuous series of a bothria ridge configuration, from an angle with a clear apex (*AP*, Fig. 3B) to a semicircular arch (*SA*, Fig. 3D), via an angle with a rounded apex (*RA*, Fig. 3C). The regularities of a gradual ridge reduction in the ‘*Argiope*-type’ strictly correspond to those described in dionychans: “Trichobothria proximal and distal plates medial differentiation: 0. Hood entire, differentiated [...]. 1. Hood not differentiated medially. The distal margin of the hood is only marked at the sides [...]” (Ramírez 2014: char. 177). The disappearance of a ridge, if it occurs, always begins from its medial portion (Fig. 4C, D), and at the edges of this gradient, bothria hard to classify may be found; is it (Fig. 4B) already the ‘*Argiope*’ or yet the ‘*Erigone*’ type? Is it (Fig. 4E, F) yet the ‘*Argiope*’ or already the ‘*Theridion*’ type? Ramírez (2014: char. 178) has faced such a problem too: “Some terminals had intermediate or ambiguous conditions”, and it is an objective irremovable difficulty of any typological procedure.

However, let us try to formalize the ‘dividing lines’ between the recognized here three main bothrial types: the ancestral ‘*Erigone*-type’, the advanced ‘*Theridion*-type’, and the intermediate ‘*Argiope*-type’. We count a bothrium among the ancestral ‘*Erigone*-type’ if its transverse ridge, angled or rounded, persists an unbroken (vs a ridge distinctly erased in at least its medial portion, as in, e.g., Fig. 4B). And we count a bothrium among the advanced ‘*Theridion*-type’ type if it is a correct dome without any vestiges of a ridge and with an alveolus is present strictly at the dome top (vs an alveolus persists at the more or less fattened frontal portion of a dome corresponding to a distal plate, as in e.g., Fig. 4F).

Thus, a simplification seems a general trend in bothrial evolution in araneoids, as well in dionychans (Ramírez 2014) and in mygalomorphs (Eskov et al. 2024); its final point is the bothria with the initial structures fused in one dome.

### ﻿Distribution of various bothrial types in various lineages of Araneoidea

The system of Araneoidea adopted in this study was proposed by Eskov and Marusik (2023) as a reconciliation of the ‘morphological’ and ‘molecular’ cladograms of the superfamily by Griswold et al. (1998) and by Dimitrov et al. (2017). The higher-rank Araneoidea subtaxa (‘branches’ and ‘lineages’) are listed below in the systematic order corresponding to the superfamily cladogram (Fig. 1), whereas the lower-rank subtaxa (the families in a ‘lineage’ and subfamilies/tribes in a family) are listed in alphabetical order. The names and abbreviation of the suprageneric taxa are listed according to their ranks, in the text below, in the list of specimens examined (Suppl. material 1), as well as in the cladograms, are as follows: ‘branches’ (four capital letters), ‘lineages’ (two capital letters), families (three lowercase letters, in round brackets) and subfamilies/tribes (five lowercase letters, in brackets). If the conventional division of a family to subfamilies/tribes is not established, we conditionally list all its members as a ‘nominative subfamily’ (marking it by the usual five lowercase letters in brackets).

‘**Tetragnathoid branch’, TETR**

1. ‘Malkariod lineage’, MA

1.1. Malkaridae Davies, 1980 (Mal).

1.1.1. Malkarinae Davies, 1980 [Malka].

1.1.2. Sternoidinae Moran, 1986 [Stern].

1.1.3. Tingotinginae Hormiga & Scharff, 2020 [Tingo].

1.2. Pararchaeidae Forster & Platnick, 1984 (Par). Conventional subfamilies/tribes are not established [Parar].

Malkaroids were listed in former times in various, very distant, spider superfamilies: Palpimanidae, Zodariidae, Archaeidae, Mecysmaucheniidae, Mimetidae, and Araneidae (see review in Hormiga and Scharff 2020). Finally, Dimitrov et al. (2017) based on molecular data, united pararchaeids with malkarids in a common lineage and nested it into the superfamily Araneoidea as the sister group of ‘tetragnatoids’; the family Pararchaeidae was ranked by them as a subfamily of Malkaridae.

However, pararchaeids are distinguished from all other malkarids by a number of important apomorphies (i.e., the elevated chelicerae arising from a distinct, fully sclerotized foramen in the prosoma; pars cephalica steeply elevated from pars thoracica above the level of coxae III or IV; the presence of cheliceral peg teeth and the absence of a tarsal claw on the female pedipalp). Due to the fact that both taxa “turned out to be reciprocally monophyletic” (Hormiga and Scharff 2020: 348), Eskov and Marusik (2023) conserved for them ranks of independent sister families. No suprageneric taxa were distinguished in Pararchaeidae by Rix (2006); malkarids were divided by Hormiga and Scharff (2020) to Sternoidinae, Tingotinginae, and Malkarinae.

1.1. Malkaridae (Mal).

**Figure 5.**
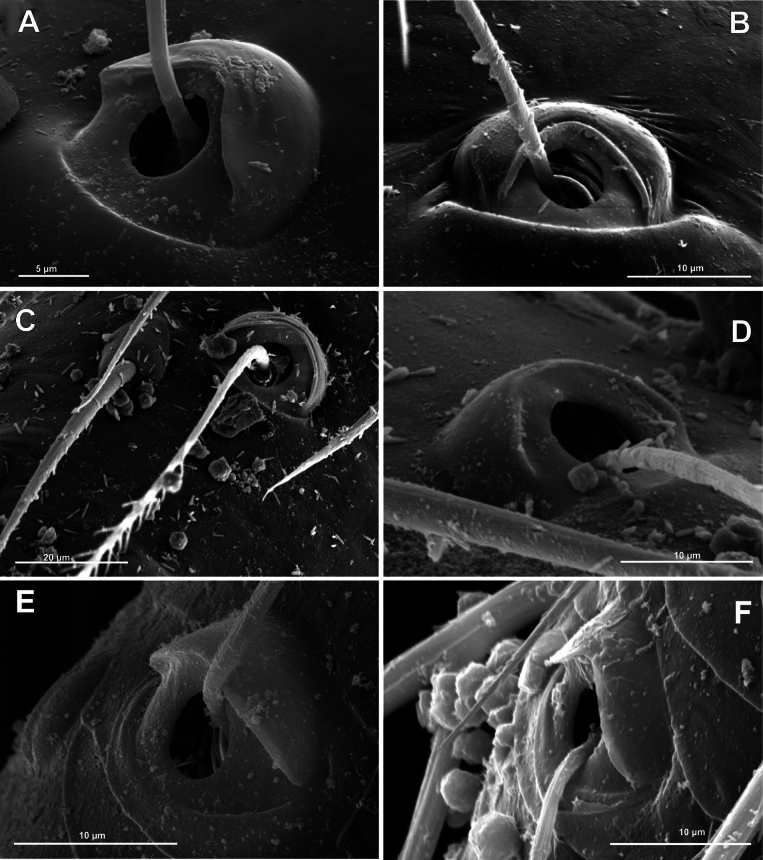
Bothria of ‘Malkaroid lineage’ of the *Erigone* type: Malkaridae (Malkarinae, Sternoidinae, Tingotinginae), Pararchaeidae**A***Chilenodesaustralis*, ti 3 (E-type) **B***Malkara* sp., ti 2 (E-type) **C***Tingotingo* sp., ti 4 (E-type) **D** the same **E***Flavarchaealulu*, ti 1 (E-type) **F***Anarchaeacorticola*, mt 3 (E-type).

The bothria of three genera representing all three malkarid subfamilies are studied here:

Malkarinae: *Malkara* Davies, 1980 (Fig. 5B);

Sternoidinae: *Chilenodes* Platnick & Forster, 1987 (Fig. 5A);

Tingotinginae: *Tingotingo* Hormiga & Scharff, 2020 (Fig. 5C, D).

In addition, the bothria of the three malkarid genera have been illustrated earlier: the sternoidin *Perissopmeros* Butler, 1932 (Moran 1986: fig. 17, as *Sternodes* Butler, 1932), *Malkara* (Davies 1980: fig. 9), and *Chilenodes* (Platnick and Forster 1987: fig. 18). The bothria of all three malkarid subfamilies belong to the ‘*Erigone*-type’ but differ in shape. A transverse ridge dividing the proximal bothrial plate (the ‘hood’) and the distal one is angled in sternodines (*ap*, Figs 3B, 5A; Moran 1986: fig. 17) and widely rounded in tingotingines (Fig. 5C, D); in the malkarines the ridge is angled, while the ‘hood’ itself is strongly swollen (Fig. 5B; Davies 1980: fig. 9), and its shape seems to be unique among araneoids. The setal shaft of *Tingotingo* (Fig. 5C) is very long and distinctly plumose.

1.2. Pararchaeidae (Par).

The bothria of two pararchaeid genera are studied here: *Anarchaea* Rix, 2006 and *Flavarchaea* Rix, 2006 (Fig. 5E, F). In addition, the bothrium of *Forstrarchaea* Rix, 2006 has been illustrated earlier by Forster and Platnick (1984: fig. 224, as *Pararchaea* Forster, 1955). The bothria of pararchaeids appear very uniform: the ‘hooded’ bothria of the ‘*Erigone*-type’, with a distinct angled transverse ridge. The diversity of the bothrial types in the ‘malkaroid lineage’ is summed in Fig. 28.

2. ‘Tetragnathoid lineage’, TE.

2.1. Arkyidae L.Koch, 1872 (Ark). Conventional subfamilies/tribes are not established [Arkyi].

2.2. Mimetidae Simon, 1881 (Mim).

2.2.1. Gelanorinae Mello-Leitão, 1935 [Gelan].

2.2.2. Mimetinae Simon, 1881 [Mimet].

2.2.3. Oarcinae Simon, 1890 [Oarci].

2.3. Tetragnathidae Menge, 1866 (Tet).

2.3.1. Diphyainae Simon, 1894 [Diphy].

2.3.2. Leucauginae Caporiacco, 1955 [Leuca].

2.3.3. Metainae Simon, 1894 [Metai].

2.3.4. Nanometinae Forster, 1999 [Nanom].

2.3.5. Tetragnathinae Menge, 1866 [Tetra].

‘Enlarged tetragnathoids’ were established by Dimitrov and Hormiga (2011) and Dimitrov et al. (2017) based on the molecular data; this lineage unites Tetragnathidae, Arkyidae, and Mimetidae, and is the sister group of ‘malkaroids’ (Hormiga and Scharff 2020: fig. 2). Arkyids were formerly considered an araneid subfamily, comprising two endemic Australian genera, *Arkys* Walckenaer, 1837 and *Demadiana* Strand, 1929 (Scharff and Coddington 1997; Framenau et al. 2010). Later they were re-ranked and relocated: “The arkyines (which we rank at the family level in our revised classification), represented here by nine terminals, are monophyletic and well supported but do not fall within Araneidae (where they are currently classified); instead, the arkyine clade is sister group to Tetragnathidae and this lineage is sister to Mimetidae” (Dimitrov et al. 2017: 229).

Mimetids (‘pirate spiders’ or ‘werewolf spiders’) are webless specialized araneophages, using the so-called ‘aggressive mimicry’. Currently, three subfamilies are recognized in mimetids: the globally distributed Mimetinae, the Neotropical Gelanorinae, and the endemic (southernmost South America) Oarcinae (Platnick and Shadab 1993). Recently, Benavides and Hormiga (2020), based on the molecular data and following Dimitrov et al. (2012), nested oarcins in araneids, but Eskov and Marusik (2023) refuted this relocation. In addition to the webless lifestyle and leg spination with the typically mimetid ‘capture basket’ (Platnick and Shadab 1993: figs 10, 11), the chelicerae of both oarcin genera, *Oarces* Simon, 1879 and *Gnolus* Simon, 1879, possess pore-bearing gland mounds and promarginal peg teeth replacing true teeth (Platnick and Shadab 1993: figs 16–18, 21–23), i.e., the key synapomorphies of Mimetidae never reported in Araneidae.

The orb-weaving tetragnathids were divided by Dimitrov and Hormiga (2011) into five subfamilies: Tetragnathinae, Leucauginae, Metainae, Diphyainae, and Nanometinae. Four tetragnathid genera of unclear subfamilial position (according to Álvarez-Padilla et al. 2009) are nested in this study according the cladogram given by Álvarez-Padilla and Hormiga (2011: fig. 144): *Allende* Álvarez-Padilla, 2007 and *Mollemeta* Álvarez-Padilla, 2007 in Tetragnathinae, *Chrysometa* Simon, 1894 in Diphyainae; as regards *Azilia* Keyserling, 1881, it is “either sister to Leucauginae with the morphology and behaviour data, or sister to all other tetragnathids with all data [mainly molecular] combined” (Álvarez-Padilla and Hormiga 2011: 728), and is listed here as ‘Tetragnathidae incertae sedis’. The East Asian genus *Guizygiella* Zhu, Kim & Song, 1997, placed in the monotypic subfamily Guizygiellinae by Zhu et al. (2003), was recently transferred by Kallal and Hormiga (2022) from Tetragnathidae to Araneidae.

2.1. Arkyidae (Ark).

**Figure 6.**
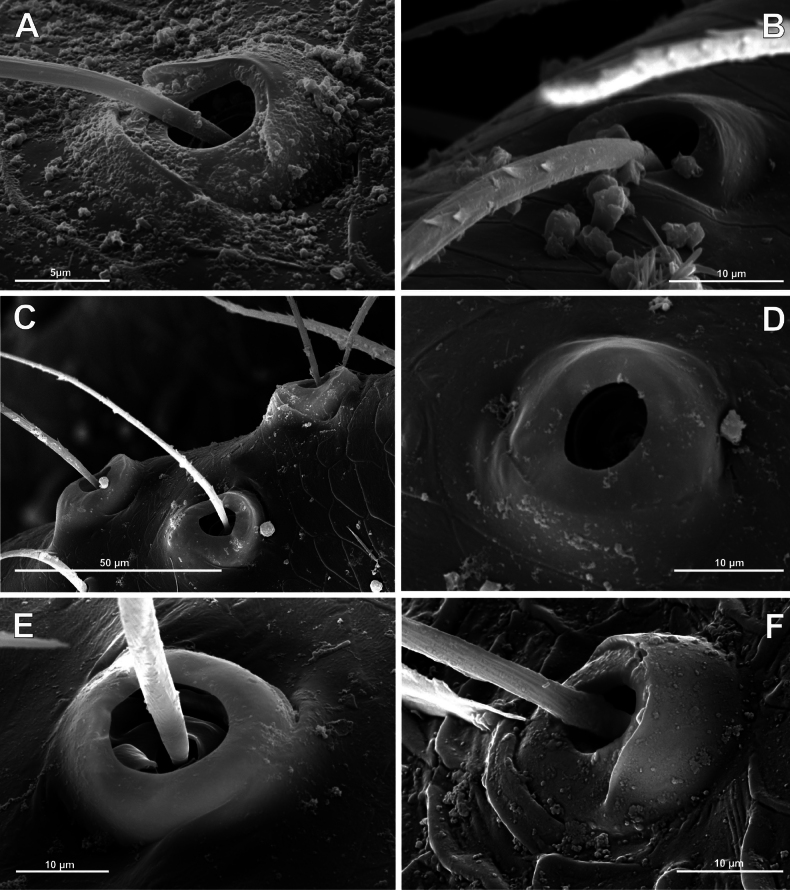
Bothria of ‘Tetragnathoid lineage’: Mimetidae (Oarcinae, Gelanorinae, Mimetinae), Arkyidae**A***Oarcesreticulatus*, ti 3 (E-type) **B***Gelanor* sp., ti 3 (E-type) **C***Erofurcata*, male palpal tibia (E-type) **D***Erofurcata*, ti 2, (A-type) **E***Australomimetustasmanensis*, ti 3 (T-type) **F***Arkysalticephala*, ti 2 (E-type).

The bothria of the single arkyid genus (of the couple comprising this family), *Arkys* Walckenaer, 1837, is studied here (Fig. 6F). It has the ‘hooded’ bothria of the ‘*Erigone*-type’ with an angled transverse ridge.

2.2. Mimetidae (Mim)

The bothria of four genera representing all three mimetid subfamilies are studied here:

Oarcinae: *Oarces* Simon, 1879 (Fig. 6A); Gelanorinae: *Gelanor* Thorell, 1869 (Fig. 6B); Mimetinae: *Ero* C. L. Koch, 1836 and *Australomimetus* Heimer, 1986 (Figs 4E, 6C–E). In addition, the bothria of the two mimetin genera have been illustrated earlier: *Mimetus* Hentz, 1832 (Griswold et al. 2005: fig. 149G) and *Australomimetus* (Forster and Platnick 1984: fig. 385). Platnick and Shadab (1993: fig. 13) have figured the bothrium of *Oarces*, and even mentioned its shape in the diagnosis of the subfamily: “[Oarcines] resemble mimetines in cheliceral gland mound and peg tooth morphology, as well as in having relatively smooth trichobothrial bases” (Platnick and Shadab 1993: 13); but this is clearly a misidentification: they dealt, in fact, with a setal socket instead of a bothrium. All three main bothrial types are present in Mimetidae. The bothria of Oarcinae and Gelanorinae both belong to the ‘*Erigone*-type’, although the bothrial transverse ridge is angled in oarcines (Fig. 6A) but rounded in the gelanorines (Fig. 6B). By contrast, the mimetin genera *Australomimetus* (Fig. 6E; Forster and Platnick 1984: fig. 385) and *Mimetus* (Griswold et al. 2005: fig. 149G) both possess dome-like bothria that belong to the ‘*Theridion*-type’. However, in the other mimetin genus, *Ero*, the composition of bothrial types seems to be unique. The bothria of its leg joints have a ridge smoothed over the entire length (Fig. 6D) and belong to the intermediate ‘*Argiope*-type’. By contrast, the bothria of a male papal tibia possess a clear rounded ridge (Fig. 6E), and thus should be attributed to the ‘*Erigone*-type’. The bothria of the male papal tibia sometimes differ from those of the leg joints (e.g., in physoglenid *Pahora* Forster, 1990: fig. 23A, B), but they never belong to two different types.

2.3. Tetragnathidae (Tet).

**Figure 7.**
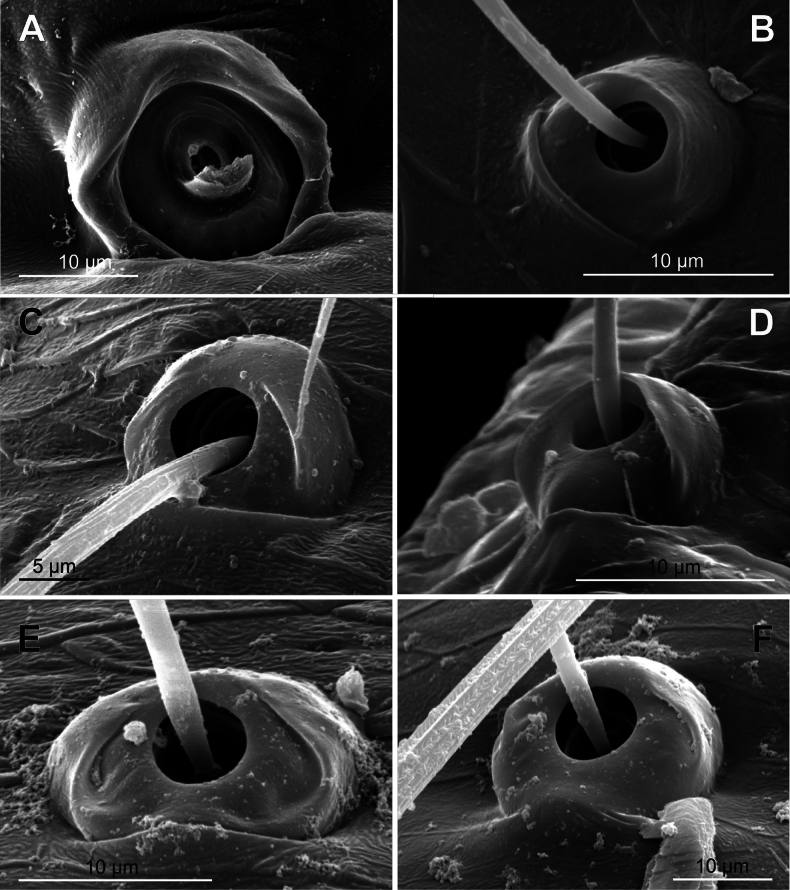
Bothria of ‘Tetragnathoid lineage’: Tetragnathidae (Diphyainae, Nanometinae) **A***Chrysometaalajuela*, ti 3 (E-type) **B**Diphyainae gen. sp., ti 4 (E-type) **C***Diphyawulingensis*, ti 3 (A-type) **D***Pinkfloydia* sp., ti 3 (E-type) **E***Nanometa* sp., ti 3 (A-type) **F***Orsinomesarasini*, ti 3 (A-type).

**Figure 8.**
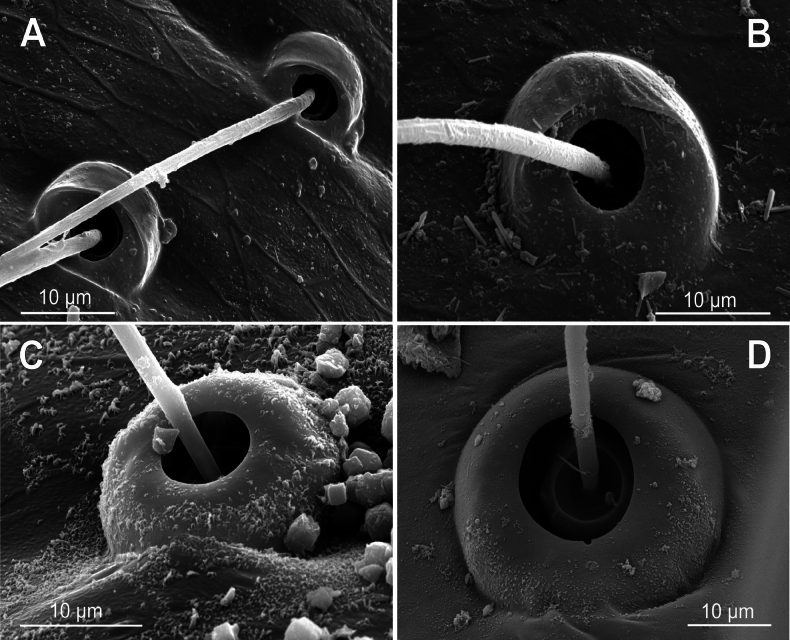
Bothria of ‘Tetragnathoid lineage’, Tetragnathidae (Leucauginae, Metainae) **A***Leucaugegranulata*, ti 3 (E-type) **B***Metleucaugedentipalpis*, ti 3 (A-type) **C***Metellinamengei*, ti1 (A-type) **D***Meta menardi*, ti 3 (T-type).

**Figure 9.**
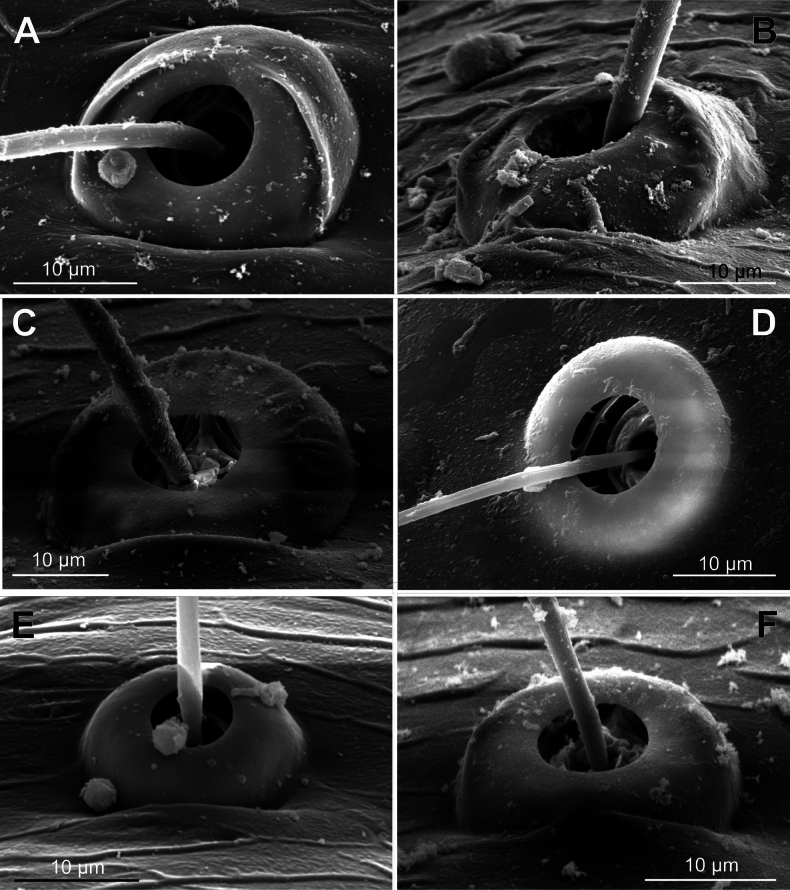
Bothria of ‘Tetragnathoid lineage’: Tetragnathidae (Tetragnathinae, Tetragnathidae incertae sedis) **A***Cyrtognathapachygnathoides*, ti 3 (E-type) **B***Allende* sp., ti 3 (A-type) **C***Mollemetaedwardsi*, ti 3 (A-type) **D***Pachygnathalisteri*, ti 2 (T-type) **E***Tetragnathaextensa*, ti 4 (T-type) **F***Azilia* sp., ti 2 (A-type).

The bothria of 16 genera representing all five tetragnathid subfamilies are studied here:

Diphyainae: *Chrysometa* Simon, 1894, *Diphya* Nicolet, 1849, and Diphyainae gen. sp. 1 (Fig. 7A–C); Leucauginae: *Leucauge* White, 1841 and *Metleucauge* Levi, 1980 (Fig. 8A, B); Metainae: *Meta* C.L. Koch, 1835 and *Metellina* Chamberlin & Ivie, 1941 (Fig. 8C, D); Nanometinae: *Nanometa* Simon, 1908, *Orsinome* Thorell, 1890 and *Pinkfloydia* Dimitrov & Hormiga, 2011 (Fig. 7D–F); Tetragnathinae: *Allende* Álvarez-Padilla, 2007, *Cyrtognatha* Keyserling, 1881, *Mollemeta* Álvarez-Padilla, 2007, *Pachygnatha* Sundevall, 1823 and *Tetragnatha* Latreille, 1804 (Fig. 9A–E); Tetragnathidae incertae sedis: *Azilia* Keyserling, 1881 (Fig. 9F).

No tetragnathid bothria have been illustrated in detail previously; numerous SEM images by Álvarez-Padilla and Hormiga (2011) reflect only the trichobothrial pattern of the legs, but not the bothrial morphology. All three bothrial types are represented in Tetragnathidae, and there are at least two types in every subfamily. Both the ‘*Erigone*-type’ and the more advanced ‘*Argiope*-type’ are combined in the three subfamilies: the Diphyainae (*Chrysometa*: Fig. 7A and Diphyainae gen. sp.1: Fig. 7B vs *Diphya*: Fig. 7C, respectively); in Nanometinae (*Pinkfloydia*: Fig. 7D vs *Nanometa*: Fig. 7E and *Orsinome*: Fig. 7F, respectively); and in Leucauginae (*Leucauge*: Fig. 8A vs *Metleucauge*: Fig. 8B, respectively). Both the intermediate ‘*Argiope*-type’ and the advanced ‘*Theridion*-type’ are combined in the subfamily Metainae (*Meta*: Fig. 8C vs *Metellina*: Fig. 8D, respectively). Finally, all three bothrial types are represented in Tetragnathinae: ‘*Erigone*-type’ in *Cyrtognatha* and *Allende* (Fig. 9A and Fig. 9B, respectively); ‘*Argiope*-type’ in *Mollemeta* (Fig. 9C); and ‘*Theridion*-type’ in *Pachygnatha* and *Tetragnatha* (Fig. 9D and Fig. 9E, respectively). The diversity of the bothrial types in the ‘Tetragnatoid lineage’ is summarized in Fig. 28.

‘**Araneoid branch’, ARAN**

3. ‘Araneoid lineage’, AR

3.1. Araneidae Clerck, 1757 (Ara).

3.1.1. Araneinae Clerck, 1757 [Arane].

3.1.2. Argiopinae Simon, 1890 [Argio].

3.1.3. Cyrtarachninae Simon, 1895 [Cyara].

3.1.4. Сyrtophorinae Simon, 1895 [Cypho].

3.1.5. Gasteracanthinae O. Pickard-Cambridge, 1871 [Gaste].

3.1.6. Micratheninae Simon, 1895 [Mithe].

3.1.7. Guizygiellinae Zhu, Song & Zhang, 2003 [Guizy].

3.1.8. Caerostrini Simon, 1895 [Caero].

3.1.9. Hypognathini Simon, 1895 [Hypog].

3.1.10. Poltyini Simon, 1895 [Polty].

3. 1. 11. Testudinarini Simon, 1895 [Testu].

3.2. Nephilidae Simon, 1894 (Nep). Conventional subfamilies/tribes are not established [Nephi].

3.3. Zygiellidae Simon, 1929 (Zyg). Conventional subfamilies/tribes are not established [Zygie].

Araneidae formerly were usually considered (i.e., before the recognition of ‘enlarged Tetragnathoids’ by Dimitrov et al. [2017]) as a sister group of the other Araneoidea (e.g., Scharff and Coddington 1997; Griswold et al. 1998). In the classification of araneids accepted in this paper, we follow Scharff and Coddington (1997), who recognized six subfamilies(Araneinae, Argiopinae, Cyrtarachninae, Сyrtophorinae, Gasteracanthinae, and Micratheninae), mainly corresponding to the araneid subfamilies recognized by Simon (1895); in addition, the cyrtarachnin tribe Mastophorini Mello-Leitão, 1931 (the ‘bolas spiders,’ possessing a unique foraging behavior) is sometimes elevated to the subfamilial rank (see Scharff and Hormiga 2012). Subfamilial assignment of several araneid genera (*Caerostris* Thorell, 1868, *Hypognatha* Guérin, 1839, *Poltys* C.L. Koch, 1843, and *Melychiopharis* Simon, 1895) is still uncertain, and herein they are listed, conditionally, in the ’old’ Simon`s (1895) tribes (Caerostrini, Hypognathini, Poltyini, and Testudinarini, respectively). Kallal and Hormiga (2022) transferred the genus *Guizygiella* from Tetragnathidae to Araneidae, but did not indicate its subfamilial status; so, we are listing this genus here in the subfamily Guizygiellinae, which was not discussed by Kallal and Hormiga (2022). Additionally, several new suprageneric clades were recently distinguished in the family cladogram by molecular methods, but the authors frankly pointed out that “few of these groups are currently corroborated by morphology, behaviour, natural history or biogeography” (Scharff et al. 2020: 1) so we have also refrained from recognizing these ‘virtual clades’. Kuntner et al. (2023) established the new family Paraplectanoididae Kuntner, Coddington, Agnarsson & Bond, 2023 for the monotypic araneid genus *Paraplectanoides* Keyserling, 1886; however, Hormiga et al. (2023) returned it to Araneidae. Nephilids (‘golden orbweavers’) were for a long time considered a sister group of Tetragnathidae or even included in tetragnathids as a subfamily (e.g., Levi and Eickstedt 1989; Zhu et al. 2003), but recently they were clearly nested into the ‘Areneoid lineage’ (Álvarez-Padilla et al. 2009; Dimitrov et al. 2017; Kuntner et al. 2019). Zygiellids have been frequently relocated from araneids to tetragnathids and vice versa (see review in Gregorič et al. 2015), and finally were also nested in the ‘Araneoid lineage’. Kuntner et al. (2019) transferred the Australian genus *Phonognatha* Simon, 1894 to Zygiellidae (based on molecular data) and resurrected the old name Phonognatheae Simon, 1894 (as Phonognathidae) for this newly established clade. This name was not accepted by Scharff et al. (2020) and Eskov and Marusik (2023) but confirmed by Kallal et al. (2020) and Kuntner et al. (2023).

However, the diagnosis of Phonognathidae provided by Kuntner et al. (2023: 969) contains a single morphological character: “…Distal grouping of setae on palpal tibia and elongated male palpal femur”, and this character does not seem convincing. So, Phonognathidae sensu Kuntner et al. (2019) remains a purely molecular clade. Until the assignment of both type genera, *Phonognatha* and *Zygiella* F. O. Pickard-Cambridge, 1902, to a united clade is supported by morphological synapomorphies, we prefer to use the established name Zygiellidae for this taxon. Kuntner et al. (2019: 557, 563) listed the genera attributed by them to Nephilidae and Phonognathidae but have not divided these taxa to subfamilies or tribes. Hormiga et al. (2023) reranked these families as subfamilies of Araneidae but also did not assign them to the subtaxa.

3.1. Araneidae (Ara).

**Figure 10.**
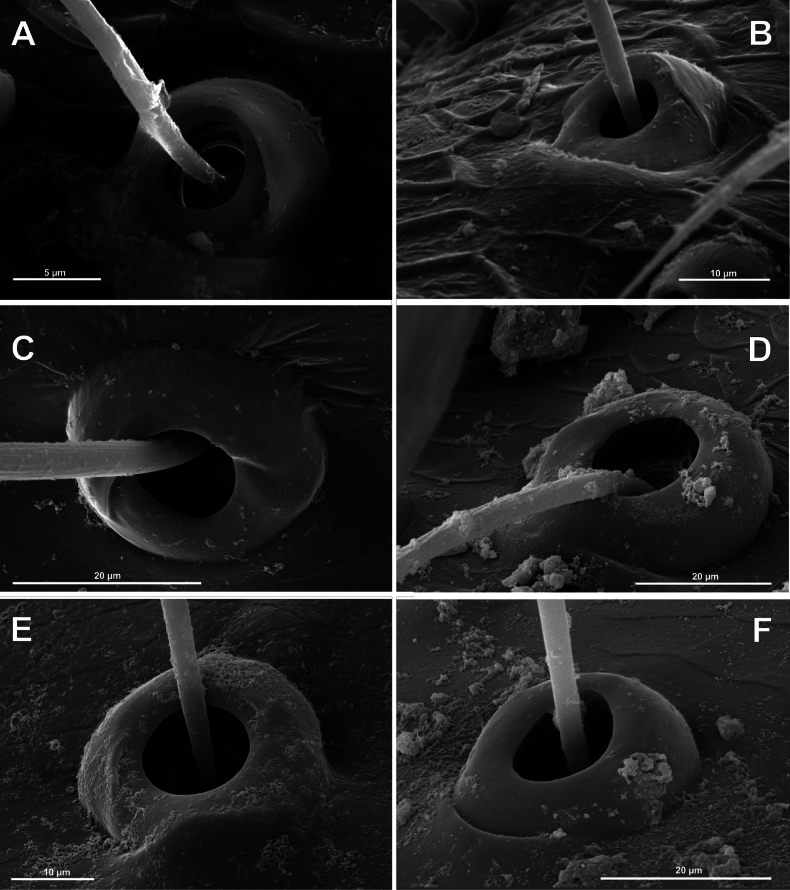
Bothria of ‘Araneoid lineage’: Araneidae (Cyrtarachninae, Argiopinae, Cyrtophorinae, Micratheninae, Gasteracanthinae) **A***Cyrtarachneixoides*, ti 3 (E-type) **B***Chorizopes* sp., ti 3 (E-type) **C***Argiopebruennichi*, ti 3 (A-type) **D***Cyrtophoramoluccensis*, ti 3 (A-type) **E***Micrathena* sp., ti 3 (A-type) **F***Gasteracanthadiadesmia*, ti 3 (T-type).

**Figure 11.**
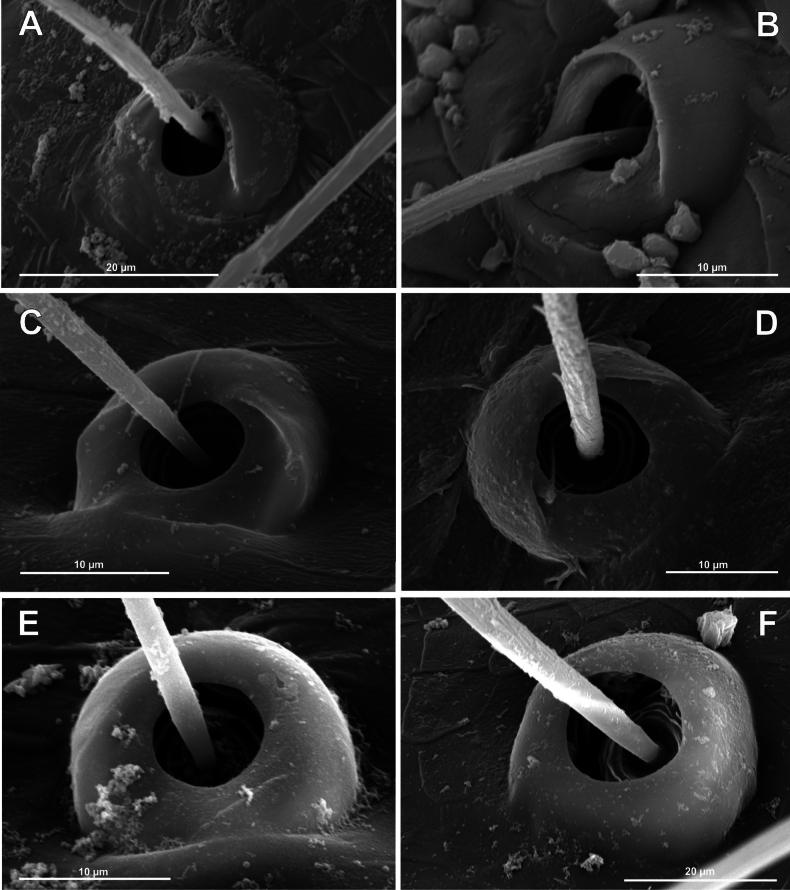
Bothria of ‘Araneoid lineage’, Araneidae (Araneinae) **A***Singahamata*, ti 3 (E-type) **B***Hypsosingapygmaea*, ti 3 (E-type) **C***Lariniabonneti*, ti 3 (A-type) **D***Mangoraacalypha*, ti 3 (A-type) **E***Cyclosaconica*, ti 3 (T-type) **F***Araneusdiadematus*, ti 3 (T-type).

**Figure 12.**
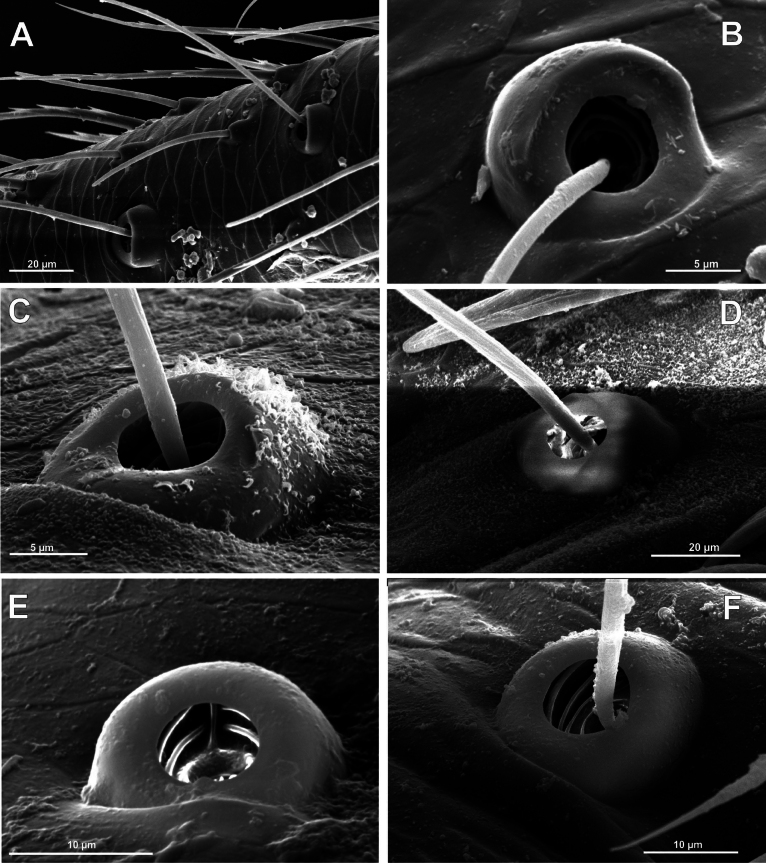
Bothria of ‘Araneoid lineage’: Araneidae (Testudinareae, Guizygiellinae, Caerostreae, Poltyeae, Hypognatheae) **A***Melychiopharis* sp., mt 3 (E-type) **B***Melychiopharis* sp., ti 3 (E-type) **C***Guizygiella* sp., ti 1 (A-type) **D***Caerostrissumatrana*, mt 4 (A-type) **E***Poltys* sp., ti 2 (T-type) **F***Hypognatha* sp., ti 3 (T-type).

The bothria of 17 genera representing all six conventional araneid subfamilies and the four tribes of uncertain position are studied here: Araneinae: *Araneus* Clerck, 1757, *Cyclosa* Menge, 1866, *Hypsosinga* Ausserer, 1871, *Larinia* Simon, 1874, *Mangora* O. Pickard-Cambridge, 1889, and *Singa* C.L. Koch, 1836 (Fig. 11A–F); Argiopinae: *Argiope* Audouin, 1826 (Fig. 10C); Cyrtarachninae: *Chorizopes* O. Pickard-Cambridge, 1871 and *Cyrtarachne* Thorell, 1868 (Fig. 10A, B); Cyrtophorinae: *Cyrtophora* Simon, 1864 (Fig. 10D); Gasteracanthinae: *Gasteracantha* Sundevall, 1833 (Fig. 10F); Micratheninae: *Micrathena* Sundevall, 1833 (Fig. 10E); Caerostrini: *Caerostris* Thorell, 1868 (Fig. 12D); Hypognathini: *Hypognatha* Guérin, 1839 (Fig. 12F); Poltyini: *Poltys* C.L. Koch, 1843 (Fig. 12E); Testudinarini: *Melychiopharis* Simon, 1895 (Fig. 12A, B); Araneidae incertae sedis: Guizygiellinae: *Guizygiella* Zhu, Kim & Song, 1997 (Fig. 12C).

The bothria of only two araneid genera have been illustrated earlier: *Metepeira* F.O. Pickard-Cambridge, 1903 (Griswold et al. 1998: fig. 22E) and *Novaranea* Court & Forster, 1988 (Court and Forster 1988: fig. 552). Despite the scarcity of the available data, Court and Forster (1988: 70) provided a generalization: “The bothria arc [in araneids] smooth with the posterior hood reduced so that only lateral ridges are visible”, which seems like an adequate description of the ‘*Argiope*-type’ of bothria. In fact, however, the intermediate ‘*Argiope*-type’ of the bothria is widespread among the araneid subtaxa, but the other bothrial types, both the ancestral ‘*Erigone*-type’ and the advanced ‘*Theridion*-type’, are also well represented in this family.

The ‘*Argiope*-type’ is the character of Argiopinae, Cyrtophorinae, Micratheninae, and Caerostrini (Fig. 10C–E and Fig. 12D, respectively). The ‘*Erigone*-type’ is a character of Cyrtarachninae and Testudinarini (Fig. 10A, B and Fig. 12A, B, respectively), whereas the ‘*Theridion*-type’ is a character of Gasteracanthinae, the Hypognathini, and the Poltyini (Figs 10F, 12F, E, respectively).

Finally, all three bothrial types are represented in Araneinae: the ‘*Erigone*-type’ in *Singa* and *Hypsosinga* (Fig. 11A, B, respectively); the ‘*Argiope*-type’ in *Larinia* and *Mangora* (Fig. 11C, D, respectively); and the ‘*Theridion*-type’ in *Cyclosa* and *Araneus* (Fig. 11E, F, respectively).

It should be mentioned that the transverse ridge of the ‘*Erigone*-type’ bothria in araneids can be either angled or rounded; however, no regularities are traceable in this respect. In cyrtarachnines the ridge is rounded in *Cyrtarachne* and angled in *Chorizopes* (Fig. 10A, B, respectively); in the araneines the ridge is angled in *Singa* and rounded in *Hypsosinga* (Fig. 11A, B, respectively); in testudinarine *Melychiopharis* the ridge is rounded (Fig. 12B).

3.2. Nephilidae (Nep).

**Figure 13.**
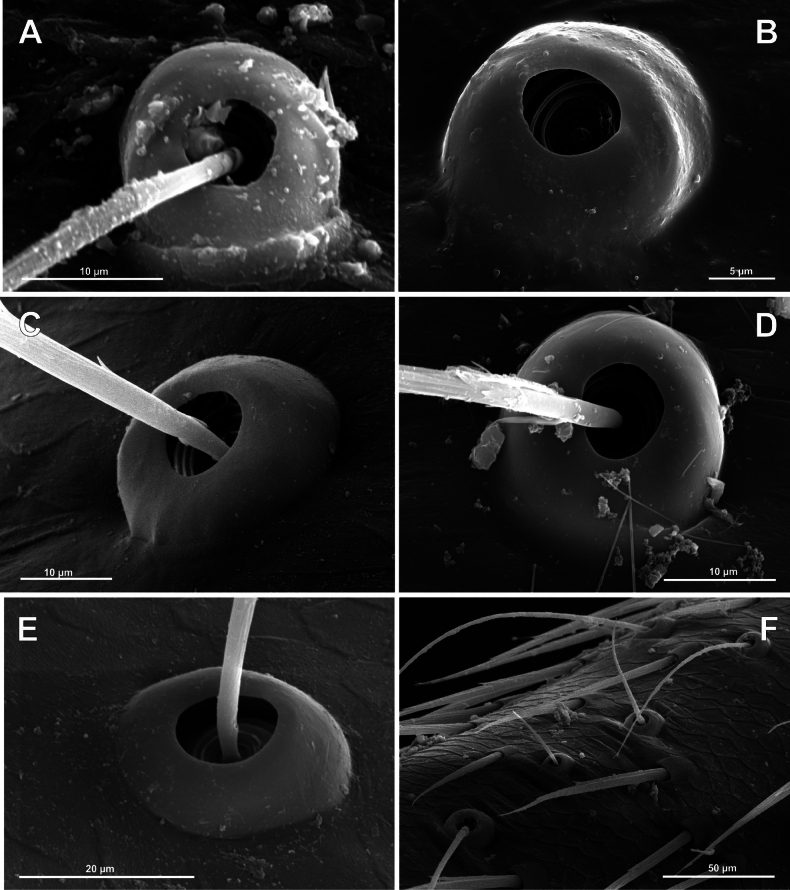
Bothria of ‘Araneoid lineage’: Zygiellidae, Nephilidae**A***Zygiellax-notata*, ti 3 (A-type) **B***Levielluscaspicus*, ti 3 (A-type) **C***Parazygielladispar*, ti 3 (T-type) **D** ‘Zygiella’ atrica, ti 3 (T-type) **E***Nephila* sp., ti 3 (T-type) **F***Nephilengysmalabarensis*, ti 3 (T-type).

The bothria of two nephilid genera are studied here: *Nephila* Leach, 1815 and *Nephilengys* L. Koch, 1872 (Fig. 13E, F). The bothria of both genera are the uniformly dome-like bothria and belong to the ‘*Theridion*-type’. It should be noted that the bothria of *Nephila* strongly vary in size (Fig. 13F).

3.3. Zygiellidae (Zyg).

The bothria of three zygiellid genera are studied here: *Leviellus* Wunderlich, 2004, *Zygiella* F. O. Pickard-Cambridge, 1902, and *Parazygiella* Wunderlich, 2004. The bothria of the ‘*Argiope*-type’ and the ‘*Theridion*-type’ are represented in Zygiellidae (*Leviellus*: Fig. 13B vs *Parazygiella*: Fig. 13C). Moreover, both types are recorded in the single genus *Zygiella*: the ‘*Argiope*-type’ in *Z.x-notata* (Clerck, 1757) (Fig. 13A) vs the ‘*Theridion*-type’ in *Z.atrica* (C.L. Koch, 1845) (Fig. 13D). The latter case could be stated as unique, but there is serious surmise that the above two *Zygiella* species are not congeneric (see below, in Discussion). The diversity of the bothrial types in the ‘Araneoid lineage’ is summarized in Fig. 29.

4. ‘Symphytognathoid lineage’, SY

4.1. Anapidae Simon, 1895 (Ana).

4.1.1. Anapinae Simon, 1895 [Anapi].

4.1.2. Gigiellinae Rix & Harvey, 2010 [Gigie].

4.1.3. Holarchaeinae Forster & Platnick, 1984 [Holar].

4.1.4. Taphiassinae Rix & Harvey, 2010 [Taphi].

4.1.5. Teutoniellinae Rix & Harvey, 2010 (stat. nov.) [Teuto].

4.2. Comaromidae Wunderlich, 2004 (Com). Conventional subfamilies/tribes are not established [Comar].

4.3. Micropholcommatidae Hickman, 1944 (Mic).

4.3.1. Micropholcommatinae Hickman, 1944 [Micph].

4.3.2. Textricellinae Hickman, 1945 [Textr].

4.4. Mysmenidae Petrunkevitch, 1928 (Mys).

4.4.1. Mysmeninae Petrunkevitch, 1928 [Mysme].

4.4.2. Mysmenopsinae Lopardo & Hormiga, 2015 [Mysps].

4.5. Symphytognathidae Hickman,1931 (Sym). Conventional subfamilies/tribes are not established [Symph].

4.6. Synaphridae Wunderlich, 1986 (Syn). Conventional subfamilies/tribes are not established [Synap].

4.7. Theridiosomatidae Simon, 1881 (Ths).

4.7.1. Epeirotypinae Archer, 1953 [Eptyp].

4.7.2. Ogulninae Coddington, 1986 [Oguln].

4.7.3. Platoninae Coddington, 1986 [Plato].

4.7.4. Theridiosomatinae Simon, 1881 [Thsom].

Forster (1959) united the minute, mainly apneumonic spiders of the families Symphytognathidae, Anapidae, Textricellidae, Micropholcommatidae, and Mysmenidae in his Symphytognathidae s. l. Later Forster and Platnick (1984) attempted to relocate micropholcommatids with textricellids from Araneoidea to their ‘enlarged Palpimanoidea’, but this hypothesis was refuted by Schütt (2000, 2003) and subsequent authors. Coddington (1986a, b) suggested that Anapidae, Mysmenidae, and Symphytognathidae form a monophyletic taxon and comprise the sister group of Theridiosomatidae. A cladogram of Symphytognathoidea was presented by Lopardo et al. (2011) as Theridiosomatidae (Mysmenidae (Synaphridae (Symphytognathidae (Anapidae s. l. (Anapinae + Micropholcommatinae))))).

However, recently Dimitrov et al. (2017: fig. 2), declared the polyphyly of Symphytognathoidea based on molecular data and distributed symphytognathoid families over various, very distant, araneoid clades: mysmenids appeared coupled with tetragnathoids, theridiosomatids with synotaxids, anapids were divided into ‘Anapidae I’ and ‘Anapidae II’ (sic!) and coupled with theridiids and cyatholipids, respectively. However, these ‘new molecular clades’ are lacking not only morphological, but also a sufficient molecular support: “The symphytognathoid families constitute a polyphyletic group, although all nodes involving these interfamilial relationships receive low support values” (Dimitrov et al. 2017: 228). For these reasons, Eskov and Marusik (2023) restored Symphytognathoidea back to its traditional status as a monophyletic taxon.

The ‘Gondwanan’ micropholcommatids are regarded in this study, following Rix and Harvey (2010), as a sister group to anapids, but in a separate family, comprising the subfamilies Micropholcommatinae and Textricellinae (Eskov and Marusik 2023). Anapidae s. str. comprises subfamilies Taphiassinae and Gigiellinae (both transferred from micropholcommatids), Holarchaeinae and Anapinae (the rest of the anapid genera, including the basal monotypic *Acrobleps* Hickman, 1979) (Eskov and Marusik 2023). For the ‘teutoniellid taxa clade’ or ‘teutoniellids’, designated by Rix and Harvey (2010) and considered a sister group of their micropholcommatids (Rix and Harvey 2010: figs 3, 4), it turned out that it possesses a complete set of the diagnostic characters of Anapidae s. str., including the supramaxillar pore-bearing depressions, and should be treated as Anapidae: Teutoniellinae (stat. nov.).

The key diagnostic characters (i.e., unambiguous synapomorphies found in all clade members without exception and never outside it) of the Anapidae s. l. (Anapidae s. str. + Micropholcommatidae) main taxa are: (1) the cheliceral gland mound fussed with a proximal promarginal tooth, and a particular ‘key-lock’ mode of fixation of the bulb in a naturally expanded condition (Anapidae s. l.); (2) the cheliceral promargin with peg teeth replacing true teeth, and a pair of fused setal sockets adjacent to the fang base (Micropholcommatidae); and (3) a pair of pore-bearing carapace depressions strictly above the maxilla (Anapidae s. str.) (pers. obs.).

The family Holarchaeidae was established by Forster and Platnick (1984) to accommodate a single genus *Holarchaea* Forster, 1955 from New Zealand and Tasmania. Its taxonomic position was still enigmatic until Dimitrov et al. (2017) found that *Holarchaea* is close to, according to molecular data, the anapid genus *Acrobleps*, and on this ground synonymized Holarchaeidae with Anapidae. The study of *Holarchaea* microstructures (pers. obs.) confirms that it is indeed nothing more than an aberrant anapid: it possesses the complete set of anapid diagnostic characters, including vestiges of the pore-bearing carapace supramaxillary depression, and was treated as the anapid subfamily Holarchaeinae (Eskov and Marusik 2023).

The family Comaromidae was established by Wunderlich (2004b) as the anapid subfamily Comarominae to comprise the controversial genus *Comaroma* Bertkau, 1889, nested in Anapidae by Kropf (1990), and later elevated to family rank (Wunderlich 2011). The status of Comaromidae Wunderlich, 2004 as a separate symphytognathoid family was seconded recently by Eskov and Marusik (2022, 2023). *Comaroma* appeared to lack both unique synapomorphies of Anapidae, i.e., the cheliceral gland-mound fused with the proximal promarginal tooth and the pore-bearing depressions at the edge of the carapace, while retaining the lateral paracymbium, the male epiandrous spigots, and the suprapedicellar setae, lost in all members of symphytognathoidan ‘EbCY clade’ (Anapidae + Symphytognathidae).

The position of Synaphridae in araneoids was controversial and ping-ponged from family to family. This taxon was established by Wunderlich (1986) as a subfamily of anapids, then elevated to family rank and restricted to two genera, *Synaphris* Simon, 1894 and *Cepheia* Simon, 1894, by Marusik and Lehtinen (2003). It was nested in Symphytognathoidea (Schütt 2003), rejected from this clade by Marusik and Lehtinen (2003) and Lopardo et al. (2007) (who placed it together with Theridiidae and Cyatholipidae, respectively), and finally returned to symphytognathoids (Lopardo et al. 2011; Lopardo and Hormiga 2015).

Regarding the remaining symphytognathoid families, they are accepted herein within traditional scopes and limits. Coddington (1986b) has divided theridiosomatids into four subfamilies (Platoninae, Epeirotypinae, Ogulninae, and Theridiosomatinae); Lopardo and Hormiga (2015) distinguished two subfamilies in mysmenids (Mysmeninae and Mysmenopsinae), leaving several genera unclassified; Symphytognathidae (as well as the above Synaphridae), are still undivided to conventional subfamilies or tribes.

4.1. Anapidae (Ana).

**Figure 14.**
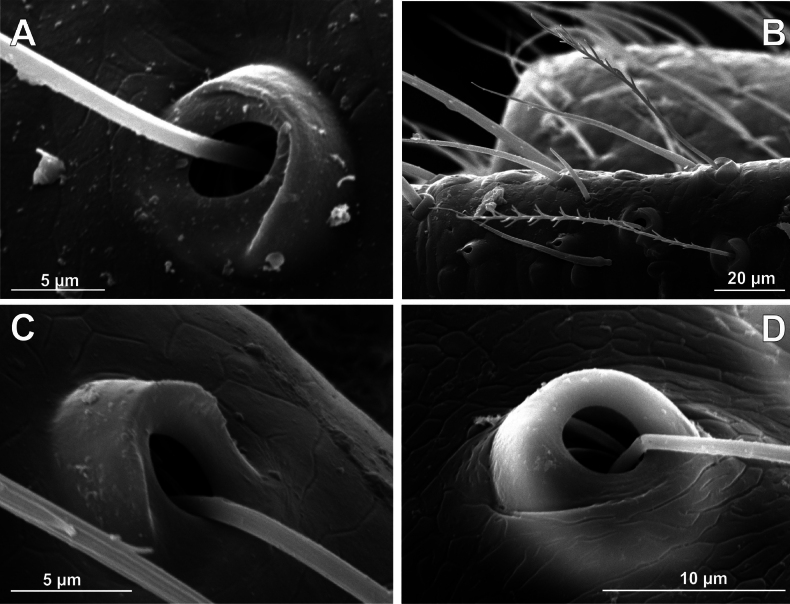
Bothria of ‘Symphytognathoid lineage’: Anapidae (Anapinae) **A***Acroblepshygrophilus*, ti 3 (E-type) **B***Minanapis* sp., ti 2 (E-type) **C***Montanapis* sp., ti 3 (E-type) **D***Pseudanapis* sp., ti 2 (A-type).

**Figure 15.**
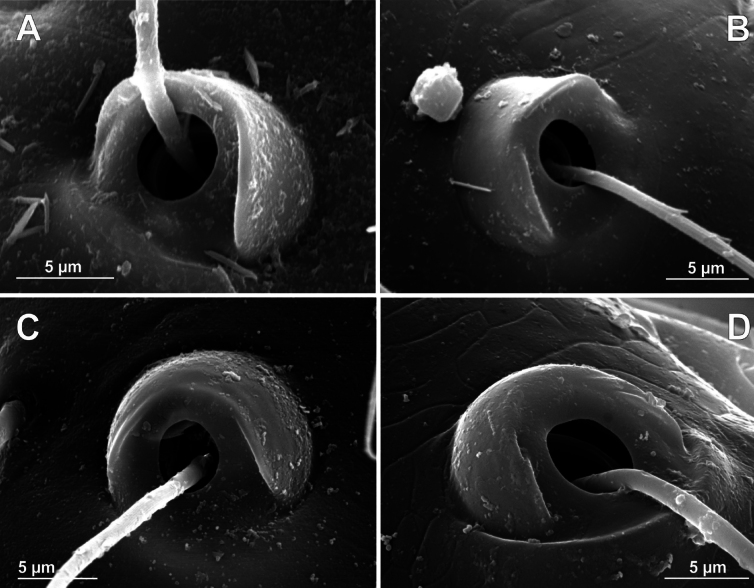
Bothria of ‘Symphytognathoid lineage’: Anapidae (Holarchaeinae, Teutoniellinae, Gigiellinae, Taphiassinae) **A***Holarchaeanovaeseelandiae*, ti 3 (E-type) **B***Teutoniellacekalovici*, ti 4 (E-type) **C***Gigiellamillidgei*, mt 2 (E-type) **D***Taphiassacastanea*, ti 2 (A-type).

The bothria of 14 genera representing all five anapid subfamilies are studied here:

Anapinae: *Acrobleps* Hickman, 1979; *Minanapis* Platnick & Forster, 1989; *Montanapis* Platnick & Forster, 1989; and *Pseudanapis* Simon, 1905 (Fig. 14A–D); as well as *Crassanapis* Platnick & Forster, 1989; *Elanapis* Platnick & Forster, 1989; *Hickmanapis* Platnick & Forster, 1989; *Sofanapis* Platnick & Forster, 1989; *Zangherella* Caporiacco, 1949; and *Zealanapis* Platnick & Forster, 1989 (images are not presented herein); Gigiellinae: *Gigiella* Rix & Harvey, 2010 (Fig. 15C); Holarchaeinae: *Holarchaea* Forster, 1955 (Fig. 15A); Taphiassinae: *Taphiassa* Simon, 1880 (Fig. 15D); Teutoniellinae: *Teutoniella* Brignoli, 1981 (Fig. 15B).

In addition, the bothria of seven anapid genera have been figured earlier: *Crassanapis*, *Minanapis* and *Risdonius* Hickman, 1939 (by Platnick and Forster 1989: figs 15, 81, and 225, respectively); *Holarchaea* (Forster and Platnick 1984: fig. 247); *Taphiassa* (Rix and Harvey 2010: figs 160C, D, 173C); *Olgania* Hickman, 1979 (Rix and Harvey 2010: fig. 191); and *Gigiella* (Rix and Harvey 2010: figs 200C, D, 207C, D). The ancestral ‘*Erigone*-type’ of bothria dominates in Anapidae; the bothria of the intermediate ‘*Argiope*-type’ are rare, and the advanced ‘*Theridion*-type’ is completely absent. Only the ‘*Erigone*-type’ present in the monogeneric subfamilies Gigiellinae (Fig. 15C; Rix and Harvey 2010: figs 200C, D, 207C, D), Holarchaeinae (Fig. 15A; Forster and Platnick 1984: fig. 247), and Teutoniellinae (Fig. 15B).

An unusual case is observed in the subfamily Anapinae. Among its 15 studied members, 14 have bothria of the ‘*Erigone*-type’ with the angled ridge (e.g., *Acrobleps*, *Minanapis*, and *Montanapis*: Fig. 14A–C, respectively). However, a single exception was recorded: the otherwise unremarkable advanced genus *Pseudanapis* has the ‘*Argiope*-type’ of bothria (Fig. 14D). Coexistence of the two bothrial types in a single taxon is not unusual, e.g., Mimetinae (see above), Taphiassinae, and Micropholcommatinae (see below), but the Anapinae would have been considered ‘uniform’ with respect to bothrial type, if not for the case of *Pseudanapis*, which is the “pebble in a shoe”. The second case of ‘*Argiope*-type’ bothria in anapids is in the subfamily Taphiassinae (Fig. 15D). This bothrial type was recorded in both studied *Taphiassa* species, *T.robertsi* Rix & Harvey, 2010 and *T.castanea* Rix & Harvey, 2010 (Rix and Harvey 2010: fig. 160C, D and fig. 173C, respectively). However, the second taphiassin genus, *Olgania*, has the usual anapid bothria of the ‘*Erigone*-type’ (Rix and Harvey 2010: fig. 191C).

4.2. Comaromidae (Com).

The bothria of *Comaroma* Bertkau, 1889 are studied here (Fig. 18B). In addition, the bothrium of *Comaroma* has been illustrated earlier (Lopardo and Hormiga 2015: fig. 80C). The bothria belong to the ‘*Erigone*-type’ with a sharp, orthogonally angled ridge.

4.3. Micropholcommatidae (Mic).

**Figure 16.**
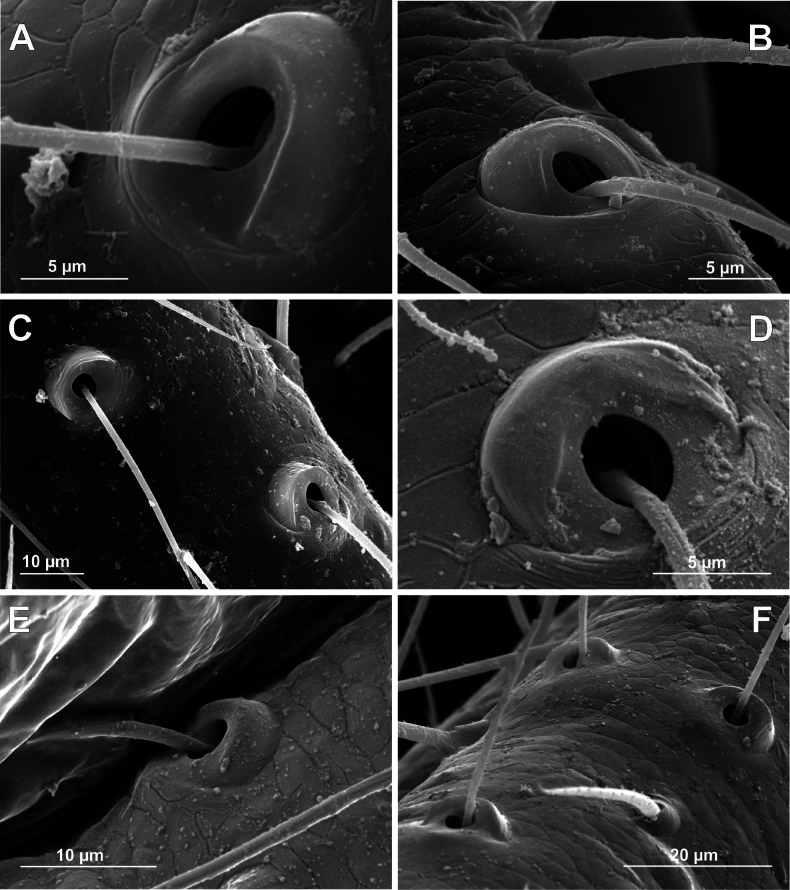
Bothria of ‘Symphytognathoid lineage’: Micropholcommatidae (Micropholcommatinae, Textricellinae) **A***Micropholcommaparmata*, ti 4 (E-type) **B***Micropholcommabryophilum*, ti 3 (E-type) **C***Tricellinagertschi*, ti 2 (E-type) **D***Plectochetoslongissimus*, ti 4 (A-type) **E***Austropholcomma* sp., ti 3 (A-type) **F***Rayforstiavulgaris*, ti 1 (E-type).

The bothria of ten genera representing both micropholcommatid subfamilies distinguished herein have been examined: Micropholcommatinae: *Austropholcomma* Rix & Harvey, 2010; *Micropholcomma* Crosby & Bishop, 1927; *Plectochetos* Butler, 1932; *Tricellina* Forster & Platnick, 1989 (Fig. 16A–E); Textricellinae: *Rayforstia* Rix & Harvey, 2010 (Fig. 16F); *Eperiella* Rix & Harvey, 2010; *Epigastrina* Rix & Harvey, 2010; *Eterosonycha* Butler, 1932; *Normplatnicka* Rix & Harvey, 2010; *Raveniella* Rix & Harvey, 2010 (images are not presented herein). In addition, the bothria of the nine micropholcommatid genera were figured earlier: Micropholcommatinae: *Plectochetos* (Forster and Platnick 1984: fig. 375, as *Micropholcomma*); *Micropholcomma*; *Pua* Forster, 1959; *Austropholcomma*; *Tricellina*; and *Patelliella* Rix & Harvey, 2010 (Rix and Harvey 2010: figs 20C, 28C, 38C, 44C, and 149C, respectively); Textricellinae: *Rayforstia* (Forster and Platnick 1984: fig. 374, as *Textricella*); *Algidiella* Rix & Harvey, 2010; *Taliniella* Rix & Harvey, 2010; and *Tinytrella* Rix & Harvey, 2010 (Rix and Harvey 2010: figs 124C, 133C, and 141C, respectively).

All the genera of Textricellinae have a highly uniform bothria of the ‘*Erigone*-type’ with an angled ridge (e.g., Fig. 16F; Rix and Harvey 2010: figs 124C, 133C, 141C). In contrast, both the ancestral ‘*Erigone*-type’ and the more advanced ‘*Argiope*-type’ are present in the Micropholcommatinae, and, in addition, the transverse ridge is rounded rather than angled. The genera with the clear ‘*Erigone*-type’ bothria are *Tricellina* (Fig. 16C; Rix and Harvey 2010: fig. 44C) and *Pua* (Rix and Harvey 2010: fig. 28C). The genera with the clear ‘*Argiope*-type’ bothria are *Austropholcomma* (Fig. 16E; Rix and Harvey 2010: fig. 38C), *Plectochetos* (Fig. 16D; Forster and Platnick 1984: fig. 375), and *Patelliella* (Rix and Harvey 2010: fig. 149C). Finally, in the species of *Micropholcomma* the bothria seem to be of an intermediate between the ‘*Erigone*-type’ and the ‘*Argiope*-type’; moreover, even within this genus *M.bryophilum* (Butler, 1932) (Fig. 16B; Rix and Harvey 2010: fig. 20C) seems to be closer to the ‘*Argiope*-type’ than *M.parmatum* Hickman, 1944 (Fig. 16A).

4.4. Mysmenidae (Mys).

**Figure 17.**
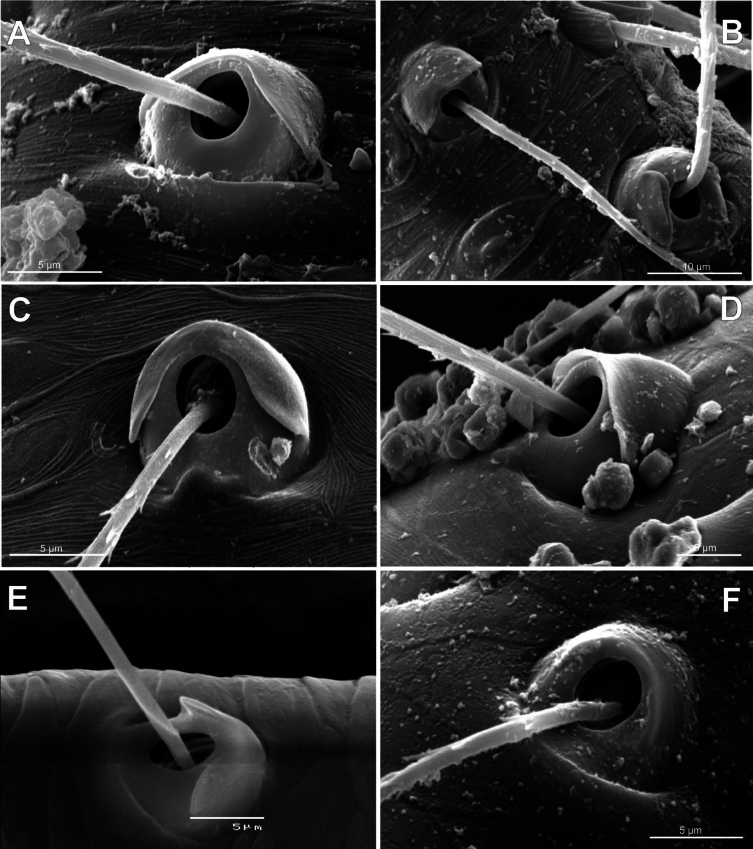
Bothria of ‘Symphytognathoid lineage’: Mysmenidae (Mysmeninae, Mysmenopsinae), Synaphridae**A***Mysmenaleucoplagiata*, ti 3 (E-type) **B***Microdipoena* sp., ti 1 (E-type) **C***Mysmenopsistengellacompa*, ti 2 (E-type) **D***Iselainquilina*, ti 2 (E-type) **E***Synaphrislehtineni*, ti 2 (E-type) **F***Cepheialongiseta*, ti 2 (A-type).

The bothria of four genera representing both conventional mysmenid subfamilies are studied here: Mysmeninae: *Mysmena* Simon, 1894 and *Microdipoena* Banks, 1895 (Fig. 17A, B); Mysmenopsinae: *Mysmenopsis* Simon, 1898 and *Isela* Griswold, 1985 (Fig. 17C, D). In addition, the bothria of the three mysmenid genera were figured previously: *Microdipoena* and *Mysmena* (Lopardo and Hormiga 2015: fig. 21I and fig. 34B, respectively), and *Isela* (Griswold 1985: fig. 8). The bothria of all mysmenids are highly uniform and belong to the ‘*Erigone*-type’ with an angled ridge.

**Figure 18.**
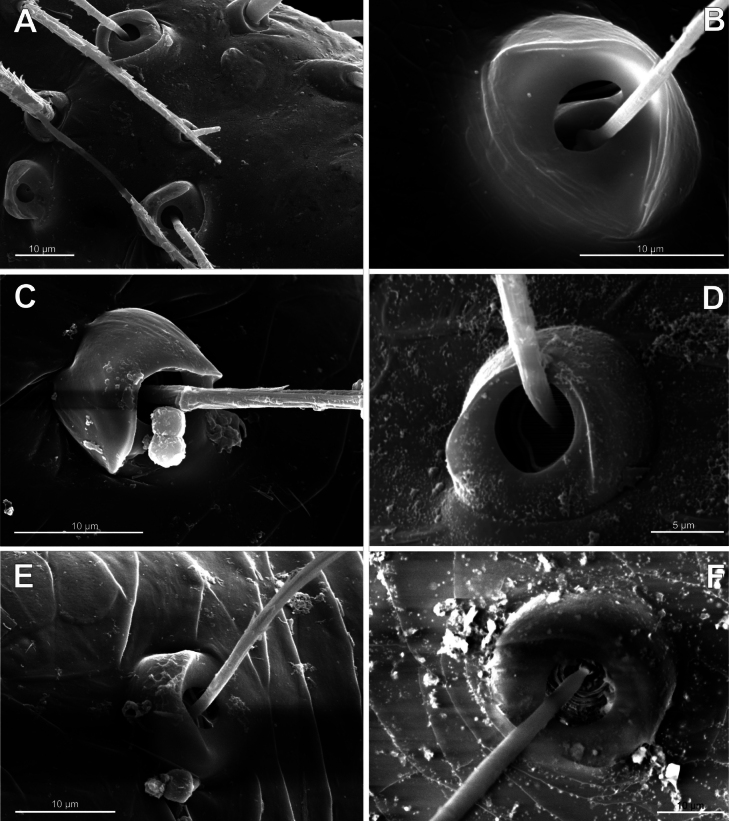
Bothria of ‘Symphytognathoid lineage’: Symphytognathidae, Comaromidae, Theridiosomatidae (Theridiosomatinae, Epeirotypinae, Ogulninae, Platoninae) **A***Symphytognathaglobosa*, ti 1 (E-type) **B***Comaromasimoni*, mt 1 (E-type) **C***Theridiosomaradiosum*, mt 1 (E-type) **D***Naatlo* sp., ti 1, (E-type) **E***Ogulnius* sp., mt 3 (E-type) **F***Platotroglodita*, ti 3 (A-type) (courtesy N. Dupérré).

4.5. Symphytognathidae (Sym).

The bothria of two symphytognathid genera have been examined here: *Symphytognatha* Hickman, 1931 (Fig. 18A) and *Anapistula* Gertsch, 1941 (image is not presented herein). In addition, the bothrium of Symphytognathidae gen. sp. has been previously illustrated (Lopardo and Hormiga 2015: fig. 119F, as SYMP-006-AUST). The bothria of the all symphytognathids seem to be highly uniform and belong to the ‘*Erigone*-type’ with an angled ridge.

4.6. Synaphridae (Syn).

The bothria of two synaphrid genera are studied here: *Synaphris* Simon, 1894 and *Cepheia* Simon, 1894 (Fig. 17E, F). In addition, the bothria of the three synaphrid genera have been figured previously: *Africepheia* Miller, 2007 (Miller 2007: figs 14, 15) and *Cepheia* (Lopardo and Hormiga 2007: fig. 25); in the genus *Synaphris* the bothria of the five species have been illustrated (Marusik and Lehtinen 2003: figs 22, 23; Marusik et al. 2005: fig. 30; Miller 2007: figs 50, 51, 59, 74; Lopardo et al. 2007: figs 15, 21, 22, 56). The bothria of the all *Synaphris* species studied belong to the ‘*Erigone*-type’ with a distinct angled ridge (Fig. 17E), whereas the bothria of both *Cepheia* (Fig. 17F) and *Africepheia* belong to the ‘*Argiope*-type’. Thus, the resemblance of bothrial shape confirms the close relationship between *Africepheia* and *Cepheia*, as suggested by Miller (2007).

4.7. Theridiosomatidae (Ths).

The bothria of five genera representing all four conventional theridiosomatid subfamilies have been examined: Theridiosomatinae: *Theridiosoma* O. Pickard-Cambridge, 1879 (Fig. 18C) and *Epilineutes* Coddington, 1986 (images are not presented herein); Epeirotypinae: *Naatlo* Coddington, 1986 (Fig. 18D); Ogulninae: *Ogulnius* O. Pickard-Cambridge, 1882 (Fig. 18E); Platoninae: *Plato* Coddington, 1986 (Fig. 18F). In addition, the bothria of the single theridiosomatid genus have been figured previously: *Cuacuba* Prete, Cizauskas & Brescovit, 2018 (Prete et al. 2018: fig. 7D). The bothria of the three theridiosomatid subfamilies, Theridiosomatinae, Epeirotypinae, and Ogulninae, seem to be highly uniform and belong to the ‘*Erigone*-type’ with an angled ridge (Fig. 18C–E). However, the bothria of the Platoninae, at least in the single genus examined, *Plato*, belong to the more advanced ‘*Argiope*-type’ (Fig. 18F). It should be noted that the bothria of *Cuacuba* also belong to the ‘*Argiope*-type’ (see Prete et al. 2018: fig. 7D). The authors have described this genus together with several new species of *Plato* but failed to indicate its subfamilial placement (Prete et al. 2018: 143). The diversity of the bothrial types in the ‘Symphytognathoid lineage’ is summarized in Fig. 30.

5. ‘Linyphioid lineage’, LI

5.1. Linyphiidae Blackwall, 1859 (Lin).

5.1.1. Erigoninae Emerton, 1882 [Erigo].

5.1.2. Linyphiinae Blackwall, 1859 [Linyp].

5.1.3. Micronetinae Hull, 1920 [Minet].

5.1.4. Mynogleninae Lehtinen, 1967 [Myngl].

5.1.5. Stemonyphantinae Wunderlich, 1986 [Stemo].

5.2. Pimoidae Wunderlich, 1986 (Pim). Conventional subfamilies/tribes are not established [Pimoi].

The ‘linyphioid lineage,’ forming, together with the ‘cyatholipoids’ and ‘theridioids,’ a distal branch of the superfamily Araneoidea (i.e., ‘clade 12’, or ‘araneoid sheet web weavers,’ according to Griswold et al. 1998: 16) represents the sister group of the latter pair (Griswold et al. 1998: fig. 7). Linyphiidae, the second most speciose family in the order, comprises five conventional subfamilies: the Holarctic Stemonyphantinae, the cosmopolitan Erigoninae, Linyphiinae, and Micronetinae, and the southern hemisphere Mynogleninae (Arnedo et al. 2009; Frick and Scharff 2014); the status of such linyphiid subfamilies as Dubiaraneinae Millidge, 1993, Ipainae Saaristo, 2007, and Sinopimoinae Li & Wunderlich, 2008, treated as distinct families by Eskov and Marusik (2023), requires further clarification. Pimoinае was established as a linyphiid subfamily to comprise the controversial genus *Pimoa* Chamberlin & Ivie, 1943 with a relict disjunct distribution (mountains of southern Europe, Himalaya, and western North America), previously transferred by Wunderlich (1979) to Linyphiidae from Metidae (= Tetragnathidae). Hormiga (1993) elevated it to a family, the sister group to Linyphiidae. Currently, only two geners are contained in the family Pimoidae, one of which is the enigmatic monotypic genus *Nanoa* Hormiga, Buckle & Scharff, 2005 from California and Oregon (Hormiga et al. 2005). Wunderlich (2008: 127) attributed *Nanoa* to the extinct family Pumiliopimoidae Wunderlich, 2008; it probably deserves the status of an independent linyphioid family or subfamily. Incidentally, the trichobothrial bases of the pimoid genera *Pimoa* and *Nanoa* differ dramatically from each other (see below).

5.1. Linyphiidae (Lin).

**Figure 19.**
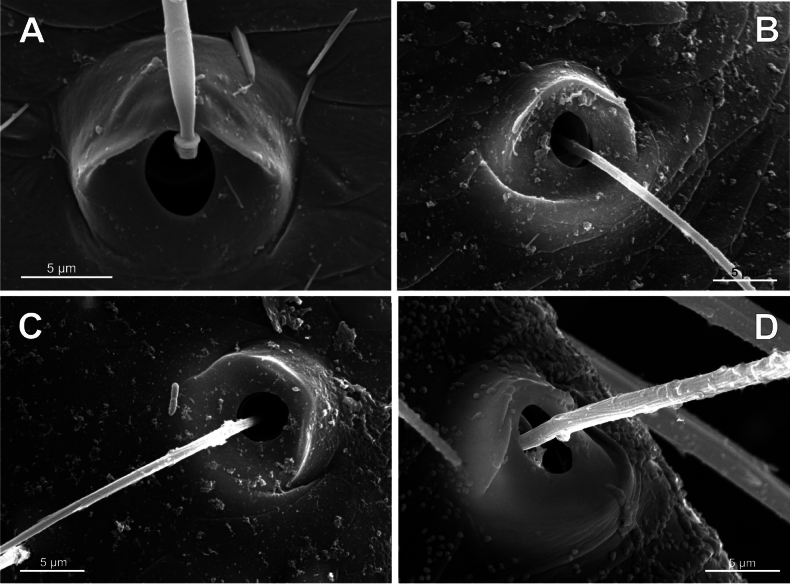
Bothria of ‘Linyphioid lineage’: Linyphiidae (Erigoninae) **A***Erigonedentipalpis*, mt 1 (E-type) **B***Pelecopsismengei*, ti 3 (E-type) **C***Scutpelecopsiswunderlichi*, ti 3 (E-type) **D***Minyrioluspusilus*, mt 1 (E-type).

**Figure 20.**
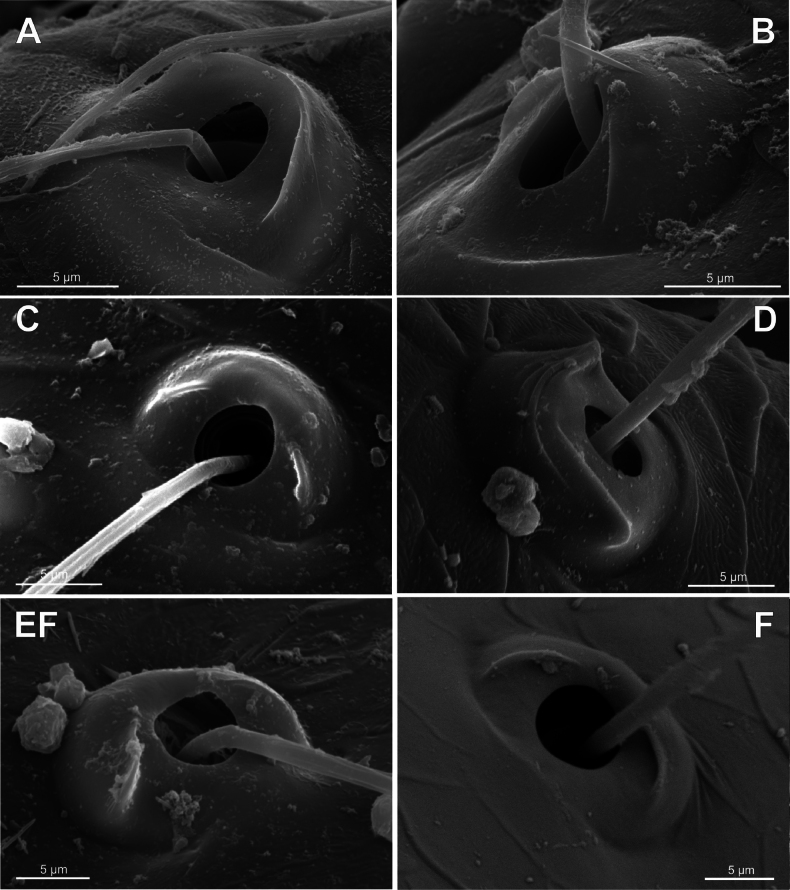
Bothria of ‘Linyphioid lineage’: Linyphiidae (Micronetinae, Linyphiinae). **A***Maropansibiricus*, ti 3 (E-type) **B***Agynetacauta*, ti 3 (E-type) **C***Micronetaviaria*, ti 1 (A-type) **D***Porrhommapygmaeum*, mt 2 (E-type) **E***Linyphiatriangularis*, ti 2 (A-type) **F***Allomengeascopigera*, mt 2 (A-type).

**Figure 21.**
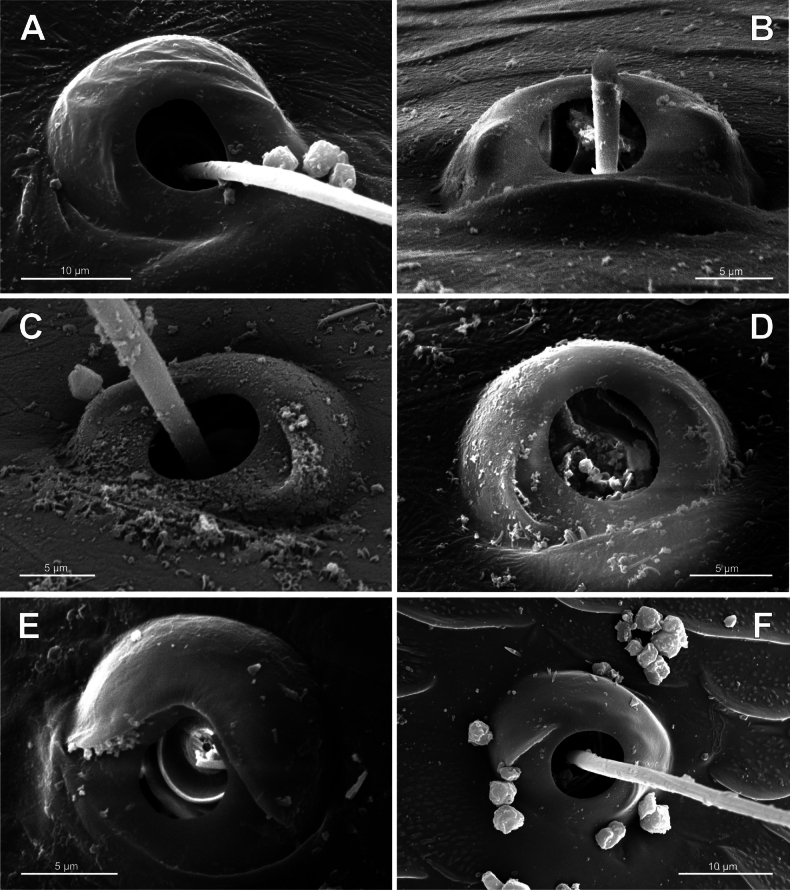
Bothria of ‘Linyphioid lineage’: Linyphiidae (Stemonyphantinae, Mynogleninae), Pimoidae**A***Stemonyphanteslineatus*, male palpal tibia (A-type) **B***Weintrauboainsularis*, ti 2 (A-type) **C***Haplinismundenia*, ti 3 (A-type) **D***Parafronetaconfusa*, ti 3 (A-type) **E***Nanoaenana*, ti3 (E-type) **F***Pimoarupicola*, ti 3 (A-type).

Bothria of 16 genera representing all five conventional linyphiid subfamilies are studied here: Erigoninae: *Erigone* Audouin, 1826; *Lophomma* Menge, 1868; *Pelecopsis* Simon, 1864; and *Scutpelecopsis* Marusik & Gnelitsa, 2009 (Fig. 19A–D) and *Minyriolus* Simon, 1884 (its images are not presented herein); Linyphiinae: *Allomengea* Strand, 1912; *Linyphia* Latreille, 1804; and *Porrhomma* Simon, 1884 (Fig. 20D–F) and *Lepthyphantes* Menge, 1866 (its images are not presented herein); Micronetinae: *Agyneta* Hull, 1911; *Maro* O. Pickard-Cambridge, 1907 and *Microneta* Menge, 1869 (Fig. 20A–C); Mynogleninae: *Haplinis* Simon, 1894 and *Parafroneta* Blest, 1979 (Fig. 21C, D); Stemonyphantinae: *Stemonyphantes* Menge, 1866 and *Weintrauboa* Hormiga, 2003 (Fig. 21A, B). In addition, the bothria of the two linyphiid genera have been figured earlier: *Masosundevalli* (Westring, 1851) (Lehtinen 1975: fig. 18), and *Orsonwelles* Hormiga, 2002, a giant Linyphiinae from the Hawaiian Islands (Hormiga 2002: fig. 46G).

The bothria of all the studied Erigoninae, as well as *Maso*, are highly uniform and belong to the ‘*Erigone*-type’ with the clearly angled ridge (Fig. 19A–D; Lehtinen 1975: fig. 18). In both subfamilies Linyphiinae and Micronetinae (it should be noted that they are often considered as tribes of the single subfamily Linyphiinae s. l.) the bothria of both the ‘*Erigone*-type’ and the ‘*Argiope*-type’ are combined, and the transverse ridges seem more rounded than angled. The ‘*Erigone*-type’ include *Maro*, *Agyneta*, and *Porrhomma* (Fig. 20A, B, and Fig. 20D, respectively); the ‘*Argiope*-type’ include *Microneta*, *Linyphia*, *Allomengea* (Fig. 20C, E, and Fig. 20F, respectively), *Lepthyphantes*; and *Orsonwelles* (Hormiga, 2002: fig. 46G). Finally, only the intermediate ‘*Argiope*-type’ bothria are found in the subfamilies Mynogleninae (Fig. 21C, D) and Stemonyphantinae (Fig. 21A, B).

5.2. Pimoidae (Pim).

The bothria of both pimoid genera *Nanoa* Hormiga, Buckle & Scharff, 2005 (Fig. 21E) and *Pimoa* Chamberlin & Ivie, 1943 (Fig. 21F) are studied here. They have dramatically different bothria: the ‘*Erigone*-type’ with the clearly angled ridge in *Nanoa* and the ‘*Argiope*-type’ in *Pimoa*. The diversity of the bothrial types in the ‘Linyphioid lineage’ is summarized in Fig. 31.

6 ‘Cyatholipoid lineage’, CY

6.1. Synotaxidae Simon, 1894 (Syt). Conventional subfamilies/tribes are not established [Sytax].

6.2. Physoglenidae Petrunkevitch, 1928 (Phy).

6.2.1. Pahorinae Forster, 1990 [Pahor].

6.2.2. Physogleninae Petrunkevitch, 1928 [Physo].

6.3. Cyatholipidae Simon, 1894 (Cya). Conventional subfamilies/tribes are not established [Cyath].

6.4. Nesticidae Simon, 1894 (Nes).

6.4.1. ‘*Eidmannella* clade’ [Eidma].

6.4.2. Nesticellini Lehtinen & Saaristo, 1980 [Necel].

6.4.3. Nesticini Simon, 1894 [Nesti].

The ‘cyatholipoid lineage’ (Cyatholipidae + Synotaxidae sensu Forster et al.1990) and the ‘theridioid lineage’ (Theridiidae + Nesticidae) were treated by Griswold et al. (1998: fig. 7) as sister groups forming the terminal clade of the araneoid cladogram: the ‘spineless femur clade’, clade 10. Ramírez et al. (2022: fig. 1) replaced nesticids from ‘theridioids’ to ‘cyatholipoids’ based on both morphological and molecular data. Kulkarni et al. (2021) nested cyatholipids together with Linyphiidae + Pimoidae based solely on molecular data, but this nesting lacks morphological support.

Forster et al. (1990) united the monotypic tribe Synotaxini (from theridiids), subfamily Physogleninae (from pholcids), and numerous newly described southern temperate taxa (New Zealand, Australia, and southern South America) in the ‘enlarged Synotaxidae’ with three subfamilies (Synotaxinae, Physogleninae, and Pahorinae), and recognized the Cyatholipidae as its sister group. However, Dimitrov et al. (2017) redelimited Synotaxidae to a single Neotropic genus, *Synotaxus* Simon, 1895, and united all the other synotaxids (sensu Forster et al. 1990) in the Physogleninae, which was then elevated to family and divided into the subfamilies Physogleninae and Pahorinae. Synotaxidae s. str. and Physoglenidae were nested in distant araneoid branches, as the sister groups of theridiosomatids and linyphioids, respectively (Dimitrov et al. 2017: fig. 2). Finally, Ramírez et al. (2022) transferred the controversial theridiid genus *Tekellina* Levi, 1957 and three nesticid genera, *Gaucelmus* Keyserling, 1884, *Hamus* Ballarin & Li, 2015, and *Nescina* Ballarin & Li, 2015, to the Synotaxidae s. str. based on both morphological and molecular data. In addition, Physoglenidae and the ‘newly enlarged Synotaxidae’ were recognized as independent but sister families (Ramírez et al. 2022: fig. 1). Based on the same morphological and molecular data, Nesticidae can be treated as the sister group of the pair Synotaxidae + Physoglenidae, but not of Theridiidae (Ramírez et al. 2022: fig. 1). Lehtinen and Saaristo (1980) established two tribes (instead of subfamilies) in Nesticidae, Nesticini and Nesticellini, but left the well-known Nearctic genera *Gaucelmus* and *Eidmannella* unclassified. *Gaucelmus*, as well as the recently described nesticid genera *Nescina* and *Hamus*, have already been transferred to Synotaxidae (see above); *Eidmannella* clearly differs from all ‘typical nesticids’ (Ballarin, pers. com. 18.08.2022) and is listed herein as an ‘*Eidmannella* clade’. The southern hemisphere Cyatholipidae (South Africa, Australia, and New Zealand) remains undivided into conventional subfamilies or tribes (Griswold 2001).

6.1. Synotaxidae (Syt).

**Figure 22.**
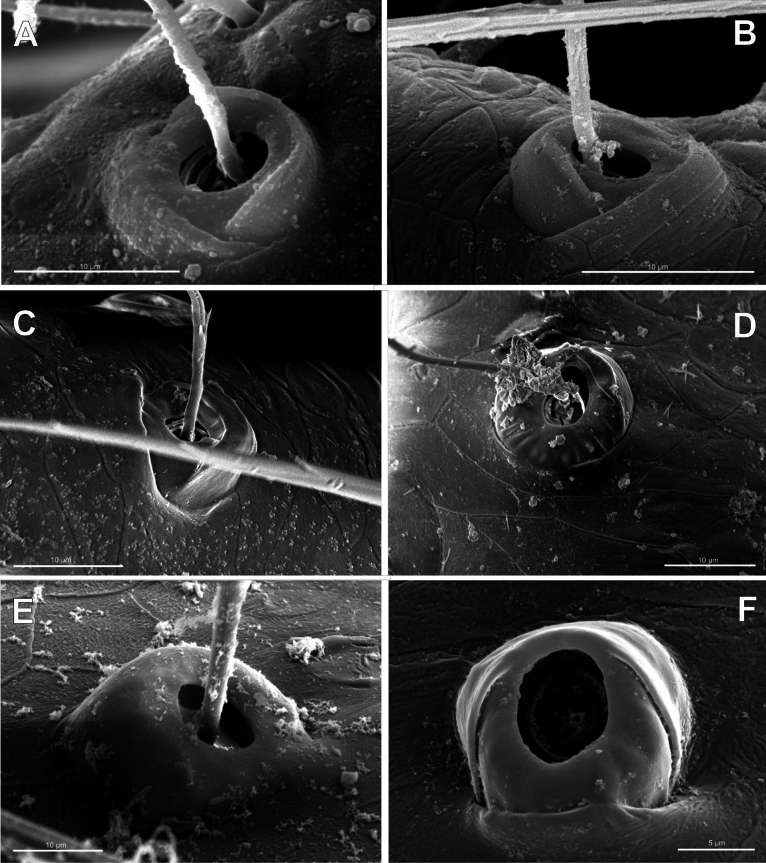
Bothria of ‘Cyatholipoid lineage’: Synotaxidae**A***Tekellinasadamotoi*, mt 1 (E-type) **B** ‘Tekellina’ araucana, ti 3 (E-type) **C***Nescina* sp., ti 3 (E-type) **D***Hamuscornutus*, ti 2 (E-type) (courtesy F. Ballarin) **E***Gaucelmus* sp., ti 3 (A-type) **F***Synotaxus* sp., ti 1 (A-type).

Bothria of all six synotaxid genera are studied here: *Gaucelmus* Keyserling, 1884; *Hamus* Ballarin & Li, 2015; *Nescina* Lin, Ballarin & Li, 2016; *Synotaxus* Simon, 1895; *Tekellina* Levi, 1957; and ‘Tekellina’ araucana Marusik, Eskov & Ramírez, 2022, probably representing an undescribed genus (Fig. 22A–F). In addition, the bothria of ‘Tekellina’ araucana have been figured (Ramírez et al. 2022: fig. 5C). Bothria of the two types are presented in Synotaxidae: the ‘*Erigone*-type’ in *Tekellina*, ‘Tekellina’ araucana, *Nescina*, and *Hamus* (Fig. 22A, B, C, and Fig. 22D, respectively), and the ‘*Argiope*-type’ in *Gaucelmus* and *Synotaxus* (Fig. 22E and Fig. 22F, respectively).

6.2. Physoglenidae (Phy).

**Figure 23.**
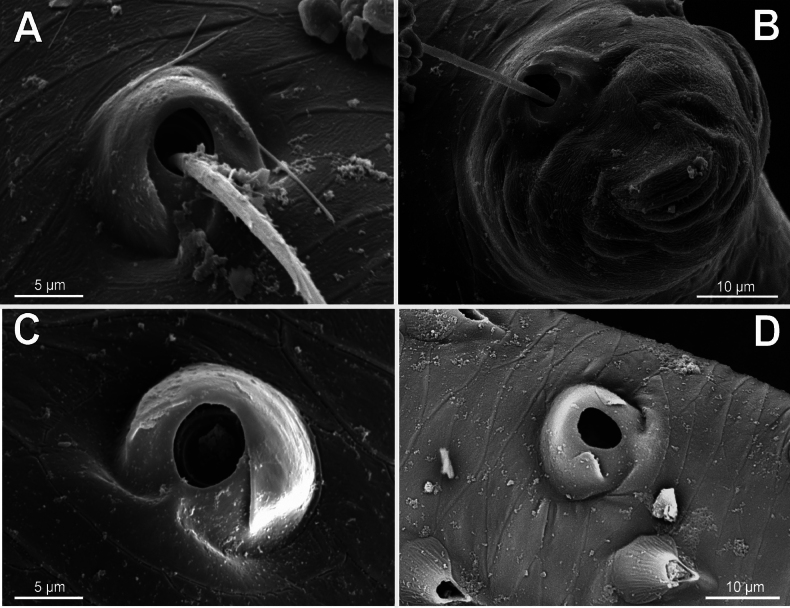
Bothria of ‘Cyatholipoid lineage’: Physoglenidae (Pahorinae, Physogleninae) **A***Pahoramurihiku*, ti 2 (E-type) **B***Pahoramurihiku*, trichobothrium-bearing spur on male palpal tibia (E-type) **C***Tupuabisetosa*, ti 2 (A-type) **D***Physoglenespuyehue*, mt 3 (A-type).

The bothria of three genera representing both physoglenid subfamilies are studied here: Pahorinae: *Pahora* Forster, 1990 (Fig. 23A, B); Physogleninae: *Tupua* Platnick, 1990 and *Physoglenes* Simon, 1904 (Fig. 23C, D). In addition, the bothria of the three physoglenid genera have been figured earlier: *Pahora*, three species (Forster et al. 1990: figs 144, 163, 178) and the *Mangua* Forster, 1990, three species (Forster et al. 1990: figs 260, 276, 299) from the Pahorinae, and the *Meringa* Forster, 1990, four species (Forster et al. 1990: figs 34, 53, 65, 78) from the Physogleninae. Each of the two physoglenid subfamilies has its own bothrial type. Both studied genera of Pahorinae, *Pahora* (Fig. 23A, B; Forster et al. 1990: fig. 163) and *Mangua* (Forster et al. 1990: fig. 299), possess the ‘*Erigone*-type’ of bothria. All studied genera of the Physogleninae, *Tupua* (Fig. 23C), *Physoglenes* (Fig. 23D), and *Meringa* (Forster et al. 1990: figs 53, 54) possess the ‘*Argiope*-type’ of bothria; this character was included in the subfamily diagnosis by Forster et al. (1990: 5): “Trichobothria present at least on tibiae, bases with only traces of posterior hood”. It should be noted that in the genus *Pahora* the “modified trichobothria sometimes present on male palpal tibia” (Forster et al. 1990: 38), and the male palpal tibia is provided with a particular structure, named the ‘trichobothrium-bearing spur’ (Forster et al. 1990: 41). The transverse ridge of the ‘*Erigone*-type’ bothria on leg joints is rounded (Fig. 23A), whereas a ridge of the bothria on ‘trichobothrium-bearing spur’ is clearly angled (Fig. 23B; Forster et al. 1990: 144, 163). This case resembles to the mimetine genus *Ero* (see above), where the bothria on the leg joints and the male palp tibia belong to the different bothrial types (Fig. 6C and Fig. 6D, respectively).

6.3. Cyatholipidae (Cya).

**Figure 24.**
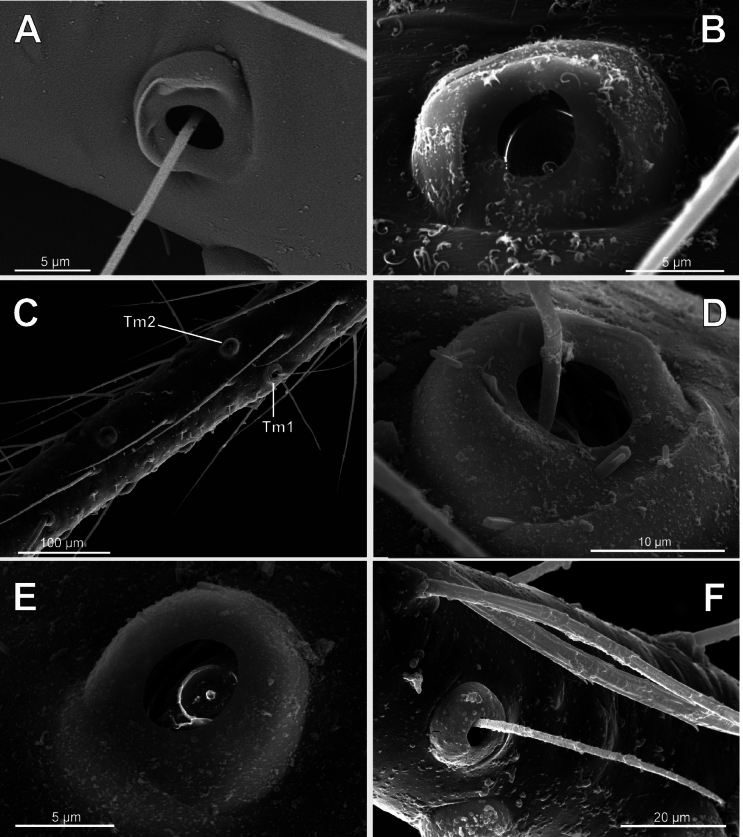
Bothria of ‘Cyatholipoid lineage’: Cyatholipidae**A***Matilda* sp.1, ti 2 (E-type) (courtesy R. Raven) **B***Tekellaabsidata*, ti 2 (A-type) **C***Teemenaarussilvestris*, ti 3 (Tm1 and Tm2, arrows – two bothria of A-type, but differs in form) **D** the same, Tm1 (A-type) **E** the same, Tm2 (A-type) **F***Ilisoa* sp., ti 2 (T-type).

The bothria of four cyatholipid genera are studied here: *Matilda* Forster, 1988; *Tekella* Urquhart, 1894; *Teemenaarus* Davies, 1978; and *Ilisoa* Griswold, 1987 (Fig. 24A–F). In addition, the bothria of the 5 cyatholipid genera have been figured previously: *Tekella*, *Teemenaarus*, *Cyatholipus* Simon, 1894 and *Matilda* (Forster 1988: figs 26, 30, 31, and 140, respectively), and *Pembatatu* Griswold, 2001 (Griswold 2001: fig. 6B). Forster (1988: 11) has included the ‘*Argiope*-type’ of bothria, described as “Bothria with the posterior hood reduced to two small ridges or absent”, in the diagnosis of the family. The intermediate ‘*Argiope*-type’ of bothria indeed seems most usual in the cyatholipids: *Tekella* (Fig. 24B; Forster 1988: fig. 26), *Teemenaarus* (Fig. 24C–E; Forster 1988: fig. 30), and *Cyatholipus* (Forster 1988: fig. 31). However, the ancestral ‘*Erigone*-type’ of bothria is recorded in *Matilda* (Fig. 24A; Forster 1988: fig. 140), as well as the advanced ‘*Theridion*-type’ in *Ilisoa* (Fig. 24F) and *Pembatatu* (Griswold 2001: fig. 6B). So, the complete set of the three bothrial types is presented in the Cyatholipidae. It should be noted that bothrium shape varies unusually in some cyatholipid genera, e.g., in *Matilda* (cf. *Matilda* sp. 1: Fig. 24A, and *Matildaaustralia*: Forster 1988: fig. 140). Moreover, sometimes the bothrium shape varies even in the same cyatholipid specimen (see *Teemenaarussilvestris*: Fig. 24C–E).

6.4. Nesticidae (Nes).

**Figure 25.**
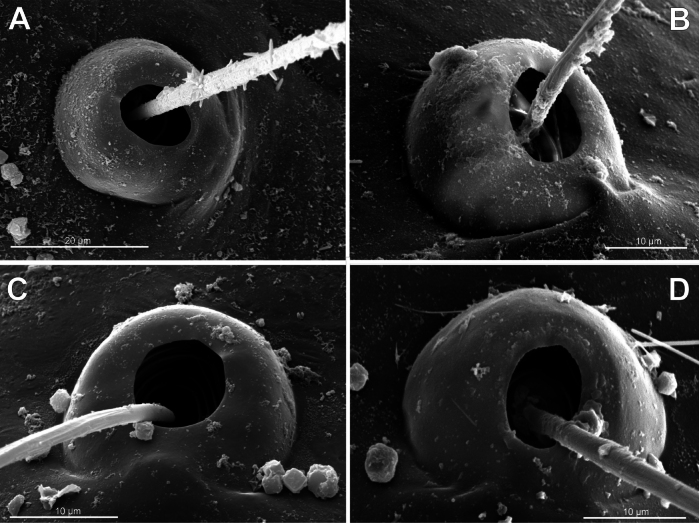
Bothria of ‘Cyatholipoid lineage’: Nesticidae (Nesticini, Nesticellini, ‘*Eidmannella* clade’) **A***Aituariapontica*, mt 1 (T-type) **B***Daginesticusmamajevae*, ti 3 (T-type) **C***Nesticellaterrestris*, ti 3 (T-type) **D***Eidmannellapallida*, ti 2 (A-type).

The bothria of four genera representing all three nesticid subtaxa, distinguished now, are studied here: Nesticini: *Aituaria* Esyunin & Efimik, 1998 and *Daginesticus* Fomichev, Ballarin & Marusik, 2022 (Fig. 25A, B); Nesticellini: *Nesticella* Lehtinen & Saaristo, 1980 (Fig. 25C); ‘*Eidmannella* clade’: *Eidmannella* Roewer, 1935 (Fig. 25D). The bothria of both Nesticini and Nesticellini are highly uniform and belong to the ‘*Theridion*-type’. However, bothria of ‘*Eidmannella* clade’ belong to the ‘*Argiope*-type’. The diversity of the bothrial types in the ‘Cyatholipoid lineage’ is summarized in Fig. 32.

7. ‘Theridioid lineage’, TH:

7.1. Theridiidae Sundevall, 1833 (Thr).

7.1.1. Argyrodinae Simon, 1881 [Argyr].

7.1.2. Hadrotarsinae Thorell, 1881 [Hadro].

7.1.3. Latrodectinae Petrunkevitch, 1928 [Latro].

7.1.4. Pholcommatinae Simon, 1894 [Pholc].

7.1.5. Spintharinae Simon, 1894 [Spint].

7.1.6. Theridiinae Sundevall, 1833 [Thrid].

7.1.7. Phoroncidini O. Pickard-Cambridge, 1874 [Phorn].

After the relocation of Nesticidae by Ramírez et al. (2022) to ‘Cyatholipoids’, the sister lineage of the ‘spineless femur clade,’ Theridiidae remained the only member of the ‘theridioid line’ (Griswold et al. 1998: fig. 7). Agnarsson (2004: fig. 105) recognized six theridiid subfamilies: Hadrotarsinae, Latrodectinae, Spintharinae, Pholcommatinae, Argyrodinae, and Theridiinae. However, the subfamily affiliation of some theridiid genera remained uncertain; and we preferred to listing herein, e.g., *Phoroncidia* Westwood, 1835 in old Simon’s (1894) tribe Phoroncidini. Hadrotarsines were formerly described as an independent family and listed in Haplogynae (see discussions in Forster et al. 1990 and Agnarsson 2004), but today they are recognized as a theridiid subfamily.

7.1. Theridiidae (Thr).

**Figure 26.**
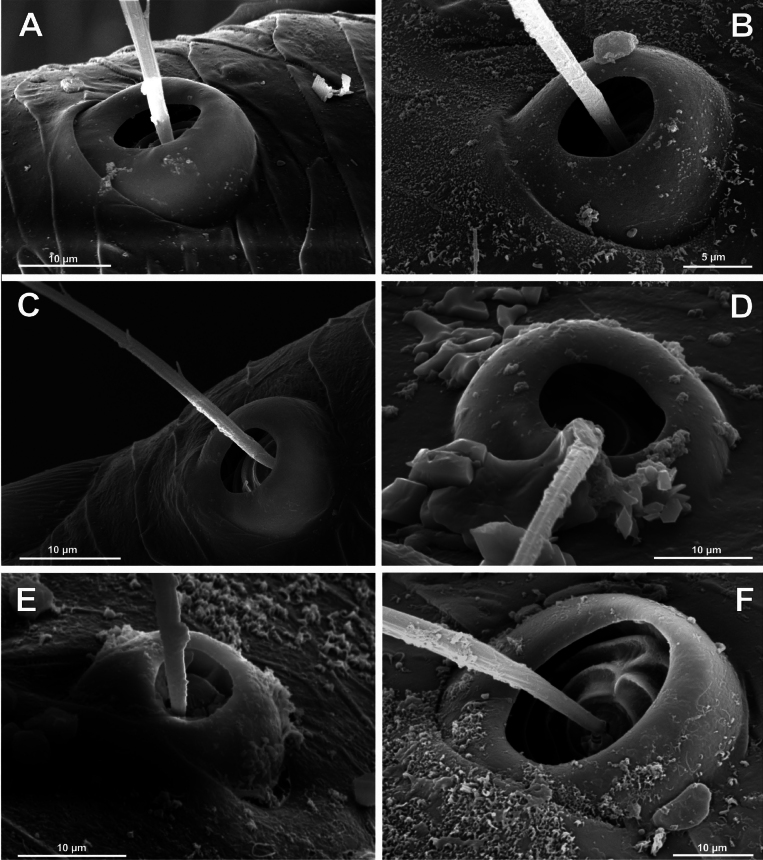
Bothria of ‘Theridioid lineage’: Theridiidae (Hadrotarsinae, Argyrodinae, Latrodectinae, Spintharinae, Theridiinae) **A***Euryopisflavomaculata*, mt 2 **B***Phycosoma* sp., ti 3 **C***Argyrodes* sp., ti 3 **D***Latrodectustredecimguttatus*, ti 3 **E***Episinusangulatus*, ti 3 **F***Theridiontinctum*, ti 3.

**Figure 27.**
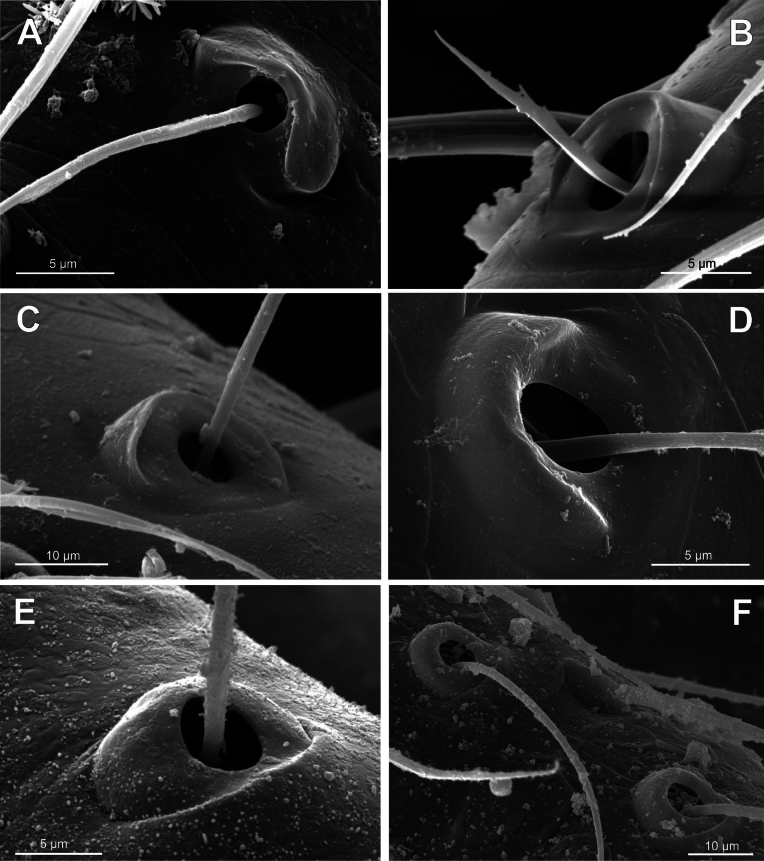
Bothria of ‘Theridioid lineage’: Theridiidae (Pholcommatinae, Phoroncidini) **A***Carniellanepalensis*, ti 3 (E-type) **B***Theonoeminutissima*, ti 1 (E-type) **C***Robertuslividus*, ti 3 (E-type) **D***Pholcommagibbum*, ti 3 (A-type) **E***Glebychminutissimus*, ti 1 (T-type) **F***Phoroncidia* sp., ti 1 (T-type).

The bothria of 13 genera representing all six conventional theridiid subfamilies and the one tribe of uncertain position are studied here: Hadrotarsinae: *Euryopis* Menge, 1868 and *Phycosoma* O. Pickard-Cambridge, 1880 (Fig. 26A, B); Latrodectinae: *Latrodectus* Walckenaer, 1805 (Fig. 26D) and *Crustulina* Menge, 1868 (which images are not presented herein); Spintharinae: *Episinus* Walckenaer, 1809 (Fig. 26E); Pholcommatinae: *Carniella* Thaler & Steinberger, 1988; *Theonoe* Simon, 1881; *Robertus* O. Pickard-Cambridge, 1879; *Pholcomma* Thorell, 1869; and *Glebych* Eskov & Marusik, 2021 (Fig. 27A–E); Argyrodinae: *Argyrodes* Simon, 1864 (Fig. 26C); Theridiinae: *Theridion* Walckenaer, 1805 (Fig. 26F); and Phoroncidini: *Phoroncidia* Westwood, 1835 (Fig. 27F). In addition, the bothria of the five theridiid genera have been figured earlier: *Anelosimus* Simon, 1891, *Argyrodes*, *Spintharus* Hentz, 1850, and *Stemmops* O. Pickard-Cambridge, 1894 (Agnarsson 2004: figs 24C, 31G, 69E, and 74D, respectively), and *Knoflachia* Marusik & Eskov, 2024 (Marusik and Eskov 2024: fig. 5F). The bothria of the almost all theridiid subfamilies and tribes (Hadrotarsinae, Latrodectinae, Spintharinae, Argyrodinae, Theridiinae, and Phoroncidini) are highly uniform and belong to the advanced ‘*Theridion*-type’ (Figs 26A–F, 27F). The single remarkable exception is the subfamily Pholcommatinae, having the complete set of the all three bothrial types: the ancestral ‘*Erigone*-type’ in *Carniella*, *Theonoe* and *Robertus* (Fig. 27A, B, and Fig. 27C, respectively); the intermediate ‘*Argiope*-type’ in *Pholcomma* (Fig. 27D); and the advanced ‘*Theridion*-type’ in *Glebych* (Fig. 27F). The diversity of the bothrial types in the ‘Theridioid lineage’ is summarized in Fig. 32.

## ﻿Discussion

**Figure 28.**
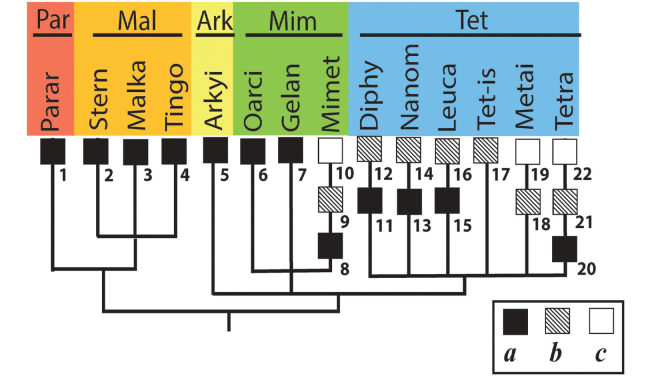
Distribution of the three main bothrial types in the subfamilies/tribes of the ‘Tetragnathoid branch’ (the ‘Malkariod’ and the ‘Tetragnathoid’ lineages). Abbreviations: a – the ancestral ‘*Erigone*-type’, b – the intermediate ‘*Argiope*-type’, c – the advanced ‘*Theridion*-type’; Ark – Arkyidae (Arkyi – ‘Arkyinae’); Mal – Malkaridae (Malka – Malkarinae, Stern – Sternoidinae, Tingo – Tingotinginae); Mim – Mimetidae (Gelan – Gelanorinae, Mimet – Mimetinae, Oarci – Oarcinae); Par – Pararchaeidae (Parar – ‘Pararchaeinae’); Tet – Tetragnathidae (Diphy – Diphyainae, Leuca – Leucauginae, Metai – Metainae, Nanom – Nanometinae, Tetra – Tetragnathinae). The numbers refer to the studied genera: 1 *Anarchaea*, *Flavarchaea*; 2 *Chilenodes*; 3 *Malkara*; 4 *Tingotingo*; 5 *Arkys*; 6 *Oarces*; 7 *Gelanor*; 8 *Ero* (male palp tibia); 9 *Ero* (leg joints); 10 *Australomimetus*; 11 *Chrysometa*, Diphyainae gen. sp.; 12 *Diphya*; 13 *Pinkfloydia*; 14 *Nanometa*, *Orsinome*; 15 *Leucauge*; 16 *Metleucauge*; 17 *Azilia*; 18 *Metellina*; 19 *Meta*; 20 *Cyrtognatha*; 21 *Allende*, *Mollemeta*; 22 *Pachygnatha*, *Tetragnatha*.

**Figure 29.**
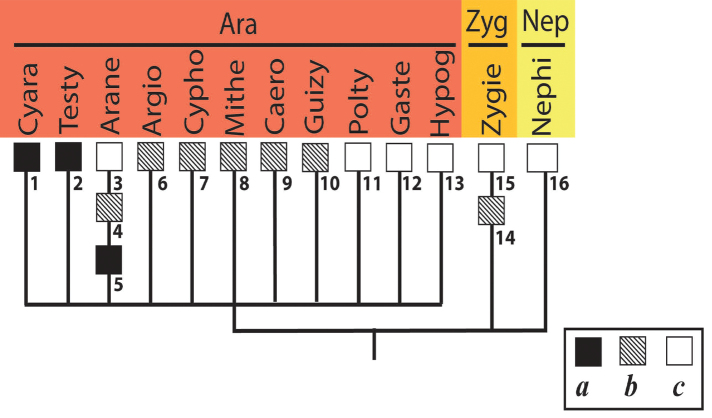
Distribution of the three main bothrial types in the subfamilies/tribes of the ‘Araneoid lineage’. Abbreviations: a – the ancestral ‘*Erigone*-type’, b – the intermediate ‘*Argiope*-type’, c – the advanced ‘*Theridion*-type’; Ara – Araneidae (Arane – Araneinae, Argio – Argiopinae, Caero – Caerostrini, Cyara – Cyrtarachninae, Cypho – Сyrtophorinae, Gaste – Gasteracanthinae, Guizy – Guizygiellinae; Hypog – Hypognathini, Mithe – Micratheninae, Polty – Poltyini, Testu – Testudinarini); Nep – Nephilidae (Nephi – ‘Nephilinae’); Zyg – Zygiellidae (Zygie – ‘Zygiellinae’). The numbers refer to the studied genera:1 *Cyrtarachne*, *Chorizopes*; 2 *Melychiopharis*; 3 *Araneus*; 4 *Larinia*, *Mangora*, *Cyclosa*; 5 *Singa, Hypsosinga*; 6 *Argiope*; 7 *Cyrtophora*; 8 *Micrathena*; 9 *Caerostris*; 10 *Guizygiella*; 11 *Poltys*; 12 *Gasteracantha*; 13 *Hypognatha*;14 *Zygiellax-notata*, *Leviellus*; 15 ‘Zygiella’ atrica, *Parazygiella*; 16 *Nephila*, *Nephilengys*.

**Figure 30.**
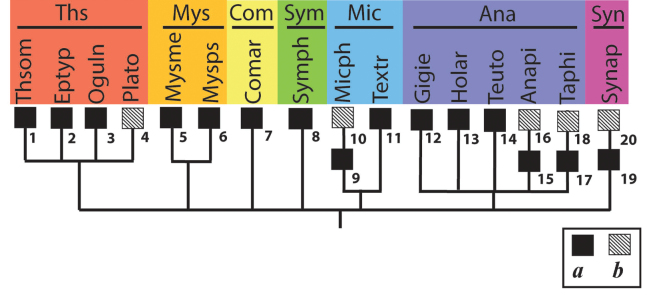
Distribution of the two main bothrial types in the subfamilies/tribes of the ‘Symphytognathoid lineage’. Abbreviations: a – the ancestral ‘*Erigone*-type’, b – the intermediate ‘*Argiope*-type’; Ana – Anapidae (Anapi – Anapinae, Gigie – Gigiellinae, Holar – Holarchaeinae, Taphi – Taphiassinae, Teuto – Teutoniellinae);Com – Comaromidae (Comar – ‘Comarominae’);Mic – Micropholcommatidae (Micph – Micropholcommatinae, Textr – Textricellinae);Mys – Mysmenidae (Mysme – Mysmeninae, Mysps – Mysmenopsinae);Sym – Symphytognathidae (Symph – ‘Symphytognathinae’);Syn – Synaphridae (Synap – ‘Synaphrinae’);Ths – Theridiosomatidae (Eptyp – Epeirotypinae, Oguln – Ogulninae, Plato – Platoninae, Thsom – Theridiosomatinae). The numbers refer to the studied genera (in square brackets a literature data): 1 *Theridiosoma*; 2 *Naatlo*; 3 *Ogulnius*; 4 *Plato*, [*Cuacuba*]; 5 *Mysmena*, *Microdipoena*; 6 *Mysmenopsis*, *Isela*; 7 *Comaroma*, *Balticoroma*; 8 *Symphytognatha*; 9 *Micropholcomma*, *Tricellina*, [*Pua*]; 10 *Plectochetos*, *Austropholcomma*, [*Patelliella*]; 11 *Rayforstia*, *Eterosonycha*, *Epigastrina*, *Raveniella*, *Normplatnicka*, *Eperiella*, [*Algidiella*, *Taliniella*, *Tinytrella*]; 12 *Gigiella*; 13 *Holarchaea*; 14 *Teutoniella*; 15 *Acrobleps*, *Minanapis*, *Montanapis*, *Crassanapis*, *Sheranapis*, *Elanapis*, *Sofanapis*, *Hickmanapis*, *Zealanapis*, *Zangherella*, [*Risdonius*]; 16 *Pseudanapis*; 17 [*Olgania*]; 18 *Taphiassa*; 19 *Synaphris*; 20 *Cepheia*.

**Figure 31.**
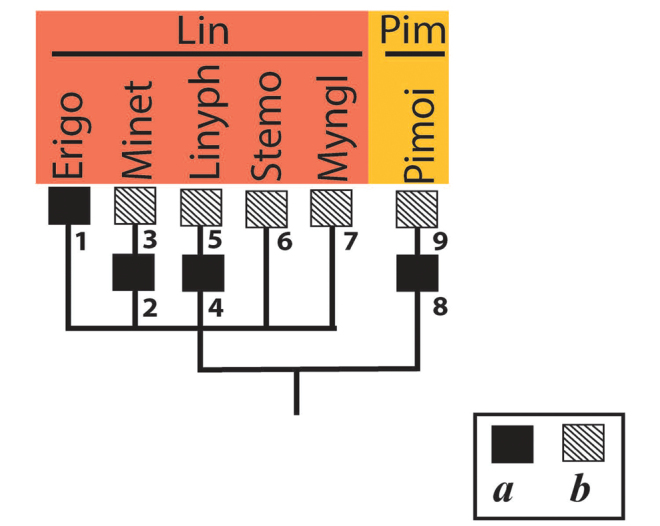
Distribution of the two main bothrial types in the subfamilies/tribes of the ‘Linyphioid lineage’. Abbreviations: a – the ancestral ‘*Erigone*-type’, b – the intermediate ‘*Argiope*-type’; Lin – Linyphiidae (Erigo – Erigoninae, Linyp – Linyphiinae, Minet – Micronetinae, Myngl – Mynogleninae, Stemo – Stemonyphantinae); Pim – Pimoidae (Pimoi – ‘Pimoidae’). The numbers refer to the studied genera: 1 *Erigone*, *Pelecopsis*, *Scutpelecopsis*, *Minyriolus*, *Lophomma*; 2 *Maro*, *Agyneta*; 3 *Microneta*; 4 *Porrhomma*; 5 *Linyphia*, *Allomengea*, *Lepthyphantes*; 6 *Stemonyphantes*, *Weintrauboa*; 7 *Haplinis*, *Parafroneta*; 8 *Nanoa*; 9 *Pimoa*.

**Figure 32.**
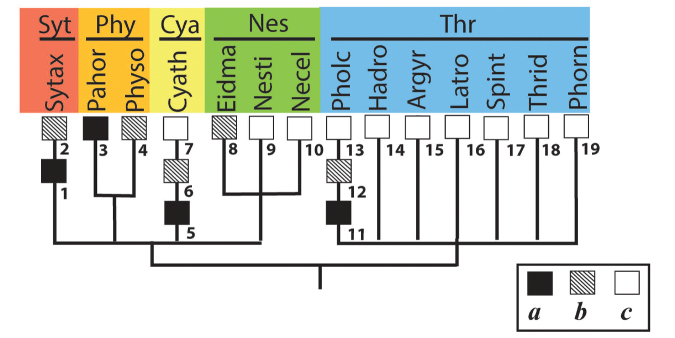
Distribution of the three main bothrial types in the subfamilies/tribes of the ‘Spineless femur clade’ (the ‘Cyatholipoid’ and the ‘Theridioid’ lineages). Abbreviations: a – the ancestral ‘*Erigone*-type’, b – the intermediate ‘*Argiope*-type’, c – the advanced ‘*Theridion*-type’; Cya – Cyatholipidae (Cyath – ‘Cyatholipidae’); Nes – Nesticidae (Eidma – ‘*Eidmannella* clade’, Necel – Nesticellini, Nesti – Nesticini); Phy – Physoglenidae (Pahor – Pahorinae, Physo – Physogleninae); Syt – Synotaxidae (Sytax – ‘Synotaxidae’); Thr – Theridiidae (Argyr – Argyrodinae, Hadro – Hadrotarsinae, Latro – Latrodectinae, Pholc – Pholcommatinae, Phorn – Phoroncidini, Spint – Spintharinae, Thrid – Theridiinae).The numbers refer to the studied genera (in square brackets a literature data): 1 *Tekellina*, ‘Tekellina’ araucana, *Nescina*, *Hamus*; 2 *Gaucelmus*, *Synotaxus*; 3 *Pahora*, [*Mangua*]; 4 *Tupua*, *Physoglenes*, [*Meringa*]; 5 *Matilda*; 6 *Tekella*, *Teemenaarus*; 7 *Ilisoa*; 8 *Eidmannella*; 9 *Aituariapontica*, *Daginesticus*; 10 *Nesticella*; 11 *Carniella*, *Theonoe*, *Robertus*; 12 *Pholcomma*; 13 *Glebych*; 14 *Euryopis*, *Phycosoma*; 15 *Argyrodes* 16 *Latrodectus*, *Crustulina*; 17 *Episinus*; 18 *Theridion*; 19 *Phoroncidia*.

Distribution, at the subfamily/tribe level, of the three main bothrial types (the ancestral ‘*Erigone*-type’, the advanced ‘*Theridion*-type’, and the intermediate ‘*Argiope*-type’) in the seven main lineages of Araneoidea (‘malkariods’, ‘tetragnathoids’, ‘araneoids’, ‘symphytognathoids’, ‘linyphioids’, ‘cyatholipoids’ and ‘theridioids’) is summarized in Figs 28–32. It should be emphasized that these are not cladograms in the strict sense: the families/subfamilies within of the seven main lineages (Fig. 1) are not subordinated. They are arranged in one row so that taxa with the ancestral type of bothria are on the left side of the row, and those with an advanced type are on the right.

A subfamily or tribe can have a single bothrial type or a sequence of several types. The sequence can be complete, comprising all three types (from the ancestral, most complicated ‘*Erigone*-type’ to the advanced, simplest ‘*Theridion*-type’, via the intermediate ‘*Argiope*-type’), or shortened to two types: the basal (from the ancestral ‘*Erigone*-type’ to the intermediate ‘*Argiope*-type’) or the terminal (from the intermediate ‘*Argiope*-type’ to the advanced ‘*Theridion*-type’). We will name them further ‘the complete E-A-T sequence’, ‘the shortened basal E-A sequence’ and ‘the shortened terminal A-T sequence’, respectively.

It should be emphasized that not a single combination of the ancestral ‘*Erigone*-type’ and the advanced ‘*Theridion*-type’, without the intermediate ‘*Argiope*-type’, was found in the any of the 61 studied subfamilies/tribes (Figs 28–32). It confirms, in our opinion, that we are dealing exactly with sequences, not with ‘mechanical sets’ of bothrial types.

In the ‘Malkaroid lineage’ (Fig. 28), there are no bothrial sequences, and all its members (the malkarids Malkarinae, Sternoidinae and Tingotinginae, and Pararchaeidae) have a single bothrial type: the ancestral ‘*Erigone*-type’.

In the ‘Tetragnathoid lineage’ (Fig. 28), there are two complete E-A-T sequences: in the mimetids Mimetinae and the tetragnathids Tetragnathinae. The remaining mimetids, Oarcinae and Gelanorinae, as well as the Arkyidae, have the ancestral ‘*Erigone*-type’ only. By contrast, the remaining tetragnathids do not have a single bothrial type, but bothrial sequences: the shortened basal E-A sequences (in Diphyainae, Nanometinae and Leucauginae) and the shortened terminal A-T sequence in Metainae.

In the ‘Araneoid lineage’ (Fig. 29), only two sequences are present: the complete E-A-T sequence in the araneids Araneinae, and the shortened terminal A-T sequence in Zygiellidae. All the remaining taxa have a single bothrial type: two have the ‘*Erigone*-type’ (the araneids Cyrtarachninae and Testudinarini), four have the ‘*Argiope*-type’ (the araneids Argiopinae, Cyrtophorinae, Micratheninae, and Caerostrini), and four have the ‘*Theridion*-type’ (the araneids Gasteracanthinae, Hypognathini and Poltyini, and in Nephilidae).

In the ‘Symphytognathoid lineage’ (Fig. 30), the complete E-A-T sequences are absent, and a single type of the bothrial sequence, the shortened basal E-A sequence, is present in four taxa (in the anapids Anapinae and Taphiassinae, the micropholcommatids Micropholcommatinae, and in Synaphridae). The majority of the symphytognathoid taxa have a single bothrial type: ten have the ‘*Erigone*-type’ (the theridiosomatids Theridiosomatinae, Epeirotypinae, and Ogulninae, the mysmenids Mysmeninae and Mysmenopsinae, Symphytognathidae, Comaromidae, the micropholcommatids Textricellinae, and the anapids Holarchaeinae, Teutoniellinae, and Gigiellinae), and one has the ‘*Argiope*-type’ (the theridiosomatids Platoninae).

In the ‘Linyphioid lineage’ (Fig. 31), the complete E-A-T sequences are also absent. There are three shortened basal E-A sequences (in the linyphiids Micronetinae and Linyphiinae, and in Pimoidae). The remaining taxa have a single bothrial type: the ‘*Erigone*-type’ in the linyphiids Erigoninae, and the ‘*Argiope*-type’ in the linyphiids Stemonyphantinae and Mynogleninae.

In the ‘Cyatholipoid lineage’ (Fig. 32), a high diversity of bothrial types and their sequences is found: the complete E-A-T sequence in Cyatholipidae; the shortened basal E-A sequence in Synotaxidae; the ‘*Erigone*-type’ in the physoglenids Physogleninae; the ‘*Argiope*-type’ in the physoglenids Pahorinae and nesticids of the ‘*Eidmannella* clade’; and the ‘*Theridion*-type’ in the nesticids Nesticini and Nesticellini.

**Figure 33.**
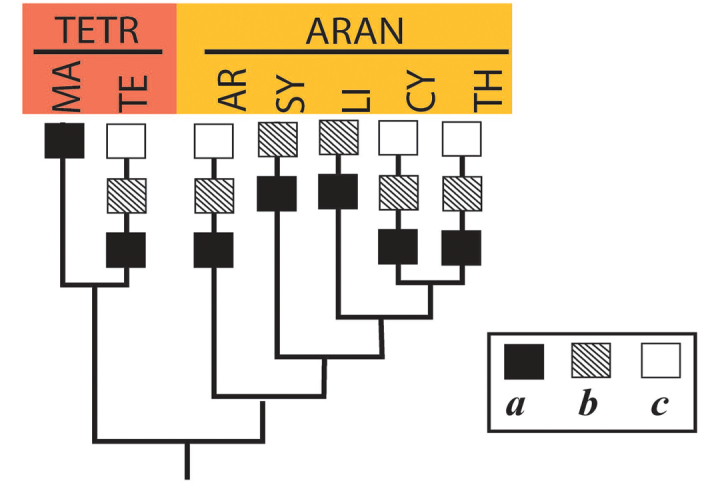
Distribution of the three main bothrial types in the main divisions of the superfamily Araneoidea, ‘branches’ and ‘lineages’. Abbreviations: a – the ancestral ‘*Erigone*-type’; b – the intermediate ‘*Argiope*-type’; c – the advanced ‘*Theridion*-type’. ARAN – Araneoid branch; TETR – Tetragnathoid branch; AR – Araneoid lineage; CY – Cyatolipoid lineage; LI – Linyphioid lineage; MA – Malkaroid lineage; SY – Symphytognathoid lineage; TE – Tetragnathoid lineage; TH – Theridioid lineage.

In the ‘Theridioid lineage’ (Fig. 32), the picture seems unique. Almost all the subfamilies/tribes (Hadrotarsinae, Latrodectinae, Spintharinae, Argyrodinae, Theridiinae, and Phoroncidini) have uniform bothria of the ‘*Theridion*-type’. However, there is a single remarkable exception: the subfamily Pholcommatinae has a complete E-A-T sequence. These data are summarized in Table 1 and Fig. 33.

**Table 1.** Distribution, at the subfamily/tribe level, of the three main bothrial types and their sequences in the seven main lineages of Araneoidea. AR – ‘araneoids’, CY – ‘cyatholipoids’, LI – ‘linyphioids’, MA – ‘malkariods’, SY – ‘symphytognathoids’, TE – ‘tetragnathoids’, and TH – ‘theridioids’.

	MA	TE	AR	SY	LI	CY	TH	Σ
ancestral ‘*Erigone*-type’, E	4	3	2	10	1	1	–	21
shortened basal E-A sequence	–	3	–	4	3	1	–	12
intermediate ‘*Argiope*-type’, A	–	–	4	1	2	2	–	9
complete E-A-T sequence	–	2	1	–	–	1	1	5
shortened terminal A-T sequence	–	1	1	–	–	–	–	2
advanced ‘*Theridion*-type’, T	–	–	4	–	–	2	6	12

We can see (Fig. 33) the parallel evolutionary trends of the step-by-step replacing the basal ‘*Erigone*-type’ of bothria by the more advanced ‘*Argiope*-type’ and, finally, by the terminal ‘*Theridion*-type’ in both main branches of the superfamily, the ‘tetragnathoid branch’ and the ‘araneoid branch’ (Fig. 33). Not even one of the seven main araneoid lineages lacks the basal ‘*Erigone*-type’. It is a single bothrial type present in the ‘malkariods’; it is included in the shortened basal E-A sequence in the ‘symphytognathoids’ and ‘linyphioids’; and it is included in the complete E-A-T sequences in the ‘tetragnathoids’, ‘araneoids’ ‘cyatholipoids’ and ‘theridioids’. It should be emphasized that the terminal ‘*Theridion*-type’ exists only as a portion of the complete E-A-T sequences and seems but a crown of this trend. There are not a single lineage having a solely the advanced ‘*Theridion*-type’ (cf. the ‘malkariods’), and even having the shortened terminal A-T sequence (cf. the ‘symphytognathoids’ and ‘linyphioids’).

The shortened terminal A-T sequence is very rare even at the subfamily/tribe level: only two cases, in the ‘tetragnathoids’ and the ‘araneoids’, vs 12 cases of the shortened basal E-A sequence (Table 1). The subfamilies/tribes having the advanced ‘*Theridion*-type’ are not so rare (12 cases), and even somewhat more numerous than the intermediate ‘*Argiope*-type’ (nine cases), but their number is almost half as large as the number of those having the basal ‘*Erigone*-type’ (21 cases).

In addition, bothrial morphology provides additional morphological arguments in some controversial cases of araneoid taxonomy.

(1) The genera *Gaucelmus*, *Hamus*, and *Nescina* were relocated by Ramírez et al. (2022) from Nesticidae into the ‘newly enlarged Synotaxidae’, based on copulatory organ characters and molecular data. Bothrial morphology strongly supports this transfer: the relocated genera have bothria of the ‘*Erigone*-type’ or ‘*Argiope*-type’, whereas all the ‘typical nesticids’ have bothria of the ‘*Theridion*-type’ (cf. Figs 22C–E, 25A–C). It should be noted that the isolated position of the genus *Eidmannella* in the Nesticidae (termed the ‘*Eidmannella* clade’ in this study), supposed by Lehtinen and Saaristo (1980) and confirmed now by Ballarin (pers. comm. 1.07.2022), is supported by its ‘*Argiope*-type’ bothria (Fig. 25D).

(2) Almost all theridiids, from all the subfamilies, have highly uniform dome-like bothria of the ‘*Theridion*-type’ (Fig. 26A–F). A remarkable exception is the tetrad of the genera listed in the subfamily Pholcommatinae: *Carniella*, *Theonoe*, and *Robertus* have the ‘*Erigone*-type’ of bothria (Fig. 27A–C), and the *Pholcomma* has the ‘*Argiope*-type’ (Fig. 27D). Agnarsson (2004: 462) united *Pholcomma*, *Carniella*, and *Robertus* in ‘clade 35’, and agreed with Knoflach’s (1996) opinion about the close relationship between *Carniella* and *Theonoe*: “The results of this study strongly support such a placement [of *Carniella*] as sister to the clade (*Pholcomma*, *Robertus*) [...]. Knoflach (1996) suggested affinities between *Carniella* and *Theonoe* based on the absence of the male palpal tibial trichobothrium and modification of the cymbial tip [...]. *Theonoe* shares several additional features with the clade containing *Carniella*, *Pholcomma* and *Robertus* and thus Knoflach’s argument seems well founded” (Agnarsson 2004: 463). Bothrial morphology clearly confirms the particular position of the well-supported Agnarsson’s (2004) ‘clade 35’ in the family Theridiidae. It should be mentioned that according to some molecular data (Arnedo et al. 2004) *Pholcomma* and *Robertus* were considered as the sister group to the remaining theridiids.

Thus, we propose to assign to the Agnarsson’s (2004) ‘clade 35’, comprising the four genera of habitually similar small/minute ‘erigonid-like theridiids’, i.e., *Theonoe*, *Carniella*, *Robertus*, and *Pholcomma*, the rank of a separate subfamily of Theridiidae. When choosing the name for this taxon, comprising both *Theonoe* and *Pholcomma*, the Theonoeinae Simon, 1894, stat. nov. seems to be the most preferable. Theonoeae and Pholcommateae Simon, 1894 were established in the same study (Simon 1894: 586 and 589, respectively), and it allows us to discard the subfamilial name Pholcommatinae Simon, 1894, because the later taxon is well known as a taxonomic ‘trash heap’ lacking a conventional diagnosis: “The composition of this subfamily is uncertain” (Agnarsson 2004: 468). It is possible that further investigations, including molecular ones, will confirm the status of Theonoeinae as a sister group to the remaining theridiids. The bothria of theonoeines, the most plesiomorphic subfamily of the family, may be an additional morphological argument here.

(3) The micropholcommatid genus *Plectochetos* Butler, 1932 was synonymized with *Micropholcomma* Crosby & Bishop, 1927 by Forster (1959: 297) with no precise justification of this act: “A close examination of both males and females of *Plectochetoslongissimus* Butler and the structure of the respiratory system leads me to conclude that this species is also congenenc with *M*[*icropholcomma*] *caeligenus* Crosby and Bishop” (Forster 1959: 298). Surprisingly, this doubtful synonymy was never discussed further, even in the fundamental revision of Micropholcommatidae by Rix and Harvey (2010). In our opinion, however, these two genera have nothing in common, except for the apneumonic respiratory system, which is but a family character. *Plectochetos* has a large bulb, comparable in the size to the prosoma, and extremely long spiral embolus (Forster 1959: figs 64, 67, 68; Forster and Platnick 1984: fig. 369), whereas the male palp of *Micropholcomma* is so small that without a microscope may be confused with the female one (personal observations), and with short embolus shaped as an incomplete ring (Rix and Harvey 2010: figs 14A, B, 21A, B). Schütt (2003: 131), dealing in her study with *Micropholcomma* only, erroneously considered a minute male palp as the subfamily character of Micropholcommatinae: “61 *Cymbium and bulbus*: (0) normal size; (1) very small, not reaching beyond Fe I; (2) about as large as the prosoma. In Micropholcommatinae the male pedipalp is so shortened that the bulbus tip does not reach beyond the femur of the first leg, which is a synapomorphy for the subfamily” (Schütt 2003: 147). In addition to the drastically dissimilar male palps and endogynal characters such as the “super-coiled insemination ducts” in *Plectochetos* (cf. Rix and Harvey 2010: fig. 15E and fig. 15A–C), these two genera clearly differ in several somatic character, e.g., the presence of a dorsal abdominal scutum in the females of *Micropholcomma* (Rix and Harvey 2010: fig. 13A, B) and its absence in females of *Plectochetos* (Forster 1959: fig. 65). The ‘*Erigone*-type’ of bothria in *Micropholcomma* (Fig. 16A) and the ‘*Argiope*-type’ of bothria in *Plectochetos*, gen. revalid. (Fig. 16D; Forster and Platnick 1984: fig. 375) complements such list of morphological differences.

(4) Gregorič et al. (2015: 241) synonymized zygiellid genus *Parazygiella* Wunderlich, 2004 with *Zygiella* F.O. Pickard-Cambridge, 1902, based on purely molecular data. However, the detailed morphological diagnosis of *Parazygiella* by Wunderlich (2004a: 936) seems more convincing, and we consider it as a separate genus. The bothrial morphology supports such a separation too: *Zygiella* has bothria of the ‘*Argiope*-type’ (Fig. 13A), whereas the bothria of *Parazygiella*, gen. revalid. belong to the ‘*Theridion*-type’ (Fig. 13C).

(5) The male palps of the zygiellid *Zygiellaatrica* (C.L. Koch, 1845) and *Z.keyserlingi* (Ausserer, 1871) are very similar and both drastically differ from that of the type species *Z.x-notata* (Clerck, 1757), as well as from all the other *Zygiella* species (Levi 1974: 272) in the extremely elongated seta-bearing tibia and extremely enlarged hook-like paracymbium (cf. Levi 1974: figs 5, 13, and 29, respectively). Thus, a separate genus can be proposed for *Zygiellaatrica* (and *Z.keyserlingi*) based on the palp characters. The different bothria, the ‘*Argiope*-type’ in *Z.x-notata* (Fig. 13A) and the ‘*Theridion*-type’ in ‘Zygiella’ atrica (Fig. 13D) also support such a separation.

(6) Álvarez-Padilla and Hormiga (2011) could not definitely nest several tetragnathid genera into their cladogram of the family: “Inside Tetragnathinae only *Cyrtognatha* changes placement as either sister to *Tetragnatha* or sister to all other tetragnathines. [...] *Azilia* is either sister to Leucauginae with the morphology and behaviour data, or sister to all other tetragnathids with all data [mainly molecular] combined” (Álvarez-Padilla and Hormiga 2011: 728). Bothrial morphology can provide some additional arguments for such a choice. *Cyrtognatha* with its ancestral ‘*Erigone*-type’ of bothria (Fig. 9A) seems a sister group rather to “other tetragnathines” with their complete E-A-T sequence (Fig. 9B–D) than to *Tetragnatha* with its advanced ‘*Theridion*-type’ (Fig. 9E). *Azilia* with ‘*Argiope*-type’ of bothria (Fig. 9F) seems a sister group rather to Leucauginae with their shortened basal E-A sequence (Fig. 8A, B) than to “all other tetragnathids” with their complete E-A-T sequence.

## ﻿Conclusions

Three superfamilies (i.e., Nicodamoidea, Deinopoidea, and Leptonetoidea) have been proposed previously as the sister group of Araneoidea. Both Araneoidea and Nicodamidae share the following characters: (1) the ‘simplified’ trichobothrial leg pattern (no tarsal, a single metatarsal trichobothrium); (2) serrate (not plumose) setae; (3) scaled (not ridged) leg cuticle; and (4) trichobothrial bases not longitudinally ridged. These characters stated here in the set of morphological synapomorphies support the ‘purely molecular’, until now, clade Nicodamoidea + Araneoidea. So, bothrial morphology, as well as the morphology of the rest of cuticle microstructures, clearly support the nicodamoid-araneoid relation hypothesis, in contrast to both the competiting ones (i.e., deinopoid-araneoid and leptonetoid-araneoid relations).

The bothrium of nicodamids, confirmed here as a sister group of araneoids, is ‘hooded’, which indicates the polarity of this character in the Araneoidea + Nicodamidae clade. Hence, the ancestral type in the superfamily Araneoidea is recognized as a ‘hooded’ bothrium with a single well-developed transverse ridge, dividing proximal and distal plates (‘*Erigone*-type’); the advanced type is a solid dome-like bothrium without vestiges of the ridge (‘*Theridion*-type’); there are also several intermediate cases reflecting various pathways and stages of ridge reduction (all united in this study as the ‘*Argiope*-type’). The same trend in the evolution of bothrial types was described in detail by Ramírez (2014) in Dionycha.

Forster’s (1988) old hypothesis (“The reduction of the posterior hood of the bothrium is derived character which have developed apparently in parallel in many of the families”) has been confirmed. The parallel continuous sequences from the ancestral bothrial type to the advanced one through some intermediate stages are found in each of the seven main phylogenetic lineages of the superfamily Araneoidea. There is not a single lineage lacking the ancestral ‘*Erigone*-type’. It is a sole bothrial type in the ‘malkariods’, and it is the shortened basal ‘*Erigone*-type’ — ‘*Argiope*-type’ sequence in the ‘symphytognathoids’ and ‘linyphioids’. Finally, the ‘tetragnathoids’, ‘araneoids’ ‘cyatholipoids’ and ‘theridioids’ have the complete ‘*Erigone*-type’ — ‘*Argiope*-type’ — ‘*Theridion*-type’ sequences. It should be emphasised that the terminal ‘*Theridion*-type’ exists only in the complete sequences and seems but a crown of this trend.

Bothrial morphology provides additional arguments for several taxonomic acts:

- the genera *Gaucelmus*, *Hamus* and *Nescina* are relocated from nesticids to synotaxids, and the isolated position of the genus *Eidmannella* in Nesticidae (termed ‘ *Eidmannella* clade’ in this study) are confirmed; the reranking of Agnarsson’s (2004) ‘clade 35’ (*Theonoe*, *Carniella*, *Robertus* and *Pholcomma*) to the theridiid subfamily Theonoeinae Simon, 1894, stat. nov., is proposed;
- the generic independence of the micropholcommatid genus *Plectochetos*, gen. revalid. (from synonymy with *Micropholcomma*) and the zygiellid genus *Parazygiella*, gen. revalid. (from synonymy with *Zygiella*) is restored; the congenericy of *Zygiella* species *Z.atrica* and *Z.keyserlingi* with the type species *Z.x-notata* is doubted, a separated genus for the former species can be proposed, and the bothrial structures also support such a separation; the controversial position of the tetragnathid genera *Cyrtognatha* and *Azilia* in the family cladograme by Álvarez-Padilla and Hormiga (2011) is clarified.

## ﻿Additional information

### Author ORCIDs

Kirill Y. Eskov https://orcid.org/0000-0002-7953-0746

Yuri M. Marusik https://orcid.org/0000-0002-4499-5148

### Data availability

All of the data that support the findings of this study are available in the main text or Supplementary Information.

## Supplementary materials

Supplementary material 1 List of the specimens examined Data type docx https://binary.pensoft.net/file/1183715This dataset is made available under the Open Database License (http://opendatacommons.org/licenses/odbl/1.0/). The Open Database License (ODbL) is a license agreement intended to allow users to freely share, modify, and use this Dataset while maintaining this same freedom for others, provided that the original source and author(s) are credited.Kirill Y. Eskov, Yuri M. Marusik
