# Supplementary material for: ﻿A comparative morphology of trichobothrial bases in araneoid spiders and its significance for the phylogeny and system of the superfamily Araneoidea (Arachnida, Araneae)
Source: Zookeys. 2024 Nov 26;1219:1–60. doi: 10.3897/zookeys.1219.133002 (PMC11615620; doi:10.3897/zookeys.1219.133002)
Supplement: Supplementary material 1 — List of the specimens examined [file zookeys-1219-001_article-133002__-s001.docx]

**APPENDIX 1**

**List of the specimens examined**

All taxa (both araneoid and non-araneoid) are listed in alphabetical order.

Abbreviations of araneoid families and subfamilies/tribes are the same as in APPENDIX 2 “Notes on the accepted system of the superfamily Araneoidea, down to subfamilies/tribes”: families (three lowercase letters, in round brackets) and subfamilies/tribes (five lowercase letters, in square brackets). If the conventional division of the family to subfamilies/tribes is not established, we conditionally list all its members as a ‘nominative subfamily’ (marking it by the usual five lowercase letters in square brackets).

The non-araneoid taxa, not included in the cladograms, are not provided with abbreviations.

The studied specimens belong to the following institutions:

CAS – California Academy of Sciences, San Francisco, USA;

FBPC – personal collection of F. Ballarin

MCZ – Museum of Comparative Zoology, Harvard University, Cambridge, USA;

MMUE – **The Manchester Museum**, University of Manchester, UK;

QMB – Queensland Museum, Brisbane, Australia;

ZMH – Zoological Museum Hamburg, Hamburg, Germany;

ZMTU – Zoological Museum, University of Turku, Finland.

If the belonging of a specimen is not indicated, it belongs to ZMMU – Zoological Museum, Lomonosov Moscow State University, Moscow, Russia.

Anapidae **(Ana)**.

Anapidae: Anapinae **[Anapi]**.

*Acrobleps hygrophilus* Hickman, 1979

♂: *Australia*, Tasmania, Hartz Mts N.P., mixed mountain forest with *Nothofagus*, wet moss at trickle, 15.12.2015 (K.Y. Eskov).

*Crassanapis chilensis* Platnick & Forster, 1989

♂: *Chile*, Puyehue N.P., Anticura Sector, *Nothofagus* forest, under logs, 19.01.2014 (K .Eskov).

*Elanapis aisen* Platnick & Forster, 1989

♂: *Chile*, Alerce Andino N.P., *Nothofagus* forest valley, in epiphytic moss and lichens. 10-17.01.2015 (K.Y. Eskov ).

*Hickmanapis minuta* (Hickman, 1943)

♂: *Australia*, Tasmania, Hobart, Wellington Mt., subalpine thin forest, in moss hillocks, 30.11-1.12.2015 (K.Y. Eskov).

*Minanapis* sp.

♂: *Chile*, Puyehue N.P., Antilanca Sector, wet *Juncus*-*Carex* meadow, in litter, 17.01.2014 (K.Y. Eskov).

*Montanapis* sp.

♂ (ZMTU): *New Caledonia*, Thio, Ouroue (P.T. Lehtinen).

*Pseudanapis* sp. (J.Murphy det.)

♂: *Malaysia*, W. Pahang, Genting, 1000 m, moss forest, 6.02.1988 (J. Murphy).

*Sheranapis* sp.

♂: *Chile*, Alerce Costero N.P., mountain *Nothofagus* forest, in epiphytic moss and bromeliads, 23‒28.01.2015 (K.Y. Eskov).

*Sofanapis* sp.

♂: *Chile*, Alerce Costero N.P., floodland mixed forest, in epiphytic moss and bromeliads, 22-26.01.2015 (K.Y. Eskov).

*Zangherella apuliae* (Caporiacco, 1949)

♂: *Turkey*, Manisa Prov., Spil Mt, 25.05‒20.10.2019 (R. Kaya).

*Zealanapis* sp.

♀: *New Zealand*, North Isl., Manawatu-Whanganui, 139 m, *Pinus radiata* plantation, in litter, 31.01.2004 (M. Minor).

Anapidae: Gigiellinae **[Gigie]**.

*Gigiella millidgei* Rix & Harvey, 2010

♂: *Australia*, Tasmania, Mt Field N.P., Fenton L., subalpine thin forest, moss hillocks, 23.12.2015 (K .Eskov).

Anapidae: Holarchaeinae **[Holar]**.

*Holarchaea novaeseelandiae* (Forster, 1949) (J. Murphy det.)

♀ (MMUE): *New Zealand*, Westland, Moeraki L., moss scrub, 11.01.1991 (J. Murphy).

Anapidae: Taphiassinae **[Taphi]**.

*Taphiassa castanea* Rix & Harvey, 2010

♂: *Australia*, Tasmania, Mt Field N.P., Lady Barroni Falls, rainforest with fern-trees, moss cover on logs, 21.12.2015 (K .Eskov).

Anapidae: Teutoniellinae **[Teuto]**.

*Teutoniella cekalovici* Platnick & Forster, 1986

♂ (ZMTU): *Chile*, Concepcion Prov., Parque Hualpen, 8.04.1977 (T. Cekalovic [TC-70]).

Araneidae **(Ara).**

Araneidae: Araneinae **[Arane]**.

*Araneus diadematus* Clerck, 1757

♂: *Russia*, Karelia, 15 km E of Poyakonda, Belomorskaya biological station, botanical garden, 2.08.2011 (K.G. Mikhailov)

*Cyclosa conica* (Pallas, 1772)

♀: *Russia*, Moscow Area, Serpukhov Dist., Prioksko-Terrasny Res., dry *Pinus* forest, 20.06.2014 (R.R. Seifulina).

*Hypsosinga pygmaea* (Sundevall, 1831)

♂: *Russia*, Moscow Area, Serpukhov Dist., Prioksko-Terrasny Res., Sukhoi Dol, 22.05.2014 (R.R. Seifulina).

*Larinia bonneti* Spassky, 1939

♀: *Russia*, Primorsky Prov., Lazovsky Reserve, Kievka, 11.07.1981 (T. Oliger).

*Mangora acalypha* (Walckenaer, 1802)

Male: *Russia*, Moscow Area, Serpukhov Dist., Prioksko-Terrasny Res., Sukhoi Dol, 20.06.2014 (R.R. Seifulina).

*Singa hamata* (Clerck, 1757)

♂: *Russia*, Moscow Area, Serpukhov Dist., Prioksko-Terrasny Res., Protokskoye L., 22.05.2014 (R.R. Seifulina).

Araneidae: Argiopinae **[Argio]**.

*Argiope bruennichi* (Scopoli, 1772)

♀: *Azerbaijan*, Kashkachai, 27.08.1977 (P. Dunin).

Araneidae: Caerostreae **[Caero]**.

*Caerostris sumatrana* Strand, 1915

♀: *Laos*, Vientiane Prov., env. of Nam-Lik Eco-Vill., 18°36′53.18″N, 102°24′31.87″E, on web, 28.06.2017 (M.M. Omelko).

Araneidae: Cyrtarachninae **[Cyara]**.

*Chorizopes* sp. (J. Murphy det.)

♀: *Singapore*, Bukit Timah, leaf litter, 20.02.1988 (J. Murphy).

*Cyrtarachne ixoides* (Simon, 1870) (J. Murphy det.)

♀: *Spain*, Ibiza, Sta. Eulalia, riverside scrub, 17.04.1980 (J. Murphy).

Araneidae: Cyrtophorinae **[Cypho]**.

*Cyrtophora moluccensis* (Doleschal, 1857) (P.T. Lehtinen)

♀: Tonga, Nuapopu Isl., on vegetation, 13.07.1980 (S. Golovach).

Araneidae: Gasteracanthinae **[Gaste]**.

*Gasteracantha diadesmia* Thorell, 1887

♂: *Thailand*, Trat Prov., Ko Chang Archipelago, Koh Mak Isl., 23.04.2007 (A.K. Bykov).

Araneidae: Hypognatheae **[Hypog]**.

*Hypognatha* sp.1.

♂: *Peru*, Junin Region, Calabaza, 11°30'38''S, 74°49'15'' W, 2200 m, sweeping, 16‒20.09.2017 (K.Y. Eskov).

Araneidae: Micratheninae **[Mithe]**.

*Micrathena* sp.1.

♀: *Peru*, Huanuco Region, Carpish Pass, 09°41'34'' S, 76°05'06'' W, 2400 m, cloud forest, sweeping, 9.09.2017 (K.Y. Eskov).

Araneidae: Poltyeae **[Polty]**.

*Poltys* sp.

Subad.: *Australia*, Tasmania, Southwest N.P., Mystery Creek Cave, rainforest with fern-trees, sweeping, 10.12.2015 (K.Y. Eskov).

Araneidae: Testudinareae **[Testu]**.

*Melychiopharis* sp.

♂: *Peru*, Junin Region, Calabaza, 11°30'33''S, 74°50'35''W, 2500 m, cloud forest, in epiphytes 20.09.2017 (K .Eskov).

Araneidae *incertae sedis* **[Ara-is]**.

*Guizygiella* sp.

♀: *Laos*, Champasak Prov., env. of Tad E-Tu resort, 15°11' N, 106°07' E, 1.06.2013 (M.M. Omelko).

Arkyidae **(Ark), [Arkyi]**.

*Arkys alticephala* (Urquhart, 1891)

Subad.: *Australia*, Tasmania, Hobart, Wellington Mt, rainforest with fern-trees, sweeping, 28‒29.11.2015 (K.Y. Eskov).

Comaromidae **(Com), [Comar].**

*Comaroma simoni* Bertkau, 1889

♂: *Austria*, Hieflau, *Fagus* forest, 4.10.1988 (C. Kropf).

Cyatholipidae **(Cya), [Cyath].**

*Ilisoa* sp.

♂: *South Africa*, Free State, Bloemfontain, botanical garden, 29°02'59''S, 26°12'44''E, 1380 m, 11.01.2014 (Y.M. Marusik).

*Matilda* sp. 1.

♂ (QMB): *Australia*, Queensland, Brisbane, Mt Coot-tha, under rocks on ground with rudimentary web, 1.02.1980 (R. Raven & V. Davies).

*Teemenaarus silvestris* Davies, 1978 (P.T. Lehtinen det.)

♂ (ZMTU): *Australia*, East Queensland, Bulburin State Forest, 25.08.1977 (V. Davies).

*Tekella absidata* Urquhart, 1894 (J. Murphy det.)

♂: *New Zealand*, Otago, Saddle Hill, 2300 m, primary bush (J. Murphy).

Hypochilidae

*Hypochilus pococki* Platnick, 1987 (N.I. Platnick det.)

♂: *USA*, NC. Jackson Co., Caney Fork Rd., 1.2 mi N intersec. with Johns Cr. Rd., 2350', 8.09.1994 (F. Coyle).

Leptonetidae

*Leptonetela caucasica* Dunin, 1990

♂: *Russia*, Krasnodar Prov., Khosta, *Taxus* and *Buxus* forest, litter, 03‒05.2008 (Y.A. Chumachenko).

Linyphiidae **(Lin).**

Linyphiidae: Erigoninae **[Erigo]**.

*Erigone dentipalpis* (Wider, 1834)

♂: *UK*, Devon, Slapton Ley Nature Reserve, 06.2007 (D.V. Logunov).

*Lophomma vaccinii* (Emerton, 1926)

♂: *Russia*, Evenkia, Taimura R., mouth of Neptene R., *Sphagnum* hillocks at *Carex* swamp, in moss, 4.08.1982 (K.Y. Eskov).

*Minyriolus pusilus* (Wider, 1834)

♂: *Finland*, Lapland, Pallas-Yllastunturi Ntn Park, mixed forest with spruce dominating, moss and litter, 6.08.2016 (Y.M. Marusik).

*Pelecopsis mengei* (Simon, 1884)

♂: *Russia*, Evenkia, Taimura R., Chambe R. mouth, floodland *Salix* bushes, in litter, 16.08.1982 (K.Y. Eskov).

*Scutpelecopsis wunderlichi* Marusik & Gnelitsa, 2009

♂: *Russia,* North Osetia, Tsey Mt. Ridge, 4 km E of Tsey Vill., 2300 m, slope meadow, 15.9‒7.09.1985 (S. Alexeev).

Linyphiidae: Linyphiinae **[Linyp]**.

*Allomengea scopigera* (Grube, 1859)

♂: *Russia*, Kola Penins., env. of Murmansk, W coast of Kola Inlet, 3‒10.08.2010 (A.A. Nekhaeva).

*Lepthyphantes leprosus* (Ohlert, 1865)

♂: *Russia*, Moscow Area, Serpukhov Dist., Prioksko-Terrasny Res., *Pinus* forest, under bark, 14.06.2017 (R.R. Seifulina).

*Linyphia triangularis* (Clerck, 1757)

♂: *Russia*, Mordovia, Temnikov Dist., Mordovsky Reserve, Pushta Vill., 7‒17.07.2010 (E. Trushina).

*Porrhomma pygmaeum* (Blackwall, 1834)

♂: *Russia*, Kola Penins., env. of Murmansk, W coast of Kola Inlet, coastal meadow, 68°54.36'N, 33°01.54'E, 4‒11.06.2011 (A.A. Nekhaeva).

Linyphiidae: Micronetinae **[Minet]**.

*Agyneta cauta* (O. Pickard-Cambridge, 1903)

♂: *Russia*, Kola Peninsula, Murmansk vicinity, W coast of Kola Inlet, birch forest (F32), 68°54.38' N, 33°00.97' E, 4‒11.06.2011 (A.A. Nekhaeva).

*Maro pansibiricus* Tanasevitch, 2006

♂, female: *Russia*, Krasnoyarsk Area, Yenisei R., Mirnoye, 62°20' N, *Pinus sibirica* forest, in moss, 8.07.1978 (K.Y. Eskov).

*Microneta viaria* (Blackwall, 1841)

♂: *Russia*, Kola Penins., env. of Murmansk, W coast of Kola Inlet, 68°54.36'N, 33°01.19'E, 4‒11.06.2011 (A.A. Nekhaeva).

Linyphiidae: Mynogleninae **[Myngl]**.

*Haplinis mundenia* (Urquhart, 1894) (J. Murphy det.)

♂: *New Zealand*, Otago, Saddle Hill, 2300 m, primary bush, 12.1990‒01.1991 (J. Murphy).

*Parafroneta confusa* Blest, 1979 (J. Murphy det.)

♂: *New Zealand*, Otago, Saddle Hill, 2300 m, primary bush, 12.1990-01.1991 (J. Murphy).

Linyphiidae: Stemonyphantinae **[Stemo]**.

*Stemonyphantes lineatus* (Linnaeus, 1758)

♂: *Russia*, Moscow Area, Serpukhov Dist., Prioksko-Terrasny Res., wet meadow, 21.05.2014 (R.R. Seifulina).

*Stemonyphantes sibiricus* (Grube, 1861)

♀: *Russia*, Kamchatka, Kamchatsky Pass, *Betula* forest, in litter, 22.08.1987 (A.V. Tanasevich).

*Weintrauboa insularis* (Saito, 1935)

♂, ♀: *Russia*, Sakhalin Area, env. of Yuzhno-Sakhalinsk, Dolina Turistov, 18.10.1985 (A.M. Basarukin).

Malkaridae **(Mal).**

Malkaridae: Malkarinae **[Malka]**

*Malkara* sp.

♀ (MMUE): *Australia*, Queesnsland, Cape Tribulation, coastal rain forest, 24.07.1992 (J. Murphy).

Malkaridae: Sternoidinae **[Stern]**.

*Chilenodes australis* Platnick & Forster, 1987

♂: *Chile*, Chiloe Isl., Senda Darwin Biological Station (15 km E of Ancud), 41°52'56''S, 73°40'04''W, lowland forest of *Nothofagus* and *Podocarpus*, in litter, 3‒4.01.2014 (K.Y. Eskov).

Malkaridae: Tingotinginae **[Tingo]**.

*Tingotingo* sp.

♀ (MMUE): *New Zealand*, Fiordland, Hollyford, 300 m, leaf litter, 6.02.1986 (J. Murphy).

Micropholcommatidae **(Mic)**.

Micropholcommatidae: Micropholcommatinae **[Micph]**.

*Austropholcomma* sp.

♂: *Australia*, Tasmania, Hartz Mts N.P., Osborn L., floodland *Nothofagus* forest, wet moss at trickle, 17.12.2015 (K.Y. Eskov).

*Micropholcomma bryophilum* (Butler, 1932)

♂: *Australia*, Tasmania, Southwest N.P., Mystery Creek Cave. Boggy forest in spring valley, in wet moss, 10‒12.12.2015 (K.Y. Eskov).

*Micropholcomma parmata* Hickman, 1944

♂: *Australia*, Tasmania, Southwest N.P., Mystery Creek Cave, rainforest with fern-trees, in moss hillocks, 10‒14.12.2015 (K .Eskov).

*Plectochetos longissimus* Butler 1932

♂: *Australia*, Tasmania, Lake St Clair N.P., Mt. Rufus, 1000 m, subalpine boggy forest, *Sphagnum* with *Richaea scoparia*, 6‒7.12.15 (K.Y. Eskov).

*Tricellina gertschi* (Forster & Platnick, 1981)

♀: *Chile*, Alerce Andino N.P., valley *Nothofagus* forest, in epiphytic moss and lichens. 10‒17.01.2015 (K.Y. Eskov).

Micropholcommatidae: Textricellinae **[Textr]**.

*Eperiella alsophila* Rix & Harvey, 2010

♀: *Chile*, Tierra del Fuego. Lago Blanca, 54°02'S, 68°56'W, 115 m, *Sphagnum* bog at lake bank, tops of *Sphagnum magellanicum* hillocks (berlese samples), 4.11.2015 (A. Haustov).

*Epigastrina fulva* (Hickman, 1945)

♂: *Australia*, Tasmania, Hobart, Wellington Mt., rainforest with fern-trees, in epiphytic moss and lichens, 29.11.2015 (K.Y. Eskov)

*Eterosonycha alpina* Butler, 1932

♂: *Australia*, Tasmania, Hobart, Wellington Mt., subalpine thin forest, in moss hillocks, 30.11‒1.12.2015 (K.Y. Eskov).

*Normplatnicka chilensis* Rix & Harvey 2010

♂: *Chile*, Tierra del Fuego. Lago Blanca 54°02'S, 68°56'W, 115 m, *Sphagnum* bog at lake bank, tops of *Sphagnum magellanicum* hillocks (berlese samples) 4.11.2015 (A. Haustov).

*Raveniella hickmani* (Forster, 1959)

♂: *Australia*, Tasmania, Hobart, Wellington Mt., subalpine thin forest, wet moss at trickle, 30.11.2015 (K.Y. Eskov)

*Rayforstia vulgaris* (Forster, 1959)

♂: *New Zealand*, South Isl., Rainbow Ski Area, 1717 m , alpine zone, in litter under carpet grass, 28.01. 2017 (M. Minor).

Mimetidae **(Mim)**.

Mimetidae: Gelanorinae **[Gelan]**.

*Gelanor* sp. 1.

♂ (MMUE): *Costa Rica*, Guanacaste Prov., La Pacifica R., riverine forest, 21.08.1983 (J. Murphy).

*Gelanor* sp. 2.

♂ (USNM): *Ecuador*, Orellana, Tiputini Biodiversity station, nr Yasuni National Park, Erwin Transect – T/1 Sta.1: 00°37'55''S, 76°08'39''W, 220‒250 m, 5.02.1999 (T.L. Erwin et al.).

Mimetidae: Mimetinae **[Mimet]**.

*Australomimetus tasmanensis* (Hickman, 1929)

♂: *Australia*, Tasmania, Mt Field N.P., Russel Falls, rainforest, sweeping on fern-trees, 20-27.12.2015 (K.Y. Eskov).

*Ero furcata* (Villers, 1789)

♂: *Russia*, Moscow Area, Serpukhov Dist., Prioksko-Terrasny Res., Protokskoye L., 10.07.2014 (R.R. Seifulina).

Mimetidae: Oarcinae **[Oarci]**.

*Oarces reticulatus* (Nicolet, 1849)

♀: *Chile*, Alerce Andino N.P., *Nothofagus-Podocarpus* forest, in litter, 5‒11.01.2014 (K.Y. Eskov & R. Rakitov).

Mysmenidae **(Mys)**.

Mysmenidae: Mysmeninae **[Mysme]**.

*Microdipoena* sp.

♂: *Vietnam*, Dong-Nai, Ma-Da forest, litter sample, 6.06.1995 (T. Sergeeva).

*Mysmena leucoplagiata* (Simon, 1880)

♂: *Turkey*, Ankara Prov., Kizilkahamam Dist., nr Camlidere Town, 40°32.709'N, 32°30.547'E, 964 m, 28.05.2009 (Y.M. Marusik).

Mysmenidae: Mysmenopsinae **[Mysps]**.

*Isela inquilina* (Baert & Murphy, 1987) (J. Murphy det.)

♂: *Kenya*, Coast, Kilifi, garden, 9.08.1980 (J. Murphy).

*Mysmenopsis tengellacompa* Platnick, 1993 (N.I. Platnick det.)

♂: *Costa Rica*, Puntarenas, Monteverde, 1500 m, scrub, 26.08.1983 (J. Murphy).

Nephilidae **(Nep), [Nephi].**

*Nephila* sp.

♀: *Thailand*, 01.2007 (A.V. Bykov).

*Nephilengys malabarensis* (Walckenaer, 1841)

♂: *Vietnam*, Thanh Hoa Prov., Lang Trang Cave, 10‒20.05.2022 (K. Tarasenko).

Nesticidae **(Nes)**

Nesticidae: ‘*Eidmannella* clade’ **[Eidma]**.

*Eidmannella pallida* (Emerton, 1875) (J. Murphy det.)

♀: *USA*, Maryland, Cambridge, woods, Golden Hill, 31.07.1981 (J. Murphy).

Nesticidae: Nesticellini **[Necel]**.

*Nesticella terrestris* (Yaginuma, 1970)

♂: *Russia*, Kuril Islands, Shikotan Isl., Krabozavodskoe Vill., 43°19'61''N, 146°45'44''E, 10‒18.09.1997 (Y.M. Marusik).

Nesticidae: Nesticini **[Nesti]**.

*Aituaria pontica* (Spassky, 1932)

♂: *Ukraine*, Chernovtsy, in cellar, 25.02.2017 (V.L. Voloshin & M.M. Fedoryak).

*Daginesticus mamajevae* (Marusik, 1987)

♂: *Georgia*, Legodekhi Reserve, cellar, 26.07.2012 (Y.M. Marusik)

Nicodamidae

*Litodamus hickmani* Harvey, 1995

♂: *Australia*, Tasmania, Hobart, Wellington Mt., rain forest with fern-trees, under stones and logs, 29.11.2015 (K.Y. Eskov).

Pararchaeidae **(Par), [Parar].**

*Anarchaea corticola* (Hickman, 1969)

♂: *Australia*, Tasmania, Hobart, Wellington Mt., subalpine thin forest, in moss hillocks, 30.11‒1.12.2015 (K.Y. Eskov).

*Flavarchaea lulu* Rix, 2006

♂: *Australia*, Tasmania, Mt Field N.P., Nichols L., subalpine *Eucalypt* forest, in moss hillocks, 23.12.2015 (K.Y. Eskov).

Physoglenidae **(Phy).**

**Physoglenidae:** Pahorinae **[Pahor]**.

*Pahora murihiku* Forster, 1990 (J. Murphy det.)

♂: New Zealand, Otago Saddle Hill, 2300 m, primary bush, 12.1990‒01.1991 (J. Murphy).

Physoglenidae: Physogleninae **[Physo]**.

*Physoglenes puyehue* Platnick, 1990

♂: *Chile*, Puyehue N.P., Anticura Sector, *Nothofagus* forest, under logs, 19.01.2014 (K.Y. Eskov).

*Physoglenes* sp.

♀: *Chile*, N.P. Alerce Costero, *Spagnum* bog, in moss, 24.01.2015 (K.Y. Eskov).

*Tupua bisetosa* Platnick, 1990

♂: *Australia*, Tasmania, Southwest N.P., Mystery Creek Cave, rainforest with fern-trees, under stones and logs, 12‒14.12.2015 (K.Y. Eskov).

Pimoidae **(Pim), [Pimoi].**

*Nanoa enana* Hormiga, Buckle & Scharff, 2005 (G. Hormiga det.).

♂ (CAS, paratype, 9118963): *USA*, Oregon, Eastern Siskiyou Mts, Jackson Co., Ashland Ranger Dist., Rogue River National Forest, Ashland watershed, south of Ashland, 42°8′58.0′N− 122°42′24.9′W, 978 m, pitfall trap, 29.09–13.10.1998 (R.W. Peck et al*.*).

*Pimoa rupicola* (Simon, 1884) (P. Pantini det.)

♂: *Italy*, Emilia-Romagna, Ravenna, Brisighella, 368 m, [T407001 Vena del Gesso Romagnola Monte Rontana, loc.Rontana, pitfal traps 16.05.‒18.07.2020 (R. Fabbri).

Symphytognathidae **(Sym), [Symph]**.

*Anapistula* sp.

♀ – *China*, Yunnan, Mengla B.G., hard leaved litter, 2.10.1987 (P.T. Lehtinen).

*Symphytognatha globosa* Hickman, 1931

♂: *Australia*, Tasmania, Mt Field N.P., Russel Falls, rainforest, sweeping on fern-trees, 20‒27.12.2015 (K .Eskov).

*Symphytognatha* sp.

♂: *Peru*, Junin Region, Calabaza. 11°30'33''S, 74°50'35''W, 2500 m, cloud forest, in epiphytes 20.09.2017 (K.Y. Eskov).

Synaphridae **(Syn), [Synap].**

*Cepheia longiseta* (Simon, 1881) (J. Murphy det.)

♀: *Portugal*, Algarve, Monte Gordo, sand dunes, 3.04.1971 (J. Murphy).

*Synaphris lehtineni* Marusik, Gnelitsa & Kovblyuk, 2005

♀ (paratype): *Ukraine*, Crimea, Feodosiya Dist., Karadagh Reserve, nr Syuryu-Kaya Mt., S exposed steppe slope, c. 30–40°, under stones, 10.10.2003 (V.A. Gnelitsa).

Synotaxidae **(Syt), [Sytax].**

*Gaucelmus* sp.

♀: USA, Texas, Hays Co., San Marcos, Ezell's Cave, 27.05.1948 (A. Flury).

*Hamus cornutus* Lin, Ballarin & Li, 2016 (F. Ballarin det.)

♂ (FBPC): *China*, Guangxi, Nanning, Longan, 23.03855°N, 107.754167°E,11.07.2011 (Wang).

*Nescina* sp.1 (F. Ballarin det.)

♀ (FBPC): *Taiwan*, Lanyu Isl., Dongqing, 40 m, 22.02987°N, 121.5707°E, humid forest litter, 15.VI.2019 (F. Ballarin)

*Nescina* sp.2

♂ (ZMTU): *Philippines*, Luzon Isl., Quezon National Park, Mirano 11.07.11.1977 (P.T Lehtinen).

*Synotaxus* sp.

♀ (USNM): *Ecuador*, Orellana, Tiputini Biodiversity station, nr Yasuni N.P., Erwin Transect – 00°37'55''S, 76°08'39''W, 220‒250 m, 8.11.1999 (T.L. Erwin et al.)

*‘Tekellina’ araucana* Marusik, Eskov & Ramírez, 2022

♂: *Chile*, Alerce Andino N.P., *Nothofagus* forest along river, in epiphytic moss and lichens, 10‒17.01.2015 (K .Eskov).

*Tekellina sadamotoi* Yoshida & Ogata, 2016

♂: *Japan*, Tokyo, Musasashi-murrayama-shi, Kishi, 26.12.2016 (T. Ichikawa).

Tetragnathidae **(Tet).**

Tetragnathidae: Diphyainae **[Diphy]**.

*Chrysometa alajuela* Levi, 1986 (F. Alvares det.).

♀ (MCZ): *Costa Rica*, P.N. (ACLA) Estacion Pittier, 09°01'N, 82°5'’W, 1800 m, 8‒11.06.1995 (G. Hormiga).

*‘Diphya’ wulingensis* Yu, Zhang & Omelko, 2014

♂: *Russia*, Primorsky Prov., Lazovski Reserve, Korpad Camp, 43°16′N 134°08′E, 6–9.08.1998 (Y.M. Marusik).

Diphyainae gen.sp.

♂: *Vietnam*, Bac Thai, Thai Nguen Plateau, 35 km N An Khe, Buon Luoi, tropical rain forest, in litter, 9.01.1981 (T. Sergeeva).

Tetragnathidae: Leucauginae **[Leuca]**.

*Leucauge granulata* (Walckenaer, 1841)

♂: *Tonga*, Nuapopu Isl., on vegetation, 13.07.1980 (S.I. Golovach).

*Metleucauge dentipalpis* (Kroneberg, 1875)

♀ (ZMMU, paratype ТА – 1014): *Turkestan*, Urgut (Fedchenko Expedition).

Tetragnathidae: Metainae **[Metai]**.

*Meta menardi* (Latreille, 1804)

♂: *UK*, Cheshire, Maeplc., in dark cгulvert, 1.05.1964 (D. Mackie).

*Metellina mengei* (Blackwall, 1869)

*♂: UK*, Cheshire, Alderley Edge, 5°18'N, 2°14'E, 7.07.2002 (D.V. Logunov).

*Metellina merianae* (Scopoli, 1763)

*♂: UK*, Devon, Slapton Ley Nature Reserve, 19.06.2007 (D.V. Logunov).

Tetragnathidae: Nanometinae **[Nanom]**.

*Nanometa* sp.

♂: *Australia*, Tasmania, Southwest N.P., Mystery Creek Cave, rainforest, sweeping on fern-trees, 11‒13.12.2015 (K .Eskov).

*Orsinome sarasini* (Berland, 1924)

♂: *Australia*, Tasmania, Southwest N.P., Mystery Creek Cave, rainforest with fern-trees, sweeping, 10.12.2015 (K .Eskov).

*Pinkfloydia* sp.

Subadult (ZMTU): *Indonesia*, Kalimantan, Timur Kutal Dist., Lempack Tomahmorah, rain forest, 24.10.1979 (P.T. Lehtinen).

Tetragnathidae: Tetragnathinae **[Tetra]**.

*Allende* sp. (G. Hormiga det.).

♂ (MCZ): *Chile*, VIII Region del Biobio, 4.2 km N of Contulmo, Lago Lanalhue, Cabanas Licalhue (Contulmo Cabins), 37.97945109°S; 73.24627331°W, 41 m. 13.11.2014 (G. Hormiga et al.).

*Cyrtognatha pachygnathoides* (O. Pickard-Cambridge, 1894) (D. Dimitrov det.).

♀ (MCZ): *Costa Rica*, Cerro Pittier, 1700 m, 8‒11.06.1995 (G. Hormiga).

*Mollemeta edwardsi* (Simon, 1904) (G. Hormiga det.).

♀ (MCZ): *Chile*, X Region de Los Lagos, Parque Nacional Puehue, Sector Aguas Calientes, sendero Pionero (Puehue NP 3) 40.7378708°S, 72.31094889°W, 538 m, 17.11.2014 (G. Hormiga et al.).

*Pachygnatha listeri* Sundevall, 1830

♂: *Russia*, Moscow Area, Serpukhov Dist., Prioksko-Terrasny Res., mixed forest, 20.06.2014 (R.R. Seifulina).

*Tetragnatha extensa* (Linnaeus, 1758)

♂: *Russia*, Chukotka, upper Osinovaya River (tributary of Belaya River), 10‒15.07.1989 (Y.M. Marusik).

Tetragnathidae: *incertae sedis* **[Tet-is]**.

*Azilia* sp. (G. Hormiga det.).

♀ (MCZ): *Panama*, Panama Prov, P.Nac. Altos de Campana, 8°41'00.4''N, 79°55'47.4''W, 895 m, 14‒19.VI.2007 (D. Dimitrov & G.Hormiga).

Theridiidae **(Thr).**

Theridiidae: Argyrodinae **[Argyr]**.

*Argyrodes* sp.

♀: *Peru*, Junin Region, Pichiquia, 11°23'07''S, 74°06'05''W, 500 m, valley forest, sweeping 26-28.09.2017 (K .Eskov).

Theridiidae: Hadrotarsinae **[Hadro]**.

*Phycosoma* sp.

♂: *Peru*, Junin Region, Calabaza, 11°30'38''S, 74°49'15''W, 2200 m, moss hillock at riverbank, 17.09.2017 (K .Eskov).

*Euryopis flavomaculata* (C. L. Koch, 1836).

♂: *Russia*, Moscow Area, Serpukhov Dist., Prioksko-Terrasny Res., Protokskoye L., 31.05.2014 (R.R. Seifulina).

Theridiidae: Latrodectinae **[Latro]**.

*Crustulina guttata* (Wider, 1834)

♂, ♀: *UK*, Suffolk, Minsmere R., marsh, 21.09.1972 (J. Crocker).

*Latrodectus tredecimguttatus* (Rossi, 1790)

♀: *Turkmenistan*, Repetek Reserve, 9.04.1982 (V. Krivokhatsky).

Theridiidae: Pholcommatinae **[Pholc]**.

*Carniella nepalensis* Tanasevitch & Marusik, 2020

♂: *Nepal*, Taplejung Dist., above Yamputhin (= Yamphudin), ca 27°27'N, 88°00'E, 1800–2000 m, left bank of Kabeli Khola, bushes, open forest, 27–29.04.1988 (J. Martens & W. Schawaller).

*Glebych minutissimus* Eskov & Marusik, 2021

♂: *Peru*, Junin Region, Calabaza, 11°30'33''S, 74°50'35''W, 2500 m, cloud forest, in epiphytes 20.09.2017 (K.Y. Eskov).

*Pholcomma gibbum* (Westring, 1851) (J.Crocker det.)

♀: *UK*, Whiteford Burrows, Gower Peninsula (south Wales), marram tussocks, 7.07.1964 (J. Crocker).

*Robertus lividus* (Blackwall, 1836)

♂: *Russia*, Moscow Area, Serpukhov Dist., Prioksko-Terrasny Res., Protokskoye L., *Pinus* forest, sweeping 10.06.2014 (R.R. Seifulina).

*Theonoe minutissima* (O. Pickard-Cambridge, 1879)

♂: *UK*, Surrey, Chobhom Common, in *Sphagnum*, 15.03.1970 (J. Crocker).

Theridiidae: Phoroncidieae **[Phorn]**.

*Phoroncidia* sp.

♂: *Australia*, Tasmania, Hobart, Wellington Mt., rainforest with fern-trees, sweeping, 28‒29.11.2015 (K.Y. Eskov).

Theridiidae: Spintharinae **[Spint]**.

*Episinus angulatus* (Blackwall, 1836)

♂: *Russia*, Moscow Area, Serpukhov Dist., Prioksko-Terrasny Res., Protokskoye L., mixed forest, sweeping 7.06.2014 (R.R. Seifulina).

Theridiidae: Theridiinae **[Thrid].**

*Theridion tinctum* (Walckenaer, 1802)

♂: *UK*, England, Cheshire, Alderley Edge, 5°18'N, 2°14'E, 7.07.2002 (D.V. Logunov).

*Theridion varians* Hahn, 1833

♂: *Russia*, Moscow Area, Serpukhov Dist., Prioksko-Terrasny Res., *Sphagnum* bog, sweeping, 7.06.2014 (R.R. Seifulina).

Theridiosomatidae **(Ths)**.

Theridiosomatidae: Epeirotypinae **[Eptyp]**.

*Naatlo* sp.

♂: *Peru*, Junin Region, 10 km SE from Satipo, Catarata la Resistencia, slope forest, sweeping 30.09.2017 (K.Y. Eskov).

Theridiosomatidae: Ogulninae **[Oguln]**

*Ogulnius* sp.

♂: *Peru*, Junin Region, Pichiquia, 11°23'07''S, 74°06'05''W, 500 m, valley forest, sweeping 29.09.2017 (K.Y. Eskov).

Theridiosomatidae: Platoninae **[Plato]**

*Plato troglodita* Coddington, 1986 (N. Dupérré det.)

♂ (ZMH): *Ecuador*, Tena Prov., Comunidad de Guayusa Loma, en Caverna de Rocas en la Propiedad del Sr. Leodan, 00.996499°S, 77.739784°W, 720 m, cave, 15.09.2021 (E.E. Tapia, Leodan).

Theridiosomatidae: Theridiosomatinae **[Thsom]**.

*Theridiosoma radiosum* (McCook, 1881) (J. Murphy det.)

♀: *USA*, Virginia, Montgomery Co., Craig Creek, 20.07.1978 (J. Murphy).

*Epilineutes* sp.

♂: *Peru*, Junin Region, 10 km SE from Satipo, Catarata la Resistencia, slope forest, sweeping 23.09.2017 (K.Y. Eskov).

Uloboridae

*Myagrammopis* sp.

♀: *Peru*, Junin Region, Calabaza, 11°30'33''S, 74°50'35''W, 2500 m, cloud forest, in epiphytes 19.09.2017 (K .Eskov).

*Zozis* sp.

♀: *Peru*, Junin Region, Pichiquia, 11°23'07''S, 74°06'05''W, 500 m, valley forest, sweeping 26-28.09.2017 (K.Y. Eskov).

Zygiellidae **(Zyg), [Zygie]**.

*Leviellus caspicus* (Simon, 1889)

♂: *Uzbekistan*, Kyzylkumsky Reserve, tugai forest, 4.04.1985 (D.V. Logunov).

*Leviellus stroemi* (Thorell, 1870)

♀: *Russia*, Mordovia, Mordovsky Rezerve, Pushta Vill, 10.06.‒31.08.2011 (E. Trushina).

*Parazygiella dispar* (Kulczyński, 1885)

♀: *Russia*, Khabarovsk Prov., Bolshe-Khekhtsyrski Reserve, 1990 (S.I. Golovach).

*Zygiella x-notata* (Clerck, 1757)

♂: *Russia*, Moscow Area, Serpukhov Dist., Prioksko-Terrasny Res., Sukhoi Dol, 22.05.2014 (R.R. Seifulina).

*‘Zygiella’ atrica* (C.L. Koch, 1845)

♂: *UK*, Dorset, Stodland Neath, 30.8.1968 (D. Mackie).
